# Improving KT tools and products: development and evaluation of a framework for creating optimized, Knowledge-activated Tools (KaT)

**DOI:** 10.1186/s43058-020-00031-7

**Published:** 2020-05-08

**Authors:** Monika Kastner, Julie Makarski, Leigh Hayden, Yonda Lai, Joyce Chan, Victoria Treister, Kegan Harris, Sarah Munce, Jayna Holroyd-Leduc, Ian D. Graham, Sharon E. Straus

**Affiliations:** 1 North York General Hospital, Centre for Research and Innovation, 4001 Leslie Street, Toronto, Ontario M2K 1E1 Canada; 2 Institute of Health Policy, Management and Evaluation, University of Toronto, Health Sciences Building, 155 College Street, Suite 425, Toronto, ON M5T 3M6 Canada; 3 Li Ka Shing Knowledge Institute of St. Michael’s hospital, University of Toronto, 30 Bond Street, Toronto, ON M5B 1W8 Canada; 4 University Health Network, Toronto, Ontario Canada; 5 Departments of Medicine and Community Health Sciences, Cumming School of Medicine, University of Calgary, Calgary, Alberta Canada; 6 School of Epidemiology and Public Health, Clinical Epidemiology Program, University of Ottawa and Ottawa Hospital Research Institute, Ottawa, Ontario Canada; 7 Department of Medicine, University of Toronto, Toronto, Canada

**Keywords:** Knowledge translation, KT tools, Interventions, Framework, Knowledge user needs, Delphi study, Survey

## Abstract

**Background:**

Positive impacts of quality improvement initiatives on health care and services have not been substantial. Knowledge translation (KT) strategies (tools, products and interventions) strive to facilitate the uptake of knowledge thereby the potential to improve care, but there is little guidance on *how* to develop them. Existing KT guidance or planning tools fall short in operationalizing all aspects of KT practice activities conducted by knowledge users (researchers, clinicians, patients, decision-makers), and most do not consider their variable needs or to deliver recommendations that are most relevant and useful for them.

**Methods:**

We conducted a 3-phase study. In phase 1, we used several sources to develop a conceptual framework for creating optimized Knowledge-activated Tools (KaT) (consultation with our integrated KT team, the use of existing KT models and frameworks, findings of a systematic review of multimorbidity interventions and a literature review and document analysis on existing KT guidance tools). In phase 2, we invited KT experts to participate in a Delphi study to refine and evaluate the conceptual KaT framework. In phase 3, we administered an online survey to knowledge users (researchers, clinicians, decision-makers, trainees) to evaluate the potential usefulness of an online mock-up version of the KaT framework.

**Results:**

We developed the conceptual KaT framework, and iteratively refined it with 35 KT experts in a 3-round Delphi study. The final framework represents the blueprint for what is needed to create KT strategies. Feedback from 201 researcher, clinician, decision-maker and trainee knowledge users on the potential need and usefulness of an online, interactive version of KaT indicated that they liked the idea of it (mean score 4.36 on a 5-point Likert scale) and its proposed features (mean score range 4.30–4.79).

**Conclusions:**

Our findings suggest that mostly Canadian KT experts and knowledge users perceived the KaT framework and the future development of an online, interactive version to be important and needed. We anticipate that the KaT framework will provide clarity for knowledge users about *how* to identify their KT needs and *what* activities can address these needs, and to help *streamline* the process of these activities to facilitate *efficient* uptake of knowledge.

Contributions to the literature

- The KaT framework is the first evidence- and theory-based resource that consolidates the disparate and varied sources of information related to knowledge translation (KT) activities into one framework and builds on existing guidance resources by focusing on the specific steps and processes that are needed to optimize the rigorous and efficient development of KT strategies.
- We have operationalized the steps and processes needed to ensure that rigorous and appropriate methods are applied in the creation of KT strategies regardless of what KT activity(ies) are considered to respond to knowledge user needs (i.e. whether the need is to develop, implement, evaluate, disseminate, sustain or scale the KT strategy and/or to ensure that an integrated KT approach is considered).
- The KaT framework represents the infrastructure for building an online, user-responsive platform that will be knowledge user centred and could optimize how we move research evidence into practice and policy more efficiently and rigorously with the best potential to improve care.

## Background

Knowledge translation or implementation science (hereon referred to as KT) is important to ensure optimized uptake of knowledge and decision-making. Given the less than optimal implementation of research evidence to inform practice and policy [1–3], positive impacts on health care and services have not been substantial with an average of 10–15% improvement observed in most quality improvement initiatives [4]. The quality and efficiency of patient care remains sub-optimal [3] with only about 55% of patients receiving recommended care [5]. Failure to use research evidence to inform practice and policy can also lead to practice variation [1–3] and can contribute to research waste if findings are poorly implemented or not at all [4, 6–8]. KT can respond to these challenges by optimizing the uptake of knowledge for different populations across different contexts and can support practice and policy decision-making for a wide range of knowledge users (e.g. researchers, patients, caregivers, clinicians, policy-/decision-makers). In particular, there has been a rapid growth in the development of KT tools, products and interventions (hereon referred to as KT strategies), which strive to present evidence in clear, concise, and user-friendly formats to facilitate the uptake of knowledge thereby increasing the potential to improve patient care and health services and delivery.

There is a wide range of KT strategies created for different KT purposes such as to inform or change clinical practice, behaviour, policy, organizations or systems and to influence research, academic and educational communities. KT strategies can target clinicians (e.g. clinical practice guidelines, computerized decision support systems, educational strategies, reminder systems, audit and feedback), decision-makers (e.g. policy briefs) or be patient-oriented (e.g. decision aids, eHealth/mHealth apps, educational videos). There are also strategies that support KT practice such as to help generate knowledge (e.g. knowledge synthesis tools [9]), implement or disseminate knowledge (e.g. dissemination planning tools [10]), assess the readiness for change within an organization [11] and create a plan for end-of-grant/-project KT [12].

There are many sources of information available to help create KT strategies whether the aim is to improve care through optimized translation of knowledge to practice/policy or to advance the science of KT and implementation. However, there is very little guidance on *how* to develop these KT strategies. None of existing KT guidance or planning tools provide a “one-stop-shop” to guide knowledge users on *all* aspects of their desired KT practice activities (i.e. whether this might involve developing, disseminating, implementing, evaluating, sustaining or scaling KT strategies or using an integrated KT approach in these activities) [13–25]. Integrated KT is an approach to conducting research that involves all relevant knowledge users in every aspect of research from setting objectives, designing and executing the study, interpreting findings through to generating a plan to disseminate the results [26]. Additionally, most KT guidance tools do not consider a customized approach to generate an action plan that can respond to the variable needs of different knowledge users in different contexts or to deliver this knowledge in the language and format that might be the most relevant and useful for them. The objectives of our study were to (1) iteratively develop and evaluate an evidence-informed conceptual framework (for use by researchers, clinicians, decision-makers and trainees) that outlines the processes required to rigorously and efficiently create KT strategies with the best potential for impact and (2) to evaluate the potential usefulness of the framework (conceptualized as an online, user-responsive platform) with researcher, clinician, decision-maker and trainee (i.e. graduate students, clinical residents) knowledge users.

## Methods

### Design overview

We used an iterative, three-phase approach to address our objectives. In phase 1, we created a conceptual framework representing the steps and processes required to rigorously and efficiently create KT strategies; we call this the Knowledge-activated Tools (KaT) framework. In phase 2, we invited KT and implementation science experts to participate in a Delphi study to further refine and evaluate the conceptual KaT framework. In phase 3, we administered an online survey to knowledge users (researchers, clinicians, decision-makers, trainees) to evaluate the potential usefulness of an online mock-up version of the KaT framework.

### Phase 1: Development of the conceptual KaT framework

#### Data sources

We used a wide array of data sources to create the KaT framework. First, we consulted with our integrated KT team consisting of experts in KT and health services (*n* = 5), intervention/tool development and design (*n* = 2) and clinicians (*n* = 3) across Canada, the USA and the UK. Second, we used the knowledge creation and dynamic action steps of the Knowledge-to-Action (KTA) model [14] and the Medical Research Council (MRC) framework for complex interventions [27] as the foundation to build the steps to creating KT strategies. Third, we used findings of a systematic review of the effectiveness of KT interventions for older adults with multimorbidity [28]. For each article that was included in this systematic review (*n* = 25), we extracted data on any processes and steps taken to create the intervention: (1) the use of any evidence to support the selected KT intervention, (2) any knowledge generation activity such as a knowledge synthesis to inform the evidence for its development, (3) if the intervention considered any theory to guide the development (e.g. the KTA model [14], the Theoretical Domains Framework [TDF] [29], which helps to identify factors that influence behaviour change), (4) if the determinants of implementation or intervention use was assessed, (5) any usability testing or pilot evaluation, (6) if there was any planned implementation strategy and (7) any assessment of the intervention’s sustainability potential. We used these data to fill gaps or confirm identified process steps from the KTA and MRC frameworks. Fourth, we performed a literature review and document analysis [30] to identify existing processes, models, frameworks and guidance and planning tools for creating KT strategies. This involved searching published and unpublished literature (in 2016) from MEDLINE, Google Scholar, the Cochrane Effective Practice and Organisation (EPOC) taxonomy [31], the KT web pages of the Canadian Institutes of Health Research (CIHR) [32] and KT networks, conferences and journals in Canada and the USS (KT Canada [33], Alberta SPOR KT Platform [34], KT Connects [35], KT Canada Scientific Meeting [36], Annual Science of Dissemination and Implementation conference [37]).

#### Data extraction and analysis

Data were extracted from each identified report using a standardized form on the report name, author, year and KT strategy characteristics (name, description, purpose, knowledge user targets, development process and its steps and processes). We used an online visual mapping platform (MindMeister®) to iteratively develop the conceptual framework and undertake the iterative framework development process to identify the specific steps and processes required to create KT strategies. With each iteration of the framework, we sought feedback from our integrated KT team to clarify steps/processes and to confirm the logic and sense of the framework organization and its content. We also conducted a mapping exercise, whereby a sub-set of our team (MK, YL, VT, JC) reviewed existing KT strategies and documented their purpose and features in duplicate, and compared their similarities and differences with our proposed conceptual KaT framework to highlight areas in the creation of KT strategies that are well understood vs. underrepresented or understudied areas that require further investigation and understanding. Additional file 1: Appendix A shows the first iteration of the KaT framework.

### Phase 2: Evaluation of the KaT framework—Delphi study

#### Design overview

We used a modified Delphi approach [38] and the reporting criteria for Delphi studies as outlined by Diamond et al. [39] to evaluate and refine the KaT framework with KT experts. We aimed to establish consensus on the framework’s organization and structure (i.e. its components, sub-components and design), clarity (understandability) and content comprehensiveness (i.e. whether it includes all the steps that are needed to rigorously create KT strategies) and whether they thought it would be perceived as useful by different knowledge users (researchers, clinicians and decision-makers). We obtained ethics approval through the St. Michael’s Hospital research and ethics board. Informed consent was implied with the individual’s acceptance of their invitation to participate in the online Delphi surveys.

#### Population and recruitment

We used a purposive sampling strategy [40] to recruit an internationally representative panel of KT experts (i.e. experience with applying rigorous methods to conduct KT research and/or advancing KT science [i.e. having studied the methods of KT]). Potential participants were identified from (1) a list of KT experts known or suggested by our integrated KT team, (2) publicly available lists of individuals who have presented at KT and health services research conferences and meetings in Canada and the USA (KT Canada [33], Alberta SPOR KT Platform [34], KT Connects [35], KT Canada Scientific Meeting [36], Annual Science of Dissemination and Implementation conference [37] and the Canadian Association for Health Services and Policy Research [CAHSPR] [41]), (3) snowball sampling whereby identified KT experts were asked to suggest others, and (4) scanning the *Implementation Science* journal [42] to identify authors of highly cited articles published from 2010 onwards. Individuals who accepted the personalized email invitation were automatically directed to an online survey platform (SimpleSurvey^TM^) to complete Round 1 of the Delphi study. Our target sample size was 20 participants as evidence recommends that 15–20 participants are needed to reach consensus appropriately and feasibly [43].

#### Delphi survey development and administration

We anticipated needing up to three rounds of ratings to reach consensus on the conceptual KaT framework and its domains and sub-domains. The round 1 survey consisted of a combination of 7-point Likert scale and open-ended (free text) questions with 3 sections to assess (1) the overall clarity (understandability), organization (structure), comprehensiveness and appropriateness of the framework and its domain labels; (2) the framework content (description of each of its domains and sub-domains); and (3) participant demographics (Additional file 1: Appendix B). The survey was pilot-tested with three KT experts to ensure the appropriateness and understandability of questions. We created a 5-min introductory video (embedded within the Delphi survey) to provide context and clarity about how the KaT framework was developed and organized, an approach we have previously used successfully [44].

#### Delphi process

After round 1, Delphi participants received a summary of their individual results (the proportions of ranked items at each point on the scale highlighting their ratings in the context of aggregated ratings of the panel), along with a revised version of the KaT framework. This feedback response system allowed participants to consider their initial ranking in the context of the aggregated scores of their peers [38, 45]. Round 2 involved a teleconference-based discussion among panel members to clarify framework items that did not reach consensus in round 1; two meetings were held to maximize opportunities for participation by all panel members. The sessions involved a facilitator (MK) reviewing the aggregated ratings for each section of the framework and prompting discussion to resolve disagreements. Comments from the first teleconference group were relayed to the second group to ensure that everyone’s feedback was considered in final decisions. In round 3, Delphi participants were invited to rate any remaining non-consensus items and to provide feedback on the revised framework using an online survey. We used Dillman’s total design survey method to maximize participation (up to two reminders/round, 1 week apart [46]). Consensus to include an item was defined a priori as a mean score of ≥ 5 out of 7 on a Likert scale (1 = not at all agree; 7 = very much agree) by ≥ 80% of Delphi participants; and to exclude an item if it was scored < 5 out of 7 by ≥ 80% of participants. Items that did not reach consensus were summarized along with their corresponding qualitative data (if provided) and used to help the panel with reassessment in subsequent rounds. The decision to make a change to the KaT framework after each round was iterative and based on the consensus score to include/exclude as well as the consistency of data between quantitative ratings and supporting qualitative. This involved two researchers who checked if qualitative data from open-ended questions confirmed quantitative scores (e.g. positive, confirmatory statements for items that reached consensus to include) or contradicted quantitative scores (e.g. negative statements about an item that reached consensus).

#### Outcomes and analysis

The primary outcome was the degree of consensus reached among the KT expert panel on KaT framework domains and their sub-domains. Data analysis involved both quantitative (summary statistics) and qualitative data. Quantitative data were analysed using descriptive statistics to show percent agreement among panellists for each rated item and to assess central tendency (means, median and their standard deviations) [38, 47, 48] and level of dispersion using the interquartile range (IQR), which provides an assessment of the extent of agreement between participants [45] (i.e. IQR of 0, 1 and 2 are considered high, good and poor consensus; respectively [49]). Qualitative responses were analysed using content analysis [50], whereby one reviewer read and coded the data using themes that corresponded with components of the KaT framework (KH) and a second reviewer checked the first reviewer’s coding (JM). Any disagreements were resolved by a third reviewer (MK) and team discussions.

### Phase 3: Survey of KT knowledge users of a conceptual version of the KaT framework

#### Survey development

We used the Checklist for Reporting Results of Internet E-Surveys (CHERRIES) [51] to guide the development of our knowledge user survey. Our goal was to elicit the perceptions of KT and quality improvement (QI) knowledge users (researchers, clinicians, decision-makers, trainees) of the potential usefulness of an online, user-responsive version of the KaT framework. We defined perceived usefulness according to Davis et al. as “the degree to which a person believes that using a particular system would enhance their job performance” [52]. Knowledge user survey questions were iteratively developed with the help of our integrated KT team. Questions included a combination of Likert-type and open-ended questions to assess the features of a conceptual, online version of KaT (section 1); its perceived usefulness (section 2); and participant demographics (section 3) (Additional file 1: Appendix C).

To help participants respond to questions about the potential usefulness of an online, user-responsive version of the KaT framework, we created mock-up web pages of the interactive domains of the framework (i.e. *Explore*, *Action plan*) (see Additional file 1: Appendix D). For example, in an online version of the framework, knowledge users would begin with an “exploration” process, which would prompt inputs about their KT purpose, scope and resources. These inputs would then be used to generate a comprehensive *Action plan* tailored to their identified needs. We provided screenshots of the *Explore* and *Action plan* web page mock-ups alongside survey questions to provide context to what they were responding to.

To facilitate the identification of the type of KT strategy that may exist and/or the one(s) that best match knowledge users’ purpose, scope and resources, we created a draft table of existing KT strategies that are organized by knowledge user target audience mapped to seven broad KT purpose categories adapted from the CIHR guidance on KT planning [12] (see Additional file 1: Appendix E). We designed this *KT Purpose Category* table to help knowledge users to explore available KT strategies and to help them decide whether the creation of a new KT strategy is warranted or an existing strategy can be used or adapted; this may reduce the potential for duplication of effort and research waste. Our future work to create an online version of the KaT framework will include a repository of KT strategies (building on those listed in the *KT Purpose Category* table [12]) whereby each strategy will be tagged for methodological quality and relevance (to user needs).

#### Participants and recruitment

We used a multistage, purposive sampling strategy to identify our participants [40]: researchers, clinicians and decision-makers who were involved in KT or QI activities and research. This strategy involved an emphasis on administering the survey to all our target knowledge user groups followed by targeted recruitment of groups with lower response rates [44]. We built a database of potential knowledge users through publicly available listservs, websites, conference proceedings and journals: KT Canada [33], Alberta SPOR KT Platform [34], KT Connects [35], Centre for Quality Improvement and Patient Safety (C-QUIPS) [53], KT Scientific Meeting [36], the Annual Science of Dissemination and Implementation conference [37], the Canadian Association for Health Services and Policy Research (CAHSPR) [41], Knowledge Utilization Colloquium [54]; *Implementation Science* [42], *Canadian Health Policy* journal [55], the *Journal of Health Services Research & Policy* [56]; and the *International Journal of Health Policy and Management* [57]. The survey was distributed using email invitations with a link to an online survey platform (SimpleSurvey^TM^).

#### Outcomes and data analysis

The primary outcome was perceived usefulness of the proposed features of an online version of the KaT framework by KT knowledge users measured by a validated perceived usefulness scale based on the technology acceptance model [52]. Quantitative analysis of Likert-type survey questions involved descriptive statistics (proportions for categorical and means for continuous outcomes variables) and qualitative analysis of responses from open-ended question involved content analysis [50]; data were coded for themes by one reviewer (JM) and checked for agreement by a second (MK).

## Results

### Phase 1: Development of the KaT framework

**Fig. 1:**
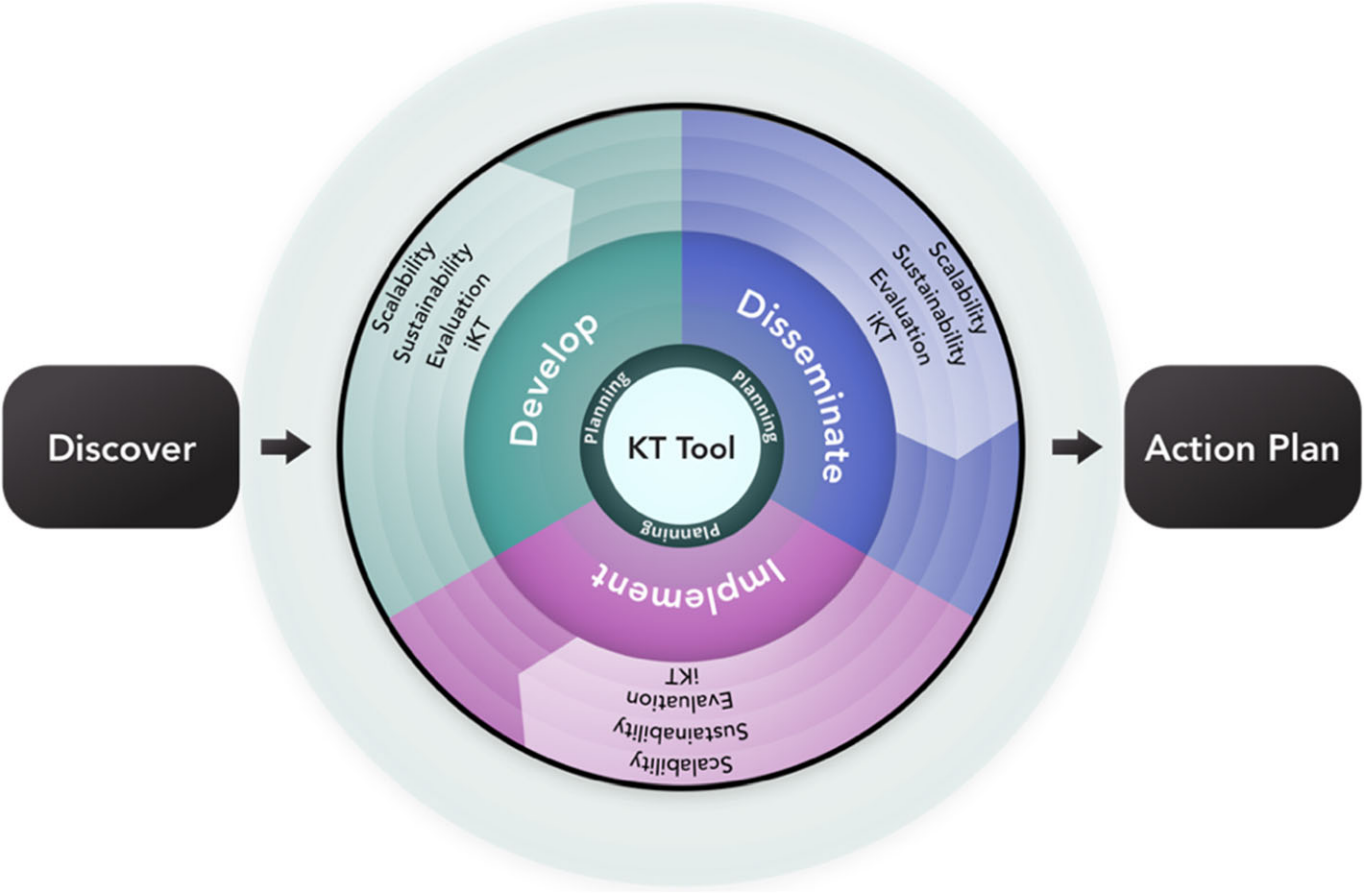
Pre-Delphi version of the Knowledge-activated Tools (KaT) framework

Table 1 shows the breakdown of the KaT framework domains (*n* = 9) and their sub-domains (*n* = 37), which were informed by 126 unique articles that were identified from our iterative evidence searching; the evidence supporting each of these are detailed in Additional file 1: Appendices F–I. The evidence-informed framework (Fig. 1) that outlines the steps and processes for the rigorous creation of KT strategies is in Table 1.

**Table 1:** Composition of the conceptual KaT framework

Domain	Sub-domains and their elements	No. of articles
**Discover**	1. Identify gap, need, problem 2. Identify purpose of KT tool/product 3. Define scope	3
**Develop**	1. Engage relevant stakeholders 2. Identify evidence base of chosen KT tool/product 3. Select theoretical basis for development or adaptation of KT tool/product 4. Develop or adapt a functioning prototype using user-centred design 5. Conduct usability evaluation of the KT tool/product	36
**Implement**	Engage relevant stakeholders and establish partnerships to: 1. Identify the implementability of the KT tool/product 2. Develop implementation plan 3. Monitor and evaluate the implementation of the KT tool or product 4. Organize and document findings—consider using a tool development and evaluation reporting criteria to guide this process	63
**Disseminate**	1. Engage stakeholders for all steps 2. Determine the goals of the dissemination and uptake 3. Design dissemination plan (i.e. end-of-grant KT plan) 4. Monitor and evaluate dissemination and uptake	9
**Action plan**	1. Summary of outputs from Discovery 2. Action plan according to the needs, purpose, scope and context of the user 3. A description of how iKT, Evaluation, Sustainability and Scalability fit within the Action plan 4. Suggested timelines (overall and for each section of the Action plan) 5. References and links to sources of action recommendations outlined in the Action plan 6. Templates relevant to Action plan items 7. Instructions manual on how to use the Action plan and templates	Informed by the steps and processes of the complete framework
**Impact drivers**		
**Integrated KT (iKT)**	1. Engage relevant stakeholders and establish partnerships throughout all the steps 2. Develop an iKT plan 3. Monitor and Evaluate the iKT plan that was implemented	12
**Sustainability**	1. Engage stakeholders throughout all steps of sustainability assessment 2. Identify the purpose of sustainability 3. Develop a sustainability plan 4. Monitor and evaluate sustainability	14
**Scalability**	1. Identify scale-up objectives and scope 2. Identify scale-up team 3. Ensure that the KT tool is ready for scale-up 4. Develop a scale-up plan 5. Monitor and evaluate	8
**Evaluation**	1. Evaluation steps are embedded within each domain (Develop, Implement, Disseminate) and impact driver (iKT, Sustainability, Scalability)	Not applicable

The KaT framework begins with the *Discovery* stage, which allows knowledge users to explore and identify their specific KT purpose and problem. For example, to increase family physicians’ awareness of a diabetes guideline or to increase self-management of older adults with multimorbidity. During the *Discovery* stage, users can also define their scope in terms of their stakeholders, context, and resources, and to identify the existing knowledge base related to their problem (e.g. does knowledge need to be generated such as via a systematic review?) (Additional file 1: Appendix F). The *Discovery* stage flows into the central part of the KaT framework, which includes three broad KT actions that we have identified as important in the creation and uptake of KT strategies: *Develop or adapt*, *Disseminate*, and *Implement*. This is a high-level representation only, as there are many sub-domains within each of these broad domains (see Additional file 1: Appendix G). There are four rings encircling the central part of the KaT framework that we call “Impact drivers” (*Integrated KT*, *Evaluation*, *Sustainability*, and *Scalability* (Additional file 1: Appendix H)) since they are determinants of KT effect or knowledge use and therefore are important to consider across any or all of developing, implementing or disseminating KT strategies, and all should be considered early in the process. The core of the framework illustrates that the goal is to create a “KT tool” (i.e. a KT strategy). The *Action plan* represents the recommended plan for action that could be generated from the *Discovery* stage (Additional file 1: Appendix I).

### Phase 2: Delphi study with KT experts

The Delphi study was carried out between January and April 2017. Of 112 KT experts who were identified and invited to participate in the Delphi study, 55 responded and 35 participated in round 1 (online survey), 19 participated in round 2 (teleconference discussions) and 26 participated in round 3 (online survey). Reasons for non-participation were no time (*n* = 15), not a KT expert (*n* = 2) and not interested (*n* = 3). The demographic characteristics of Delphi participants are in Table 2. The majority of KT experts were Canadian (91%) from Ontario (49%), Québec (20%), and Alberta (14%) having multiple roles (e.g. researcher/scientist and/or professor and/or clinician). Sixty-three percent of participants had 6–15 years of KT experience with expertise in KT practice (74%) or science (60%) in the areas of implementation (74%), Integrated KT (71%), dissemination (60%), sustainability (26%), and scalability (17%). More than half of participants were involved in developing a KT framework or model, and the most frequently used in their work were reported to be the KTA model (84%) [14], Rogers Diffusion of Innovations (61%) [58] and the TDF (42%) [29].

**Table 2:** Characteristics of KT experts who participated in the Delphi study

Characteristic	Round 1 (*n* = 35)	Round 2 (*n* = 19)	Round 3 (*n* = 26)
**Country**			
Canada			
Ontario	17 (49%)	12 (63%)	14 (56%)
Québec	7 (20%)	3 (16%)	6 (24%)
Manitoba	1 (3%)	1 (5%)	1 (4%)
Alberta	5 (14%)	3 (16%)	3 (12%)
British Columbia	1 (3%)	-	-
Newfoundland	1 (3%)	-	-
Australia	2 (6%)	-	-
USA	1 (3%)	-	1 (4%)
Role*			
Researcher, Scientist and/or Assistant/Associate/Full Professor	45	22	32
Clinician	7	3	6
Other	7	4	3
**Years of KT and implementation science experience**			
1–5 years	10 (29%)	6 (32%)	7 (28%)
6–10 years	16 (46%)	8 (42%)	11 (44%)
11–15 years	6 (17%)	3 (16%)	4 (16%)
> 15 years	3 (9%)	2 (11%)	3 (12%)
KT expertise*			
KT practice or science	47	26	34
Implementation	26	15	16
Dissemination	21	12	14
Integrated KT (iKT)	25	15	20
Sustainability	9	8	8
Scalability	6	5	5

*Participants had multiple roles and KT expertise

#### Delphi round 1 (*n* = 35)

Of 55 framework items assessed via an online survey, 16 items (29%) did not reach consensus to include (percent agreement range 54–77%) (Additional file 1: Appendix J). Table 3 shows the percent agreement and the central tendency and spread of scores. The panel indicated that the organization of the overall KaT framework made sense (agreement 83%) and was comprehensive (agreement 89%), but it was not a good reflection of the framework’s intended purpose (i.e. to guide the rigorous creation of KT strategies) (agreement 74%). The panel felt that researchers may find the framework useful (agreement 80%) but not health care providers (66%) or policy- or decision-makers (63%). Concerns were related to the complexity of the framework for use by non-KT experts, the theoretical nature of the framework, and that it may be too time-consuming to use.

**Table 3:** Delphi study with KT experts: results of round 1

KaT Framework Domain	Domain factor	N	Mean (SD)	Median	IQR†	Percent agreement to include‡
**DISCOVER**	Important to include the DISCOVER domain in the overall KaT framework	35	6.7 (0.70)	7.0	0	97%
	It makes sense for the DISCOVER domain to feed into the central part of the KaT framework	35	6.3 (0.94)	6.0	1	91%
	Knowledge users will find the DISCOVER domain useful.	35	6.1 (1.09)	6.0	2	91%
	DISCOVER is an appropriate label	35	5.0 (1.40)	5.0	2	**71%**
	The 3 sub-domains of DISCOVER (and their elements) *make sense*	33	6.2 (0.70)	6.0	1	94%
	The 3 sub-domains of DISCOVER (and their elements) are *comprehensive*	33	5.9 (1.04)	6.0	1	88%
**DEVELOP**	Important to include in the overall KaT framework	35	6.5 (0.81)	7.0	1	86%
	Knowledge users will find it useful.	35	5.7 (1.53)	6.0	2	**74%**
	The 5 sub-domains of DEVELOP (and their elements) *make sense*	33	6.2 (0.93)	6.0	1	91%
	The 5 sub-domains of DEVELOP (and their elements) are *comprehensive*	33	6.0 (0.88)	6.0	0	91%
**DISSEMINATE**	Important to include in the overall KaT framework	35	6.3 (0.86)	7.0	1	94%
	Knowledge users will find it useful.	35	5.6 (1.50)	6.0	3	**71%**
	The 4 sub-domains (and their elements) *make sense*	33	6.2 (0.75)	6.0	1	91%
	The 4 sub-domains (and their elements) are *comprehensive*	33	6.2 (0.76)	6.0	1	94%
**IMPLEMENT**	Important to include in the overall KaT framework	35	6.5 (0.81)	7.0	1	94%
	Knowledge users will find it useful	35	6.0 (1.43)	7.0	2	83%
	The 5 sub-domains (and their elements) *make sense*	32	6.0 (1.01)	6.0	1	91%
	The 5 sub-domains (and their elements) are *comprehensive*	32	6.0 (1.02)	6.0	0	91%
**3 BROAD DOMAINS**	The three domains represented in the KaT framework is comprehensive in terms of capturing what is important to consider in the creation and uptake of KT tools	35	5.54 (1.5)	6.0	2	80%
**IMPACT DRIVERS**	The label “Impact Drivers” appropriately conveys these four concepts	35	5.4 (1.29)	6.0	1	80%
	The order in which the four impact drivers are represented *make sense*	35	5.2 (1.63)	6.0	2	**71%**
	The placement of the four impact drivers clearly illustrates that they should be considered across each of the three broad domains of the framework (i.e., develop, disseminate, implement)	35	5.4 (1.58)	6.0	2	**74%**
	**Integrated KT (iKT)**					
	It makes sense to include iKT as one of the impact drivers	35	6.1 (1.23)	6.0	1	91%
	The 3 sub-domains of iKT (and their elements) *make sense*	32	6.4 (0.72)	6.0	1	97%
	The 3 sub-domains of iKT (and their elements) are *comprehensive*	32	6.3 (0.74)	6.0	1	97%
	**EVALUATION**					
	It makes sense to include EVALUATION as one of the impact drivers	35	6.1 (1.33)	7.0	1	91%
	The representation of EVALUATION across the *3 domainsmake sense*	32	5.8 (1.30)	6.0	2	88%
	The representation of EVALUATION across the *4 impact driversmake sense*	32	5.7 (1.40)	6.0	2	88%
	EVALUATION is included in all of the areas of the KaT framework that should consider evaluation	31	6.0 (0.82)	6.0	2	97%
	**SUSTAINABILITY**					
	It makes sense to include SUSTAINABILITY as one of the impact drivers	35	6.2 (0.83)	6.0	1	94%
	The 4 sub-domains (and their elements) of SUSTAINABILITY *make sense*	32	6.1 (0.70)	6.0	1	97%
	The 4 sub-domains (and their elements) of SUSTAINABILITY are *comprehensive*	32	6.0 (0.88)	6.0	1	97%
	**SCALABILITY**					
	It makes sense to include SCALABILITY as one of the impact drivers	35	6.2 (0.86)	6.0	1	91%
	The 5 sub-domains (and their elements) of SCALABILITY *make sense*	32	5.7 (1.15)	6.0	1	88%
	The 5 sub-domains (and their elements) of SCALABILITY are *comprehensive*	32	5.9 (1.02)	6.0	0	91%
**CORE**	The core is important to include as part of the overall KaT framework	35	6.0 (1.43)	6.0	1	83%
	The placement of the CORE clearly illustrates that a KT tool is the ultimate goal and end product resulting from using the KaT framework	35	5.7 (1.62)	6.0	2	**69%**
**PLANNING**	PLANNING, which encircles the core, is important to include as part of the overall KaT framework	35	5.2 (1.61)	6.0	3	**60%**
	The placement of PLANNING clearly illustrates that a plan can be generated for each or all of the three broad domains of the KaT framework (i.e., develop, disseminate, implement)	35	4.9 (1.72)	5.0	3	**54%**
**ACTION PLAN**	The ACTION PLAN is important to include as part of the overall KaT framework	35	6.6 (0.77)	7.0	1	97%
	Researchers will find it useful	35	5.7 (1.33)	6.0	3	**74%**
	Health care providers will find it useful	35	5.8 (1.20)	6.0	3	**77%**
	Policy or decision makers will find it useful	35	5.7 (1.18)	6.0	3	**74%**
	It’s clear that the ACTION PLAN will be an output resulting from the use of the KaT framework	35	6.0 (1.38)	6.0	1	91%
	The 7 outputs (and their elements) of the ACTION PLAN *make sense*	32	5.9 (1.12)	6.0	2	91%
	The 7 outputs (and their elements) of the ACTION PLAN are *comprehensive*	32	5.9 (1.11)	6.0	2	91%
**OVERALL KaT framework**	The overall framework is clear (i.e., easy to understand or interpret)	35	5.3 (1.33)	6.0	1	**77%**
	The organization makes sense	35	5.6 (1.34)	6.0	1	83%
	The KaT framework is a good reflection of its intended purpose (i.e., to guide the rigorous and efficient creation of KT tools)	35	5.7 (1.30)	6.0	2	**74%**
	The KaT framework is comprehensive (i.e., it covers the important areas that need to be considered in the creation of KT tools and products)	35	5.9 (1.10)	6.0	1	89%
	Researchers will find it useful	35	5.8 (1.20)	6.0	2	80%
	Health care providers will find it useful	35	5.3 (1.40)	6.0	2	**66%**
	Policy or decision makers will find it useful	35	5.3 (1.40)	6.0	2	**63%**
**TABLE of existing KT tools**	Knowledge users will find the TABLE of existing KT tools organized by targets useful	35	5.4 (1.60)	6.0	3	**63%**
	Knowledge users will find the TABLE of existing KT tools mapped to purpose categories useful	35	5.4 (1.60)	6.0	3	**69%**

^**†**^IQR 0 = high consensus, IQR 1 = good consensus, IQR 2 = poor consensus

^‡^Percent agreement to include item = score of ≥ 5 out of 7 by ≥ 80% of panel (consensus) or < 5 out of 7 by < 80% of panel (non-consensus)

Domain items that did not reach consensus to include by < 80% of panel are bolded

#### Delphi round 2 (*n* = 19)

The 16 items that did not reach consensus in round 1 were discussed by the panel in two teleconferences (Additional file 1: Appendix K). In particular, the panel discussed items related to the potential usefulness of the KaT framework to different knowledge users. The panel found this assessment challenging because they perceived that the back-end of each framework domain (which contains a lot of content and evidence; Additional file 1: Appendices F–I) would be too complex to use by knowledge users. We explained that although the KaT framework in itself can be used by knowledge users to guide their actions (i.e. via the representation of all the steps needed to create KT strategies), they would not be interacting with the actual evidence tables informing the framework domains and sub-domains. These evidence tables will be utilized in the future development of an online, interactive version of the KaT framework to inform its back-end content and logic.

#### Delphi round 3 (*n* = 26)

Of 14 items (carried forward from round 2) that were assessed in an online survey, all reached consensus to include (mean percent agreement 93%; range 80–100%). Table 4 shows the percent agreement and the central tendency and spread of Likert scale scores across these items.

**Table 4:** Delphi study with KT experts: results of round 3

KaT Framework Domain	Domain factor	Mean (SD)	Median	IQR^**†**^	Percent agreement to include^‡^
**EXPLORE**	EXPLORE is an appropriate label	6.6 (0.50)	7.0	1	100%
**Central component of the KaT framework** (*DEVELOP*, *DISSEMINATE*, *IMPLEMENT*)	The new placement of the 3 domains makes sense (i.e. DISSEMINATE and IMPLEMENT on either side of the core, and DEVELOP placed below)	5.4 (1.5)	6.0	2	80%
	I like the brief definition provided for DISSEMINATE (i.e. share knowledge) and IMPLEMENT (i.e. apply knowledge)	6.6 (0.58)	7.0	1	100%
**IMPACT DRIVERS**	I like the new visual representation of the IMPACT DRIVERS (i.e. their font colours match their respective domain colour)	5.9 (0.99)	6.0	1	85%
	The placement of the IMPACT DRIVERS (i.e. they are repeated at each of the three domain sectors) clearly illustrates the idea that they should be considered regardless of whether the goal is to DEVELOP, DISSEMINATE and/or IMPLEMENT a KT tool	5.9 (1.2)	6.0	1	88%
	I like the new placement of EVALUATION to convey the idea that it applies to all domains and impact drivers	6.1 (0.77)	6.0	1	96%
**CORE**	The placement of the CORE clearly illustrates that the KT tool is the ultimate goal and end product resulting from using the framework	6.1 (0.91)	6.0	1	96%
**OVERALL KaT framework**	The overall framework is clear (i.e. easy to understand or interpret)	5.9 (0.95)	6.0	1	92%
	The organization of the framework makes sense	5.9 (0.99)	6.0	2	92%
	The framework is comprehensive—it covers the important areas that need to be considered in the creation and uptake of KT tools and products (i.e. EXPLORE, DEVELOP, DISSEMINATE, IMPLEMENT, IMPACT DRIVERS, EVALUATE, ACTION PLAN)	6.2 (0.97)	6.0	1	92%
**Perceived usefulness of the KaT framework to knowledge users**	I like the idea of an online, interactive platform that can be used by a wide range of knowledge users to create KT tools and products	6.4 (0.70)	6.5	1	100%
	There is potential that researchers will find the online, interactive KaT platform useful in creating KT tools and products	6.4 (0.80)	6.5	1	96%
	There is potential that health care providers will find the online, interactive KaT platform useful in creating KT tools and products	5.7 (1.0)	6.0	2	92%
	There is potential that policy-makers will find the online, interactive KaT platform useful in creating KT tools and products	5.6 (1.2)	6.0	1	88%

*SD* standard deviation, *IQR* interquartile range

^**†**^IQR 0 = high consensus, IQR 1 = good consensus, IQR 2 = poor consensus

‡Percent agreement to include item = score of ≥ 5 out of 7 by ≥ 80% of panel (consensus) or < 5 out of 7 by < 80% of panel (non-consensus)

#### Final KaT framework

**Fig. 2:**
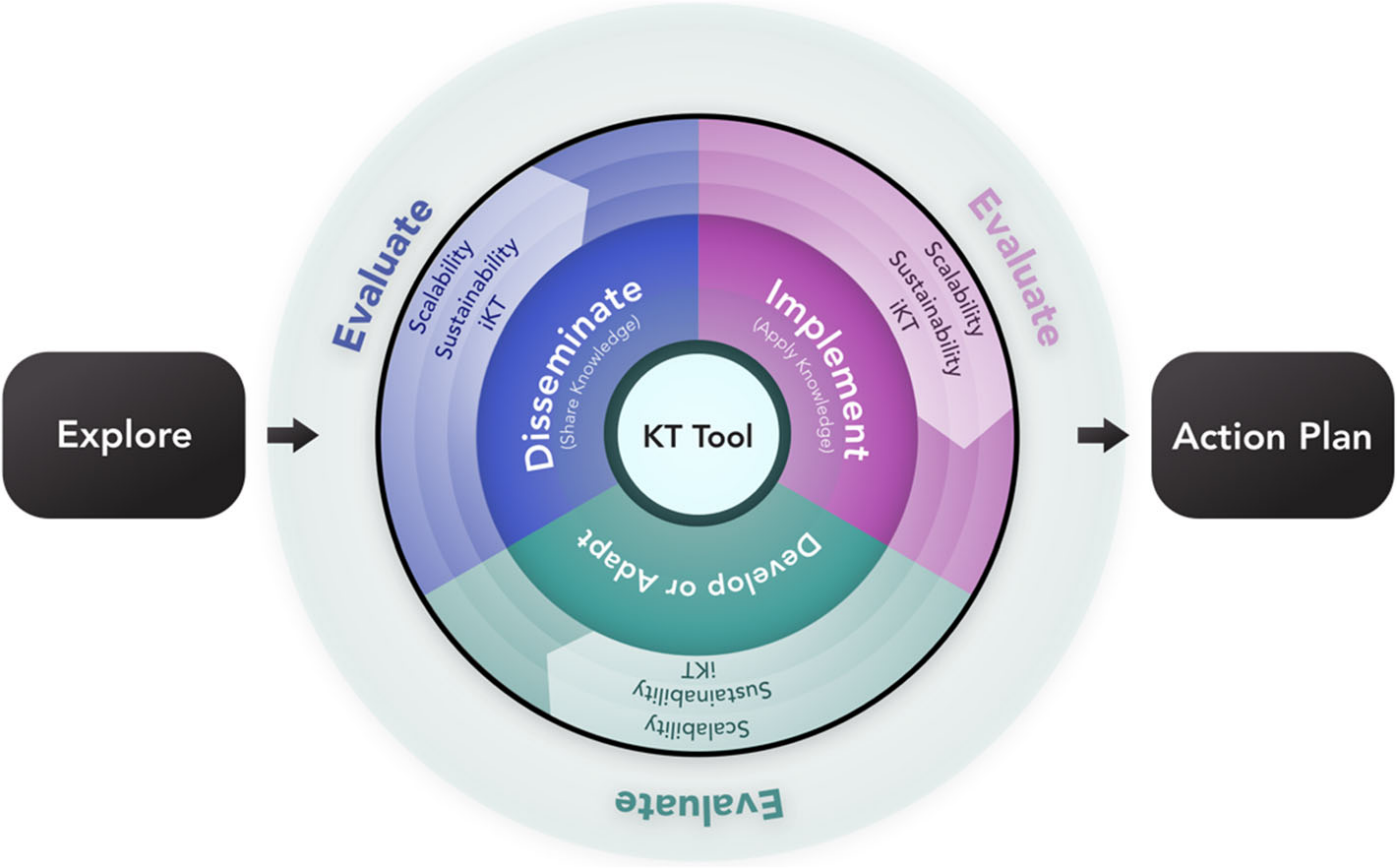
The final Knowledge-activated Tools (KaT) framework

The final version of the KaT framework (Fig. 2) was informed iteratively using three rounds of ratings and discussions with our KT experts. The following changes were made: (1) the label for the *Discover* domain was changed to *Explore*; (2) the placement of the 3 central domains were re-organized, and a brief definition was added for *Disseminate* (share knowledge) and *Implement* (apply knowledge); (3) the *Evaluate* impact driver was removed and placed in the outermost arc of the central framework to convey the idea that evaluation applies to all domains and impact drivers; (4) the placement of the remaining impact drivers (i.e. *Integrated KT*, *Sustainability*, *Scalability*) was repeated at each of the three domain sectors to highlight that they should be considered regardless of whether the KT purpose is to *Develop*, *Disseminate*, and/or *Implement* a KT strategy; and (5) *Planning*, which encircled the core of the framework was removed. The *Action plan*, which flows from the central component of the framework, represents the recommended plan of action that could be generated from the *Explore* stage including how each impact driver might fit within the overall customized plan.

### Phase 3: Knowledge user survey

The survey was initially launched in February 2018 (closing in March 2018); and re-launched to capture additional knowledge users in September 2018 (closing in October 2018); two reminders were sent to non-respondents during each launch period. Of 1750 potential participants that were invited, 254 individuals responded and 201 completed the survey (response rate 12%). Table 5 shows the demographic characteristics of survey participants (*n* = 201). The majority of respondents were researchers (59%), clinician scientists (20%) or decision-makers (13%) working in university (56%), hospital (22%) or government (8%) settings. Their primary areas of expertise were in health services (25%), KT or Implementation science (22%), medicine (15%), public health (14%), psychology (6%) and nursing (4%).

**Table 5:** Characteristics of knowledge user survey respondents (*n* = 201)

Characteristic	***N*** (%)
**Gender**	
Women	132 (65.7)
Men	66 (32.8)
Prefer not to answer	3 (1.5)
**Age range (years)**	
25–34	22 (10.9)
35–44	50 (24.9)
45–54	63 (31.3)
55–64	46 (22.9)
65–74	19 (9.5)
75–84	1 (0.5)
Primary role*	
Researcher or scientist	119 (59)
Clinician scientist	40 (20)
Decision-maker (managers, directors, clinicians, funders, policy-makers)	26 (13)
Knowledge user	10 (5)
Graduate student	4 (2)
Other	2 (1)
**Setting of employment (*n*= 195)**	
University or College	110 (56.4)
Hospital	43 (22.1)
Government	15 (7.7)
Not for profit agency or organization, foundation, non-government organization	10 (5.1)
Research institute	6 (3.1)
Other	11 (5.6)
**Primary area of expertise**	
Health services	51 (25.4)
KT/Implementation science	44 (21.9)
Medicine	30 (14.9)
Public health	28 (13.9)
Psychology	11 (5.5)
Nursing	8 (4.0)
Policy	3 (1.5)
Epidemiology	3 (1.5)
Technology	3 (1.5)
Allied health	2 (1.0)
Engineering	2 (1.0)
Pharmacy	2 (1.0)
Other	14 (7.0)

*We used the first respondent entry to identify participant primary role and area of expertise; most participants indicated as having multiple roles and expertise

#### Perceptions of an online version of KaT and its potential usefulness

Table 6 shows the mean scores on a 5-point Likert scale of participants’ perceptions of the online version of KaT. Survey respondents perceived the mock-up of the *Explore* web page as important for providing knowledge users with the opportunity to identify their KT purpose (mean 4.79 out of 5; SD 0.431). They liked the proposed feature that would allow users to select options from a series of drop-down menus to generate a KT purpose statement (mean 4.46; SD 0.624), and liked the web page overall (mean 4.54; 0.557) (Additional file 1: Appendix L). For the mock-up of the *Action plan* web page, respondents liked that it would provide customized information to users (mean 4.65; SD 0.546) and found the idea of a repository of existing KT strategies a useful feature of the proposed online KaT framework (mean 4.55; SD 0.640) (Additional file 1: Appendix M) and indicated that it was a good idea to provide quality (mean 4.41; SD 0.743) and relevance (mean 4.30; SD 0.776) ratings for KT strategies in this repository. Respondents also indicated that providing the option for users to learn about *evaluating* (mean 4.57; SD 0.606), *sustaining* (mean 4.45; SD 0.720) and *scaling* (mean 4.42; SD 0.696) their KT strategy as well as to learn how an *Integrated KT* approach could be applied (mean 4.38; SD 0.719) was important to include in the *Action plan*. Overall, respondents liked the idea of an online version of KaT (mean 4.36; SD 0.635). In terms of its potential usefulness, respondents perceived the online version of KaT as *slightly likely* to enable them to accomplish their tasks more quickly (mean 5.033; SD1.132), improve their work performance (mean 4.837; SD 1.072), increase their productivity (mean 4.724; SD 1.032), enhance the effectiveness of their work (mean 5.078; SD1.099) or to make it easier to do their work (mean 5.093; SD 1.165) and would *quite likely* find the online version of KaT useful for their work overall (mean 5.485; SD 1.044).

**Table 6:** Knowledge user survey: responses about the conceptual Knowledge-activated Tools (KaT) platform

KaT domain (sub-domain)	Mean (SD) on a 5-point Likert scale
**EXPLORE page of the conceptual KaT platform**	
It’s important to provide the opportunity for knowledge users to identify their KT purpose	4.79 (0.431)
I like the idea of selecting options from a series of drop-down menus to generate a KT purpose statement	4.46 (0.624)
Overall, I like the idea of the conceptual Explore page	4.54 (0.557)
**ACTION PLAN page of the conceptual KaT platform**	
I like the idea that the Action plan would provide customized information to platform users	4.65 (0.546)
I would find the table of existing KT tools a useful feature of the KaT platform	4.55 (0.640)
It’s a good idea to show the quality* rating for each of the suggested KT tools	4.41 (0.743)
It’s a good idea to show the relevance* rating for each of the suggested KT tools	4.30 (0.776)
It’s important to provide platform users with an option to develop a new KT tool if they wish	4.32 (0.775)
It’s important to provide platform users with an option to learn about integrated KT (*defined as a process of involving all relevant knowledge users in the broad spectrum of research activities*)	4.38 (0.719)
It’s important to provide platform users with the option to learn about evaluating their KT tool	4.57 (0.606)
It’s important to provide platform users with the option to learn about the sustainability of their KT tool	4.45 (0.720)
It’s important to provide platform users with the option to learn about the scalability of their KT tool	4.42 (0.696)
**Overall**	
Based on my current understanding, the KaT platform would be relevant for my work	3.97 (0.793)
Based on my current understanding, I would use the KaT platform in my work	3.81 (0.835)
Overall, I like the idea of the KaT platform	4.37 (0.635)

*quality = methodological rigor and validity of the KT tool; relevance = how well the the pupose of the KT tool matches with the KT needs of the user

## Discussion

We developed a framework for creating optimized Knowledge-activated Tools (KaT) and evaluated its potential usefulness with researcher, clinician, decision-maker and trainee knowledge users. The KaT framework is evidence-informed and was developed and evaluated using rigorous methods. Of the 55 KaT framework items assessed in round 1 of our Delphi study, 16 items did not reach consensus and were re-assessed and discussed in round 2, with any remaining items being resolved in round 3. The final framework (Fig. 2) was informed by iterative changes throughout the Delphi process and represents the blueprint for what is needed to create KT strategies. This framework is not meant to be used in isolation as there is a lot of evidence and description of its domains and sub-domains supporting it (see Additional file 1: Appendices F–I). Our survey of knowledge users aimed to seek participant perceptions of the KaT framework in the context of how it may be translated into an interactive, online platform. Knowledge users liked the idea of this platform (mean score 4.36 out of 5) and its proposed features (mean score range 4.30–4.79 out of 5). We anticipate that the KaT framework will provide clarity for knowledge users about *how* to identify their KT needs and *where* these fit within the continuum of activities that can be considered for their purpose, to help *streamline* the process of developing, implementing and evaluating KT strategies to facilitate the *efficient* uptake of knowledge, and the infrastructure for an online, user-responsive platform version of KaT that will be able to generate a customized plan that *directly* responds to the needs, scope and resources of different knowledge users.

Most resources to guide KT practice have focused on one or a few aspects of creating KT strategies such as to help create a KT plan using questions, checklists or worksheets [18–22]; to outline the theory of KT, and to facilitate the uptake of knowledge [14, 18], to help with knowledge user collaboration or end-of-grant KT approaches [12, 17] and provide a searchable repository or database of KT tools [15, 16, 23]. The KaT framework is comprehensive, as it brings together all aspects of what is needed to facilitate KT practice activities in the context of creating KT strategies (i.e. processes for their development, implementation, evaluation, dissemination, sustainability and scalability and to consider Integrated KT), and it has operationalized the processes needed to ensure that rigorous and appropriate methods are applied throughout any of these activities. The KaT framework meets the need for a comprehensive and evidence-based guidance in *how* to rigorously and appropriately develop or adapt, evaluate and implement KT strategies.

Our study has some limitations. First, our systematic review (which in part informed the KaT framework) did not retrieve KT strategies beyond those that evaluated multi-chronic disease tools for older adults [28]. However, we performed a targeted literature search and consulted with our KT expert team to identify key KT resources to inform the KaT framework. Second, we may not have captured all KT experts for our Delphi panel and we were not able to assemble an internationally representative group of KT experts; they were mostly Canadian. However, our Integrated KT team does include members who are internationally known KT and Implementation Science experts. In our future work to further validate the KaT framework, we will ensure more international representation of KT experts. Third, the response rate for our KT knowledge user survey was 12%, which may limit the generalizability of our findings. However, we assembled a sampling frame of KT knowledge users using a wide search in publicly available, national and international KT networks, conferences and journals, and our goal was to achieve representativeness rather than a large sample size [28] (i.e. to include a wide range of knowledge users such as researchers, clinicians and decision-makers). Lastly, our knowledge user survey evaluated a mock-up of what an online version of KaT may look like and function, so their perceptions were limited to this representation only. However, the goals of this evaluation were to determine knowledge users’ need for such a platform and whether they would find it useful. To provide enough context for knowledge users to assess the platform’s usefulness, we focused on eliciting feedback on the most interactive aspects of the conceptual platform (i.e. *Explore* and *Action plan* domains of the KaT framework).

Our study has several strengths. First, the KaT framework is the first theory-based resource that consolidates all aspects of KT practice activities and builds on existing guidance resources by focusing on the specific steps and processes that are needed for the rigorous and efficient development of KT strategies. We have therefore advanced the science of KT by consolidating the disparate and varied sources of information into one framework. None of the currently existing KT resources operationalize the processes for creating KT strategies, and none has the infrastructure to be knowledge user-centred (i.e. the potential to deliver a customized and executable action plan that responds to their needs). This is important because context, local circumstances and needs are imperative when undertaking KT activities to ensure knowledge uptake. We therefore need to be sensitive to the variable needs of knowledge users (and their contexts) to ensure that we appropriately support their efforts across the entire spectrum of KT activities, whether their goal is to develop or adapt, implement and/or disseminate KT strategies. Second, the KaT framework will have to undergo further validation testing prior to use by knowledge users, but it represents the first step in creating an online platform that could optimize how we move research evidence into practice and policy more efficiently and rigorously with the best potential to improve health decision-making, service delivery and patient care. This is important, as we do not always transfer research evidence into practice or policy leading to wasted resources and sub-optimal impact or unsustainable implementation efforts [28]. The online platform will be user-responsive and customizable and cater to the variable needs of different audience groups (e.g. researchers vs. patients and caregivers vs. clinicians vs. policy-makers). As such, the platform will be sensitive to knowledge users who may not be familiar with concepts such as “implementation”, “theory” and “integrated KT”. The online platform will be iteratively co-designed with each type of knowledge user, and our goal will be to ensure that it will be user-friendly and relevant for each.

## Conclusions

We created and evaluated a framework for Knowledge-activated Tools (KaT) with a panel of KT experts (via a Delphi study) as well as a wide range of researcher, clinician, decision-maker and trainee knowledge users (via an online survey). Our findings suggest that KT experts perceived the KaT framework to be important and needed (88%) and they expressed interest in supporting the future development of an interactive, online version of KaT. Our survey of KT knowledge users showed that they liked the proposed features of a conceptual online KaT platform and would likely use it. As such, we anticipate that the development of this platform will bring together a wide range of stakeholders and KT experts to build an international community that could contribute to and advance the knowledge of KT as the science evolves.

## Supplementary information

**Additional file 1:** Appendices.

## Data Availability

The extracted data for the Delphi and survey studies are in the Additional file. Other data sets from this study are available upon reasonable request from the corresponding author.

## Abbreviations

KT: Knowledge translation
KaT: Knowledge-activated Tools
KTA: Knowledge-to-Action
MRC: Medical Research Council
TDF: Theoretical Domains Framework
EPOC: Effective Practice and Organisation
CIHR: Canadian Institutes of Health Research

## References

[1] Kiesler DJ, Auerbach SM (2006). Optimal matches of patient preferences for information, decision-making and interpersonal behavior: evidence, models and interventions. Patient Educ Couns..

[2] Dawes M, Sampson U (2003). Knowledge management in clinical practice: a systematic review of information seeking behavior in physicians. Int J Med Inform..

[3] McGlynn EA, Asch SM, Adams J (2003). The quality of health care delivered to adults in the United States. N Engl J Med..

[4] Chalmers I, Bracken MB, Djulbegovic B (2014). How to increase value and reduce waste when research priorities are set. Lancet..

[5] Straus S, Tetroe J, Graham I (2013). Knowledge translation in health care: moving from evidence to practice.

[6] Chalmers I, Glasziou P (2009). Avoidable waste in the production and reporting of research evidence. Lancet..

[7] Moher D, Glasziou P, Chalmers I (2016). Increasing value and reducing waste in biomedical research: who’s listening?. Lancet..

[8] Glasziou P, Chalmers I (2018). Research waste is still a scandal-an essay by Paul Glasziou and Iain Chalmers. BMJ.

[9] Covidence systematic review tool. Available at: https://www.covidence.org/home. Accessed Dec 2019.

[10] Patient-Centered Outcomes Research Institute (PCORI). Dissemination and implementation toolkit 2015. Available at: https://www.pcori.org/sites/default/files/PCORI-DI-Toolkit-February-2015.pdf. Accessed Dec 2019.

[11] Ready, Set, Change! Online decision support tool via KT Canada. Available at: http://readiness.knowledgetranslation.ca. Accessed Dec 2019.

[12] Canadian Institutes of Health Research (CIHR). Guide to knowledge translation Planning at CIHR: integrated and end-of-grant approaches. Ottawa: Canadian Institutes for Health Research; 2012. Available at: https://cihr-irsc.gc.ca/e/45321.html. Accessed Dec 2019.

[13] National Institutes of Health: Training institute for dissemination and implementation research in health (TIDIRH). Available at: https://obssr.od.nih.gov/training/training-supported-by-the-obssr/training-tidirh/. Accessed Dec 2019.

[14] Graham ID, Logan J, Harrison MB (2006). Lost in knowledge translation: time for a map?. J Contin Educ Health Prof..

[15] National Collaborating Centre for Methods and Tools (NCCMT). Registry of knowledge translation methods and tools for public health, McMaster University. Available at: https://www.nccmt.ca. Accessed Dec 2019.

[16] Health Information Research Unit: McMaster KT+ premium literature services (PLUS). Available at: https://hiru.mcmaster.ca/hiru/HIRU_McMaster_PLUS_projects.aspx. Accessed Dec 2019.

[17] Canadian Institutes of health Research (CIHR). A guide to researcher and knowledge-user collaboration in health research. Available at: https://cihr-irsc.gc.ca/e/44954.html. Accessed Dec 2019.

[18] Public Health Quebec (Institut National de Sante Publique du Quebec). Facilitating a knowledge translation process. Available at: https://www.inspq.qc.ca/pdf/publications/1628_FaciliKnowledgeTransProcess.pdf. Accessed Dec 2019.

[19] Allergen: KT planning tools for allergenic researchers. 2014. Available at: https://allergen-nce.ca/wp-content/uploads/2014/04/KTTool.pdf. Accessed Dec 2019.

[20] Melanie Barwick KT Planning template, SickKids Hospital. Available at: http://www.sickkids.ca/pdfs/Learning/58366-58366-KT_Template.pdf. Accessed Dec 2019.

[21] RE-AIM measures and checklists. checklist for study or implementation planning. Available at: http://www.re-aim.org/wp-content/uploads/2016/09/checklist_planning_intervention.pdf. Accessed Dec 2019.

[22] Institute for Work and Health: KTE resources. From research to practice: a knowledge transfer planning guide 2006. Available at: https://www.iwh.on.ca/tools-and-guides/from-research-to-practice-kte-planning-guide. Accessed Dec 2019.

[23] Gagliardi AR, Brouwers MC, Bhattacharyya OK. The development of guideline implementation tools: a qualitative study. CMAJ Open. 2015:E127–33. 10.9778/cmajo.20140064 PMC4382042 25844365

[24] Gagliardi AR, Brouwers MC, Bhattacharyya OK (2012). The guideline implementability research and application network (GIRAnet): an international collaborative to support knowledge exchange: study protocol. Implement Sci.

[25] Public Health Agency of Canada: Knowledge translation planning primer. 2012. Available at: http://publications.gc.ca/collections/collection_2013/aspc-phac/HP35-37-2012-eng.pdf. Accessed Dec 2019.

[26] Kothari A, Wathen CN (2013). A critical second look at integrated knowledge translation. Health Policy.

[27] Craig P, Dieppe P, Macintyre S, Michie S, Nazareth I, Petticrew M (2013). Developing and evaluating complex interventions: the new Medical Research Council guidance. Int J Nurs Stud..

[28] Kastner M, Cardoso R, Lai Y, Treister V, Hamid JS, Hayden L, et al. Effectiveness of interventions for managing multiple high-burden chronic diseases in older adults: a systematic review and meta-analysis. CMAJ. 2018;190(34). 10.1503/cmaj.171391 PMC6110649 30150242

[29] Cane J, O'Connor D, Michie S (2012). Validation of the theoretical domains framework for use in behaviour change and implementation research. Implement Sci..

[30] Bowen GA. Document analysis as a qualitative research method. Qual Res J. 2009;9(2).

[31] EPOC (Effective Practice and Organization of Care) taxonomy. 2015; http://epoc.cochrane.org/epoc-taxonomy. Accessed Dec, 2019.

[32] Knowledge Translation at the Canadian Institutes of Health Research (CIHR): a primer. Available at: https://ktdrr.org/ktlibrary/articles_pubs/ncddrwork/focus/focus18/Focus18.pdf. Accessed Dec 2019.

[33] Knowledge translation Canada. 2017; http://ktcanada.org/. Accessed Dec, 2019.

[34] AlbertaInnovates. Knowledge translation Platform. 2017; http://www.aihealthsolutions.ca/initiatives-partnerships/spor/knowledge-translation-platform/. Accessed Dec, 2019.

[35] Michael_Smith_Foundation. KT connects: knowledge translation webinar series. 2017; http://www.msfhr.org/ktconnects. Accessed Dec, 2019.

[36] KT Scientific Meeting: Knowledge translation Canada. 2017; http://ktcanada.org/. Accessed Dec, 2019.

[37] Annual Science of Dissemination and Implementation Conference 2017. Available at: https://www.academyhealth.org/events/site/10th-annual-conference-science-dissemination-and-implementation-health. Accessed Dec 2019.

[38] Jones J, Hunter D (1995). Consensus methods for medical and health services research. BMJ..

[39] Diamond IR, Grant RC, Feldman BM (2014). Defining consensus: a systematic review recommends methodologic criteria for reporting of Delphi studies. J Clin Epidemiol..

[40] Palinkas LA, Horwitz SM, Green CA, Wisdom JP, Duan N, Hoagwood K (2015). Purposeful sampling for qualitative data collection and analysis in mixed method implementation research. Admin Policy Mental Health Mental Health Serv Res.

[41] Canadian Association for Health Services and Policy Research (CAHSPR). Available at: https://cahspr.ca. Accessed Dec 2019.

[42] Biomed Central: Implementation Science. 2017; https://implementationscience.biomedcentral.com/. Accessed May 8, 2017.

[43] Ludwig B. Predicting the future: have you considered using the Delphi methodology? J Extens. 1997;35(5).

[44] Brouwers MC, Makarski J, Kastner M, Hayden L, Bhattacharyya O, Team G-MR (2015). The Guideline Implementability Decision Excellence Model (GUIDE-M): a mixed methods approach to create an international resource to advance the practice guideline field. Implement Sci.

[45] Rayens M, Hahn E (2000). Building consensus using the policy Delphi method. Policy Polit Nursing Prac.

[46] Dillman DA, Smyth JD (2007). Design effects in the transition to web-based surveys. Am J Prev Med..

[47] Hill K, Fowles J (1975). The methodological worth of the Delphi forecasting technique. Technol Forecast Soc Change..

[48] Hasson F, Keeney S, McKenna H (2000). Research guidelines for the Delphi survey technique. J Adv Nurs..

[49] Linstone H, Turoff M (1975). The Delphi survey: method techniques and application.

[50] Elo S, Kyngäs H (2008). The qualitative content analysis process. J Adv Nursing..

[51] Eysenbach G (2004). Improving the quality of web surveys: the Checklist for Reporting Results of Internet E-Surveys (CHERRIES). J Med Internet Res.

[52] Davis FD, Bagozzi RP, Warshaw PR (1989). User acceptance of computer technology: a comparison of two theoretical models. Manag Sci.

[53] Dalla Lana School of Public Health, University of Toronto: Centre for Quality Improvement and Patient Safety (QuIPS): staff. Available at: https://www.cquips.ca/staff/. Accessed Dec 2019.

[54] Knowledge Utilization Colloquium (2015-2018). In. Available at: https://www.ualberta.ca/nursing/research/research-units/knowledge-utilization-studies-program/knowledge-utilization-colloquia/index.html. Accessed Dec 2019.

[55] Canadian Health Policy Journal: About CHP journal. Available at: https://www.canadianhealthpolicy.com/about-chp-journal.html. Accessed Dec 2019.

[56] Journal of Health Services Research & Policy. Available at: https://journals.sagepub.com/home/hsr. Accessed Dec 2019.

[57] International Journal of Health Policy and Management: About IJHPM. Available at: www.ijhpm.com. Accessed Dec 2019.

[58] Rogers E (1995). Diffusion of innovations.

