# Supplementary material for: Improving KT tools and products: development and evaluation of a framework for creating optimized, Knowledge-activated Tools (KaT)
Source: Implement Sci Commun. 2020 May 8;1:47. doi: 10.1186/s43058-020-00031-7 (PMC7427906; doi:10.1186/s43058-020-00031-7)
Supplement: Supplementary file 1 — Additional file 1: Appendices. [file 43058_2020_31_MOESM1_ESM.docx]

**Appendix A**

Draft version of the Knowledge-activated Tools (KaT) framework


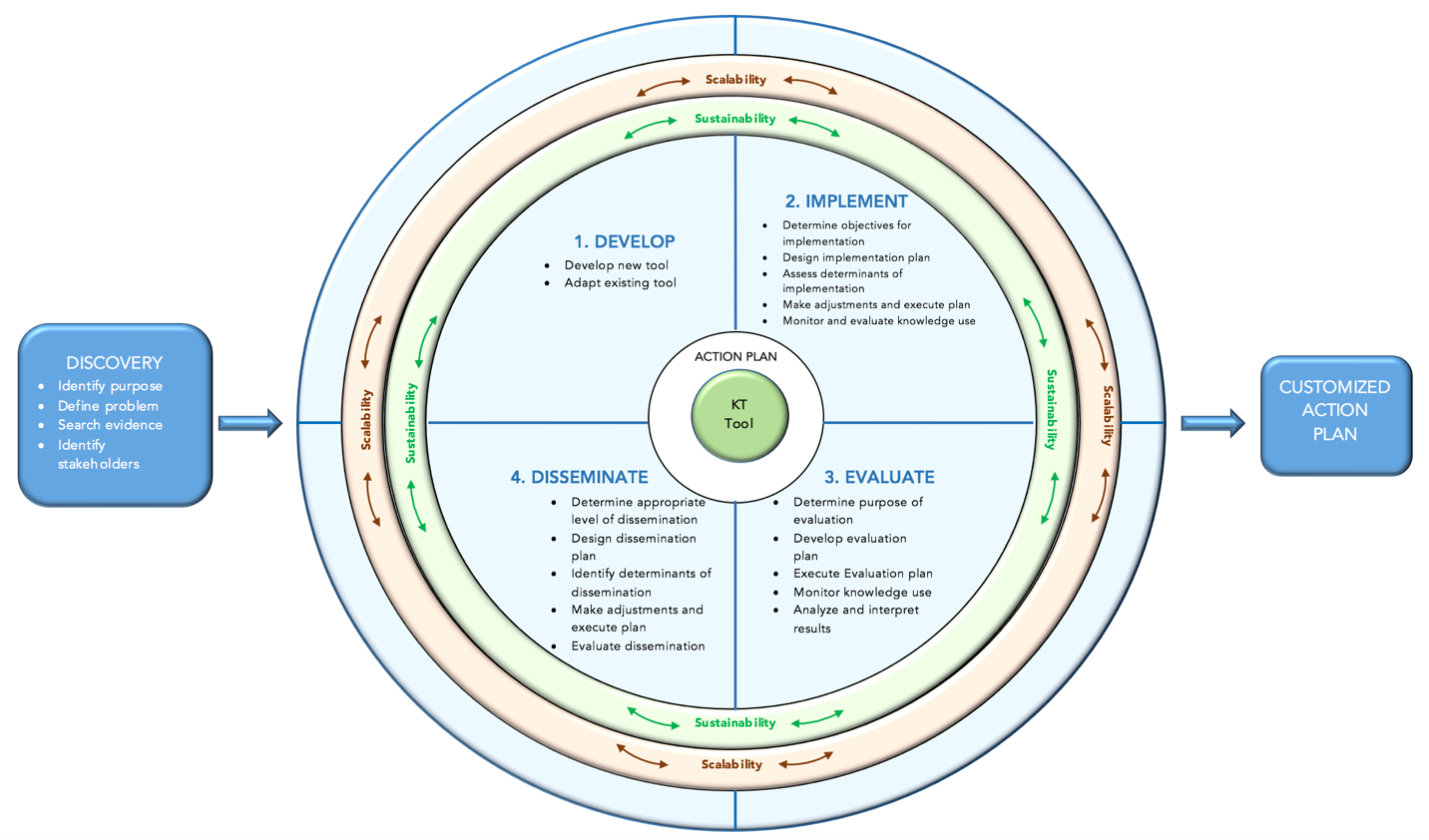


**Appendix B**

Selected questions from the Delphi Round 1 Survey

| **Survey page** | **Screen shot** |  |
| --- | --- | --- |
| **Introductory video** | 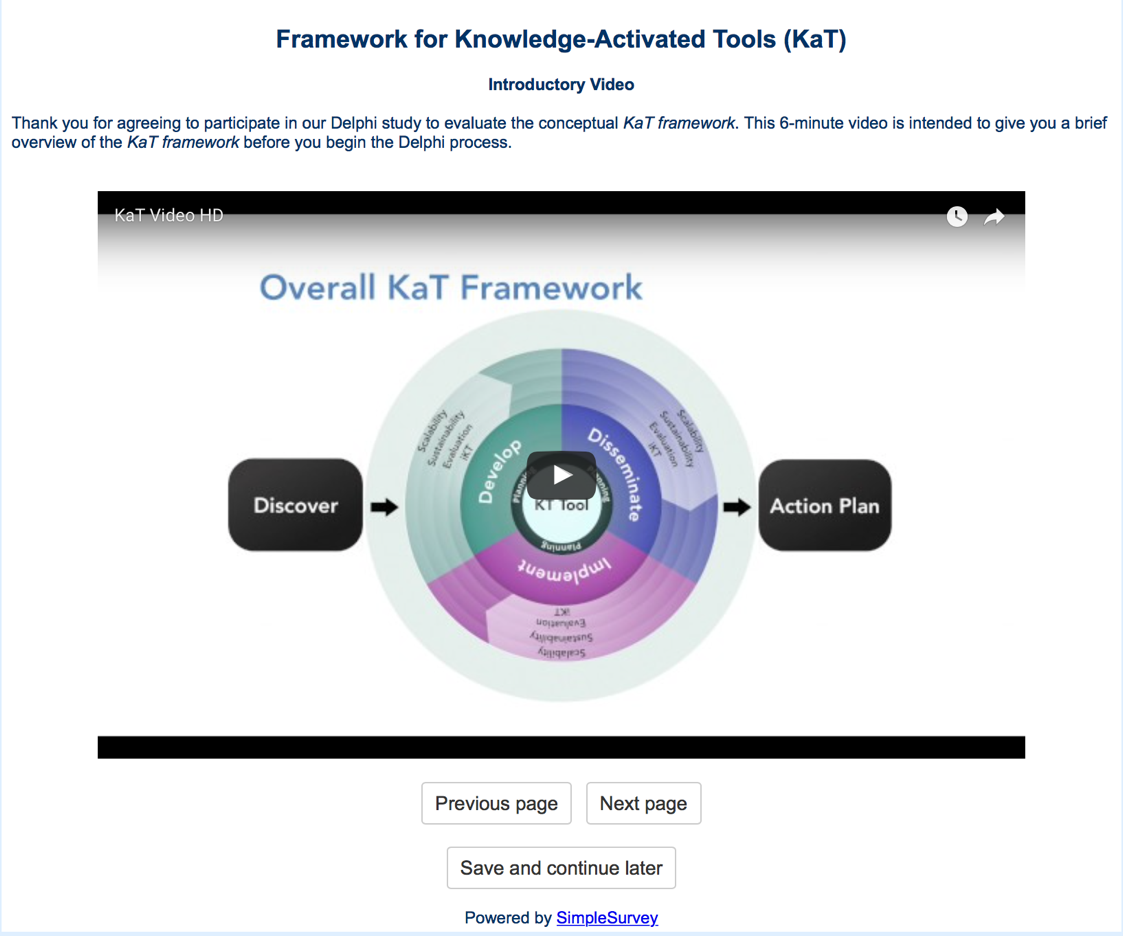 |  |
| **Survey overview** | 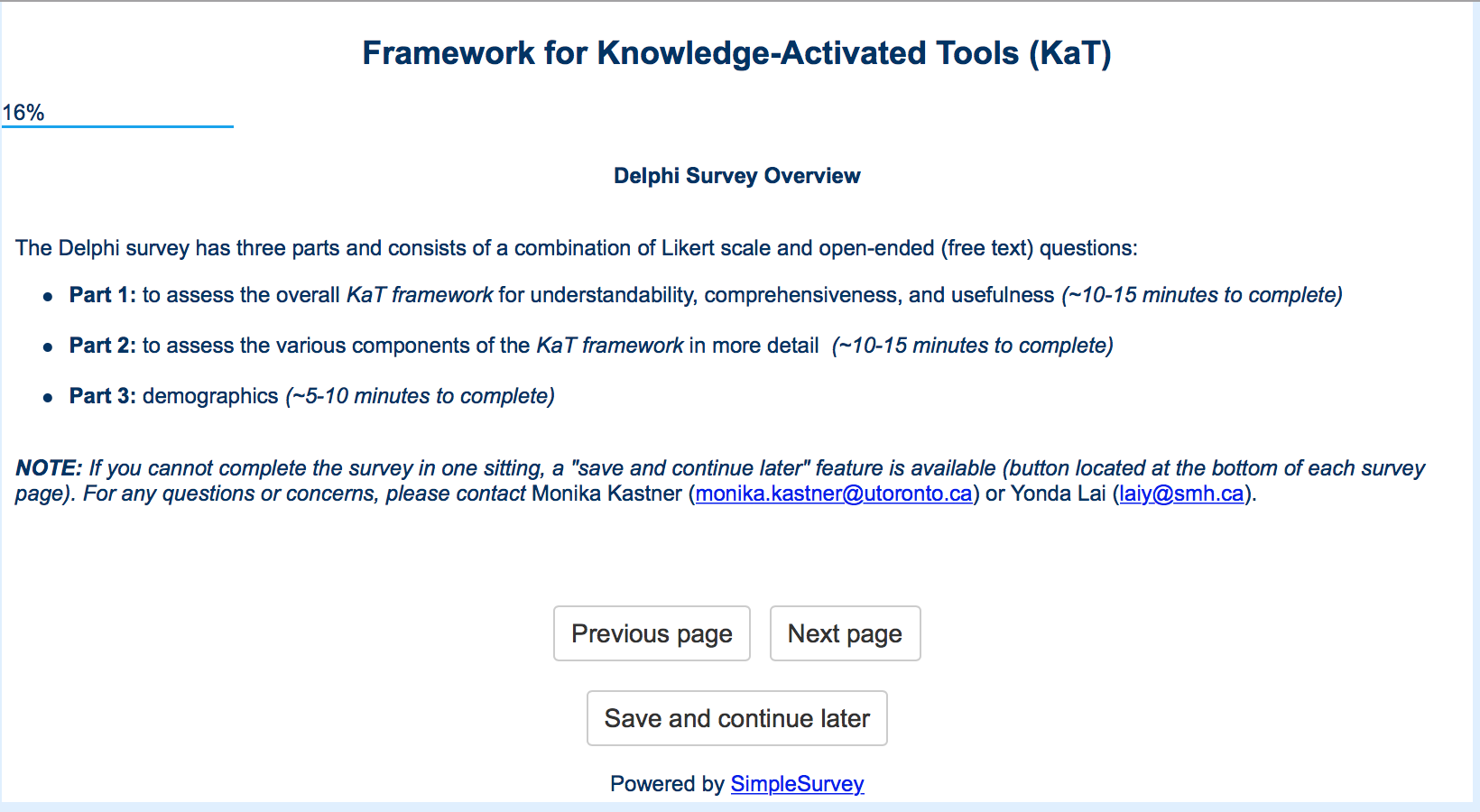 |  |
| **High-level assessment of the KaT framework:** Discover domain | 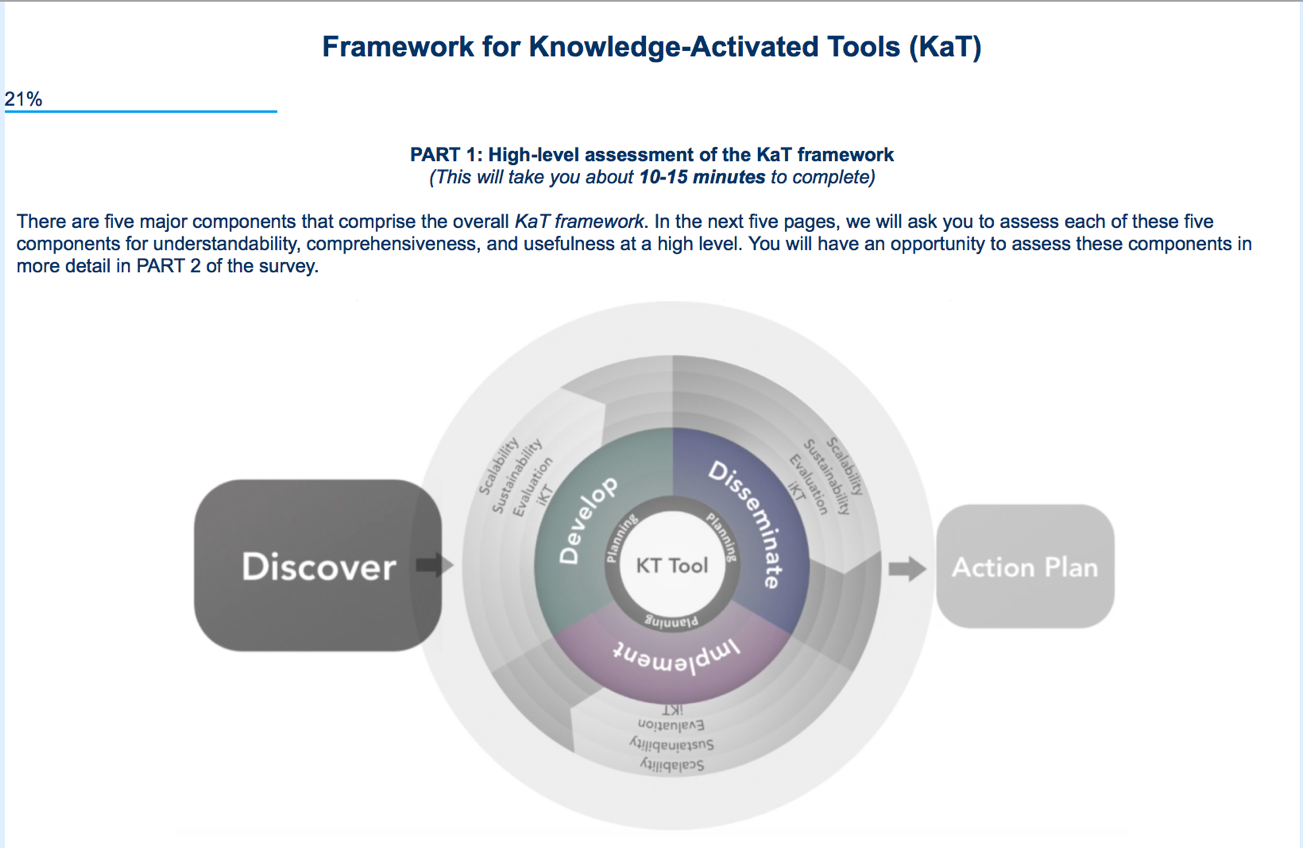 |  |
|  | 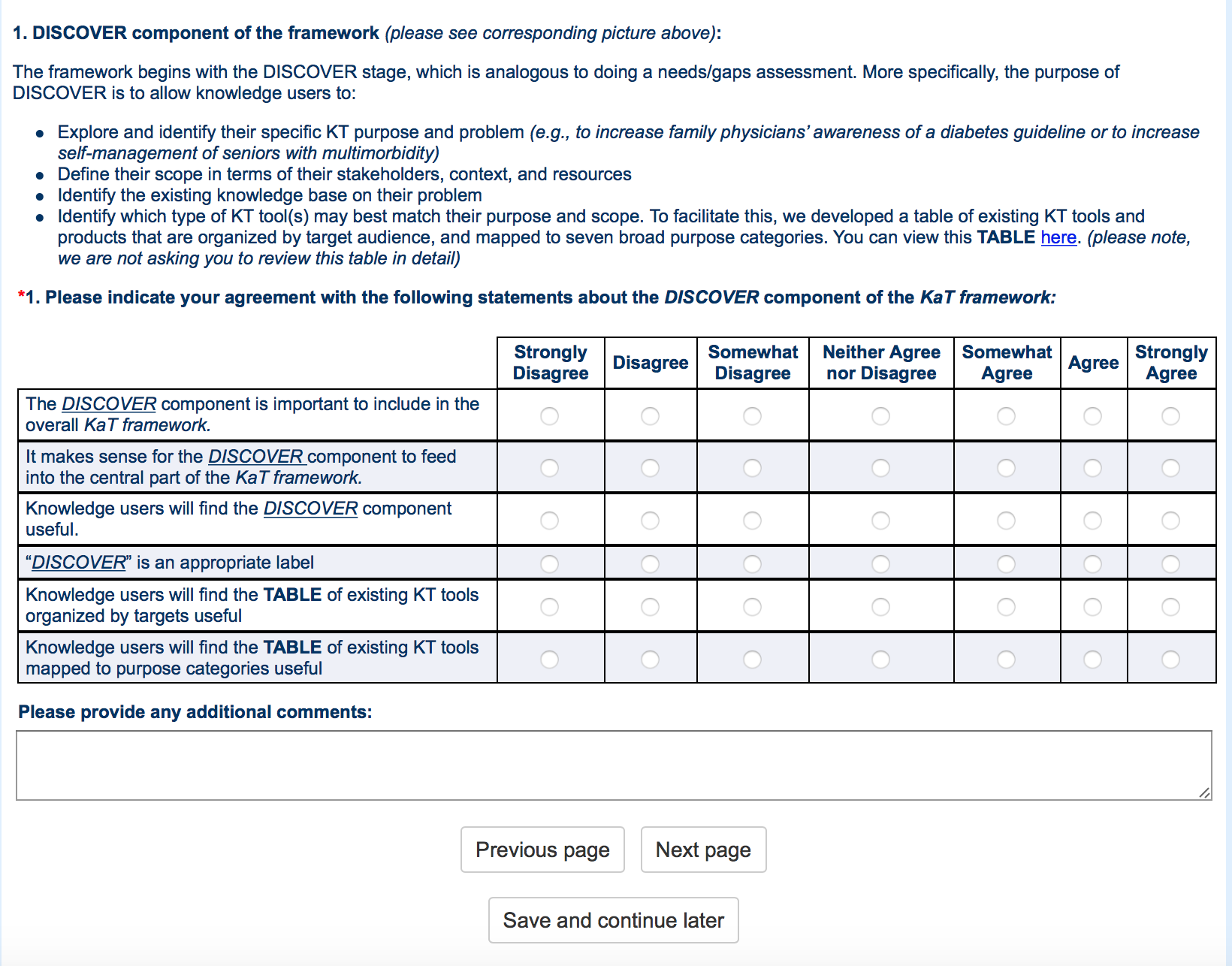 |  |
| **High-level assessment of the KaT framework:** Central component | 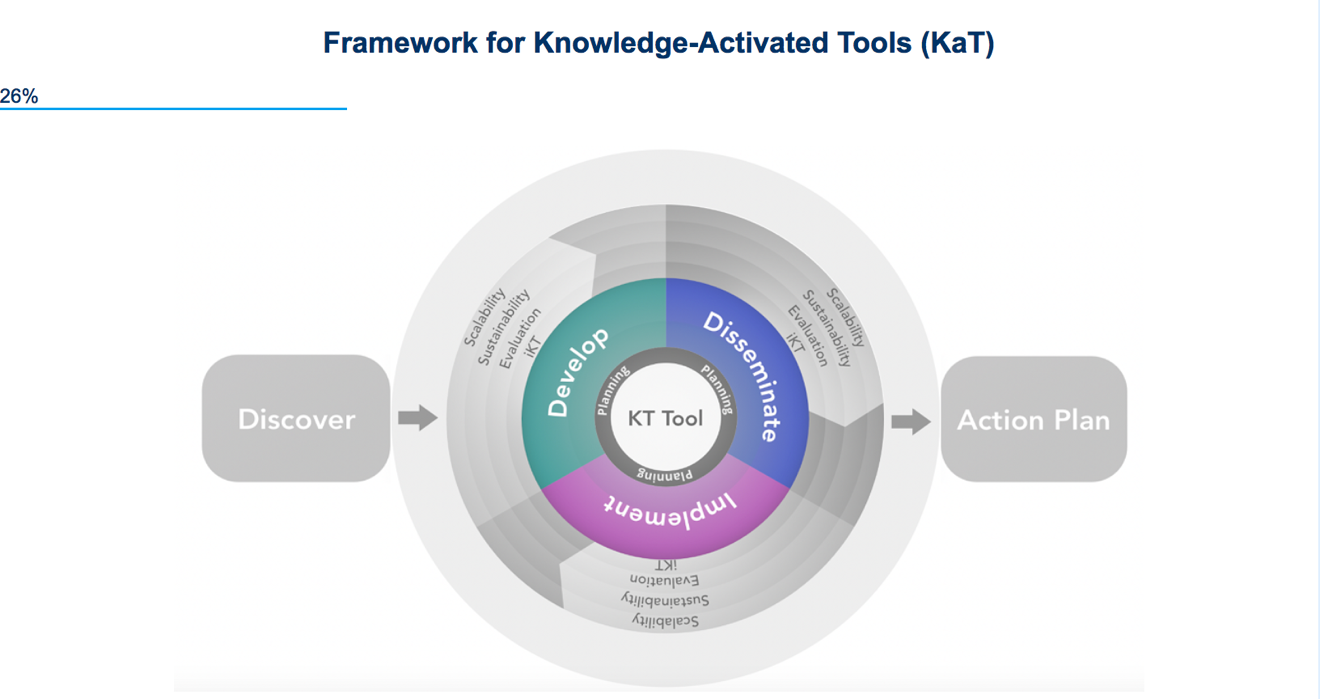 |  |
|  | 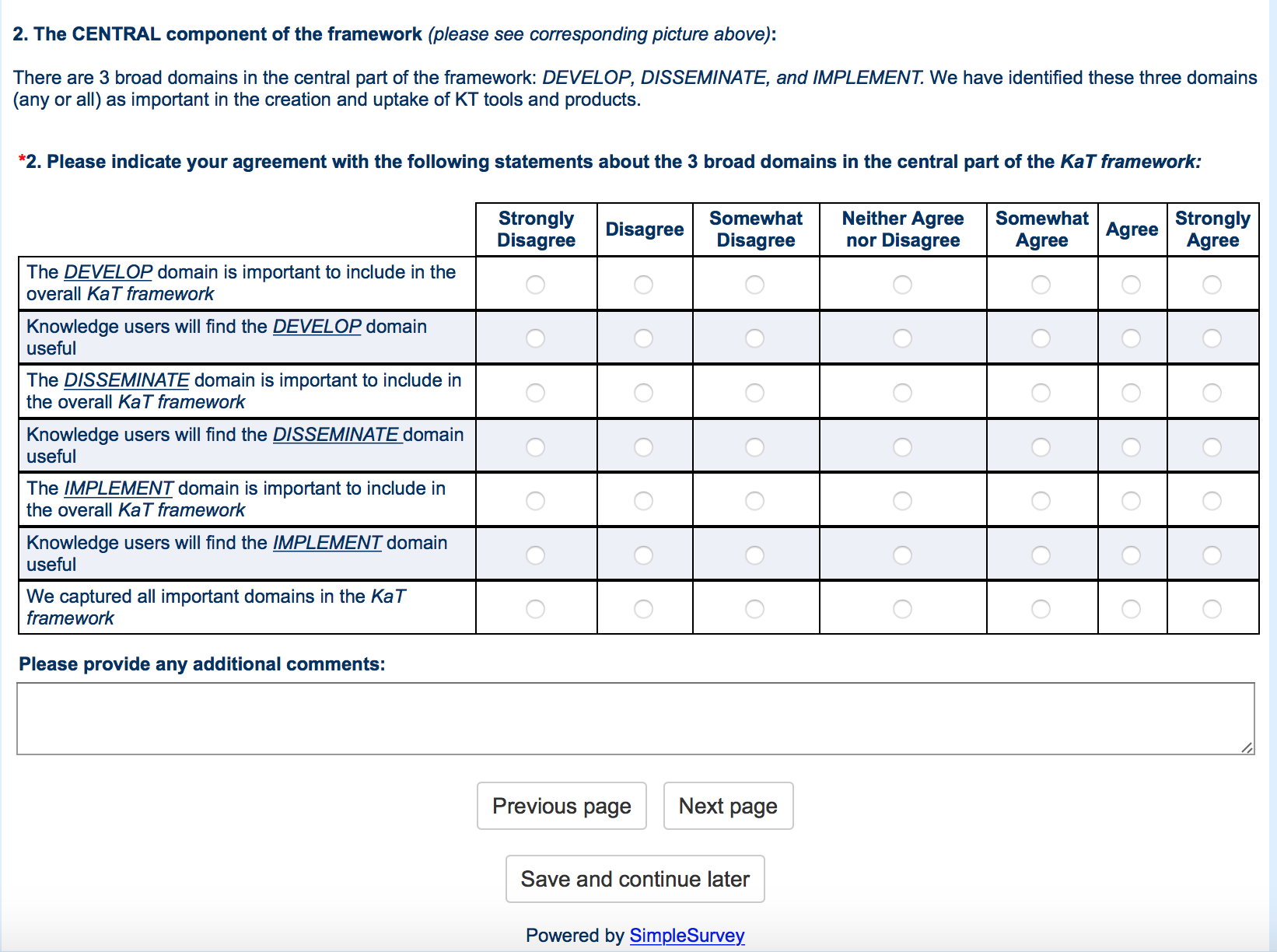 |  |
| **More detailed assessment of the KaT framework:** Discover domain and its sub-domains | 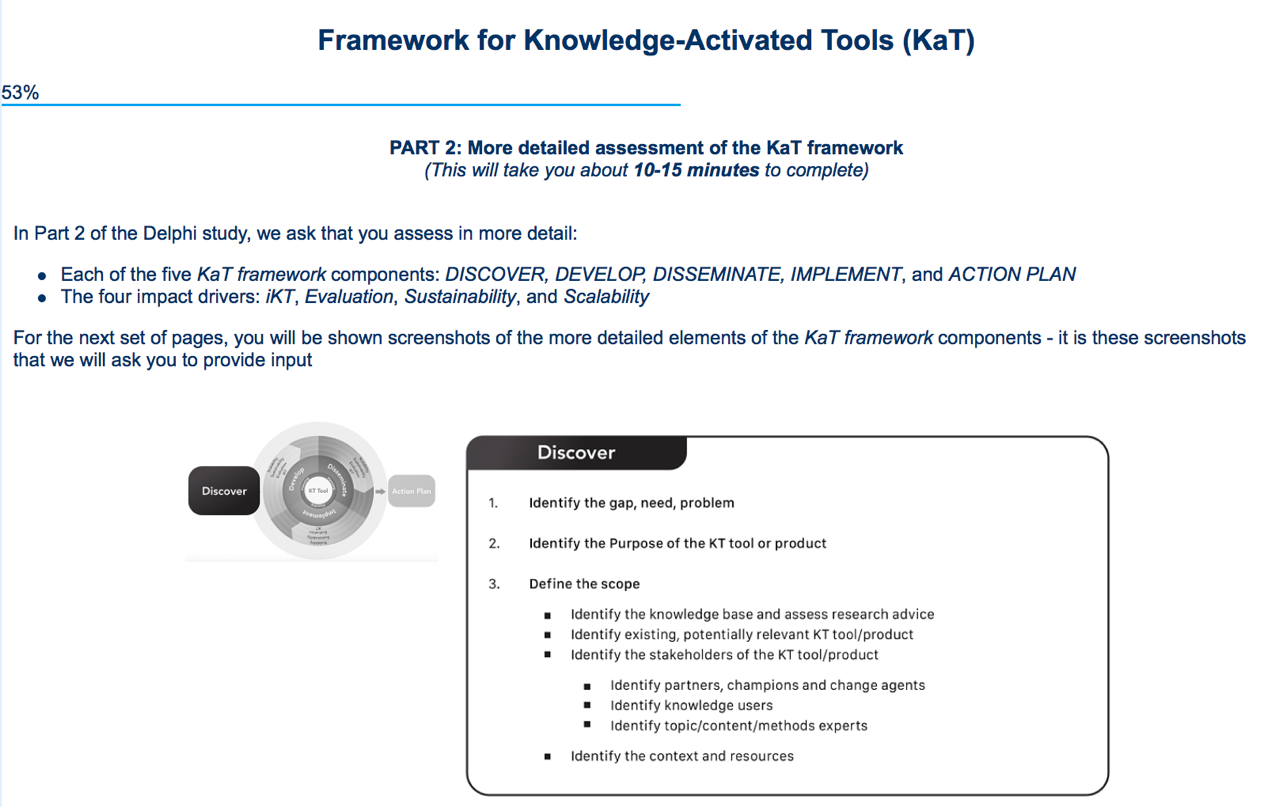 | |
|  | 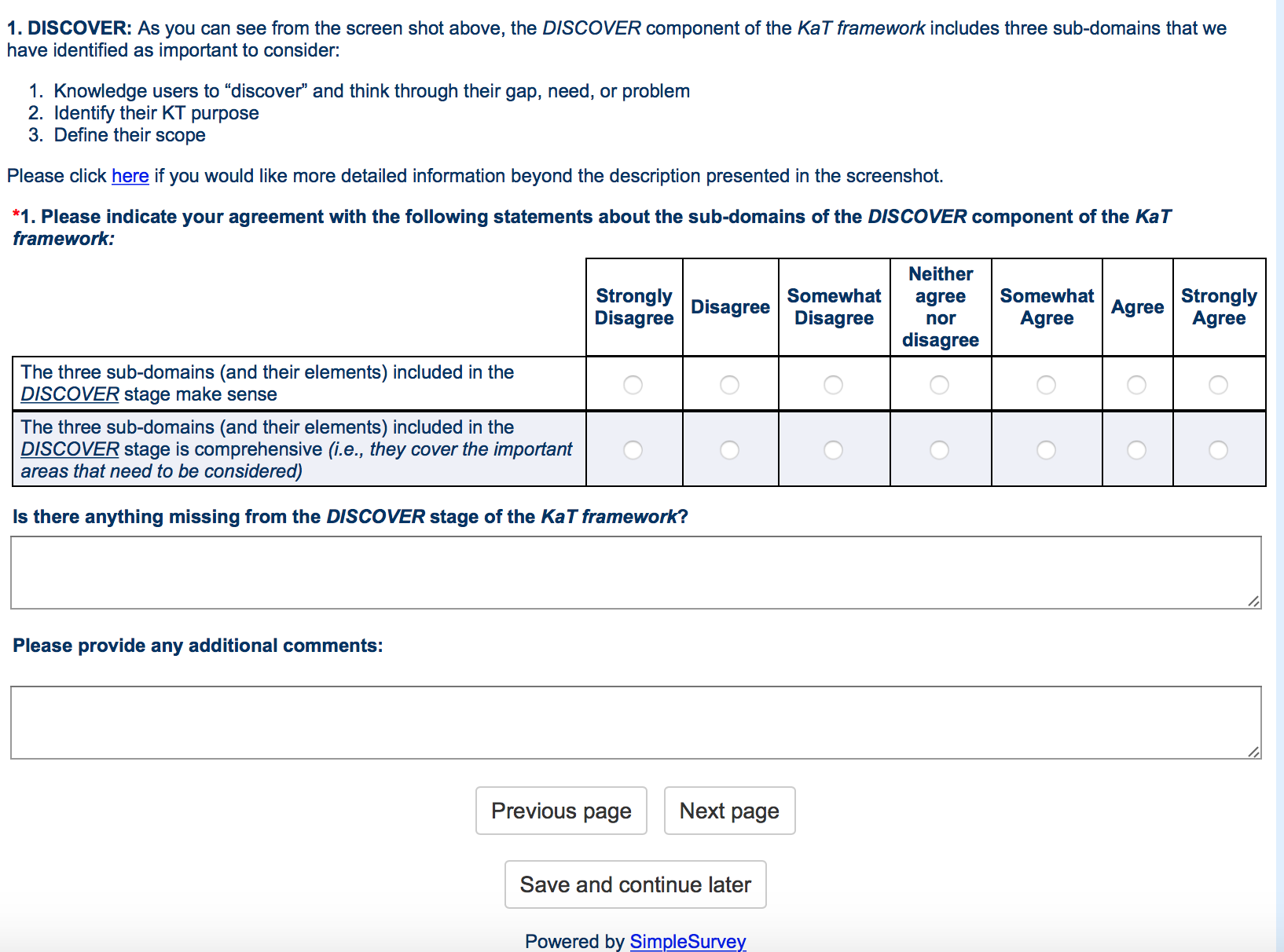 | |
| **More detailed assessment of the KaT framework:** Disseminate domain and its sub-domains | 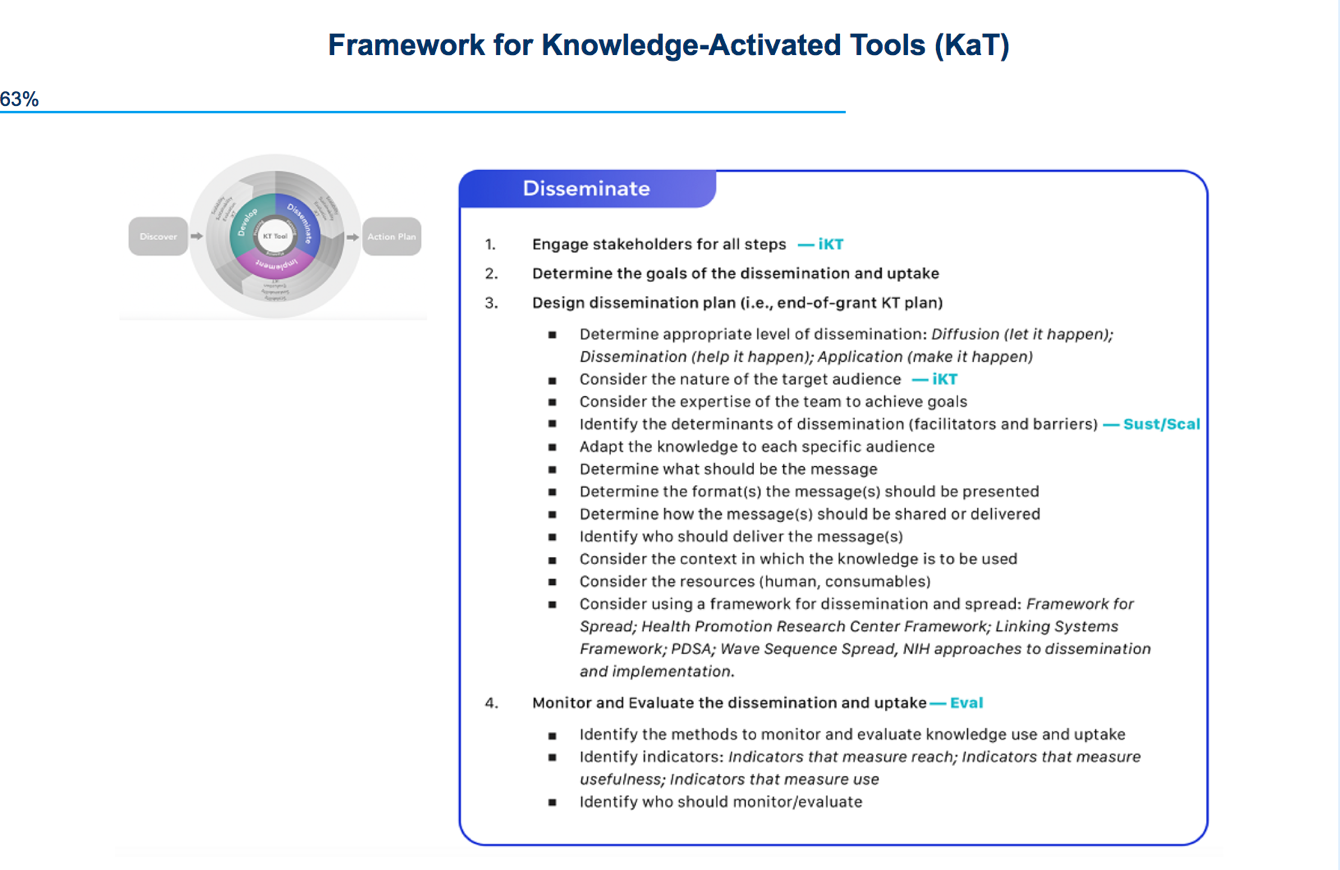 | |
|  | 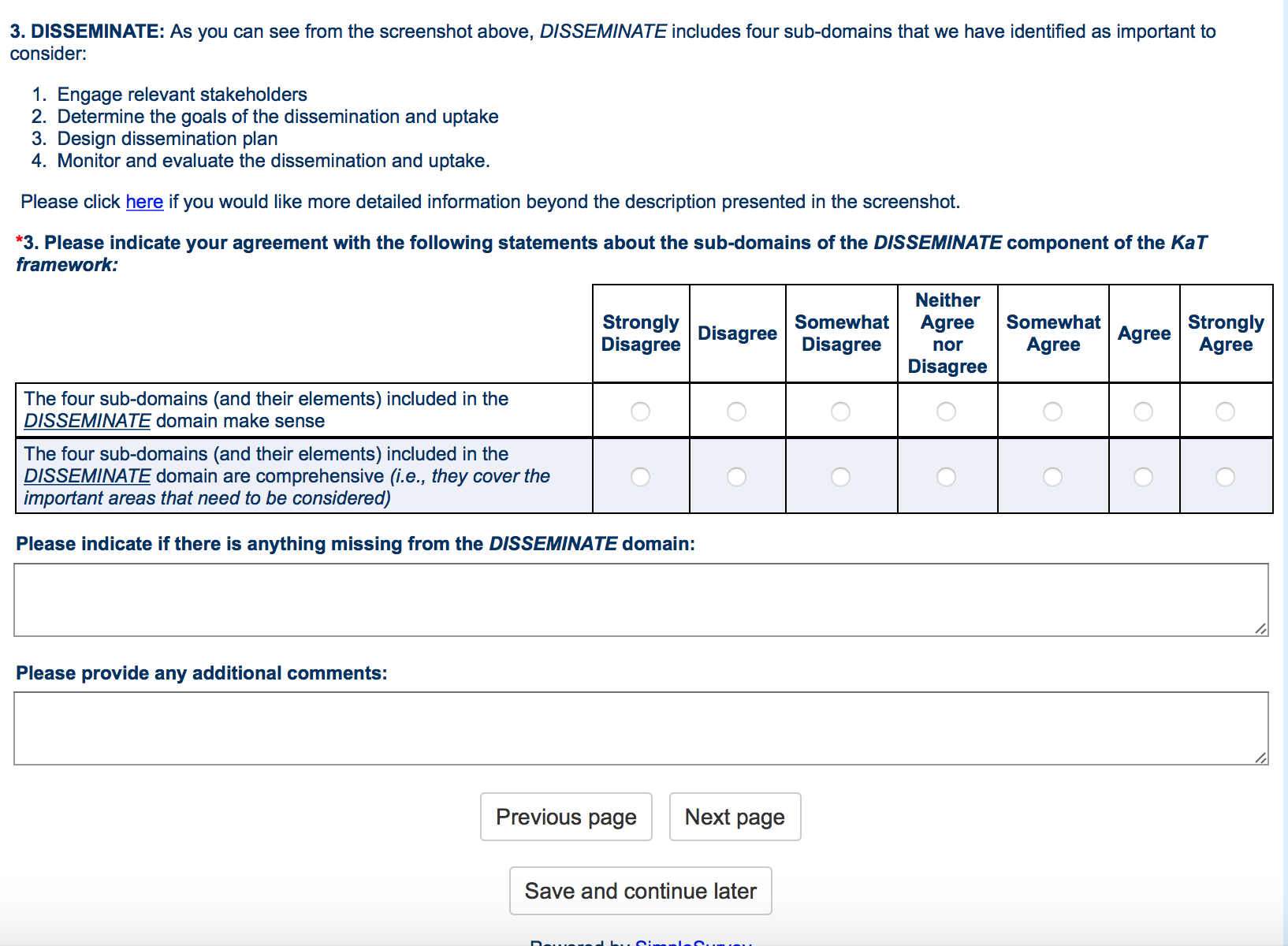 | |
| **Demographic questions** | 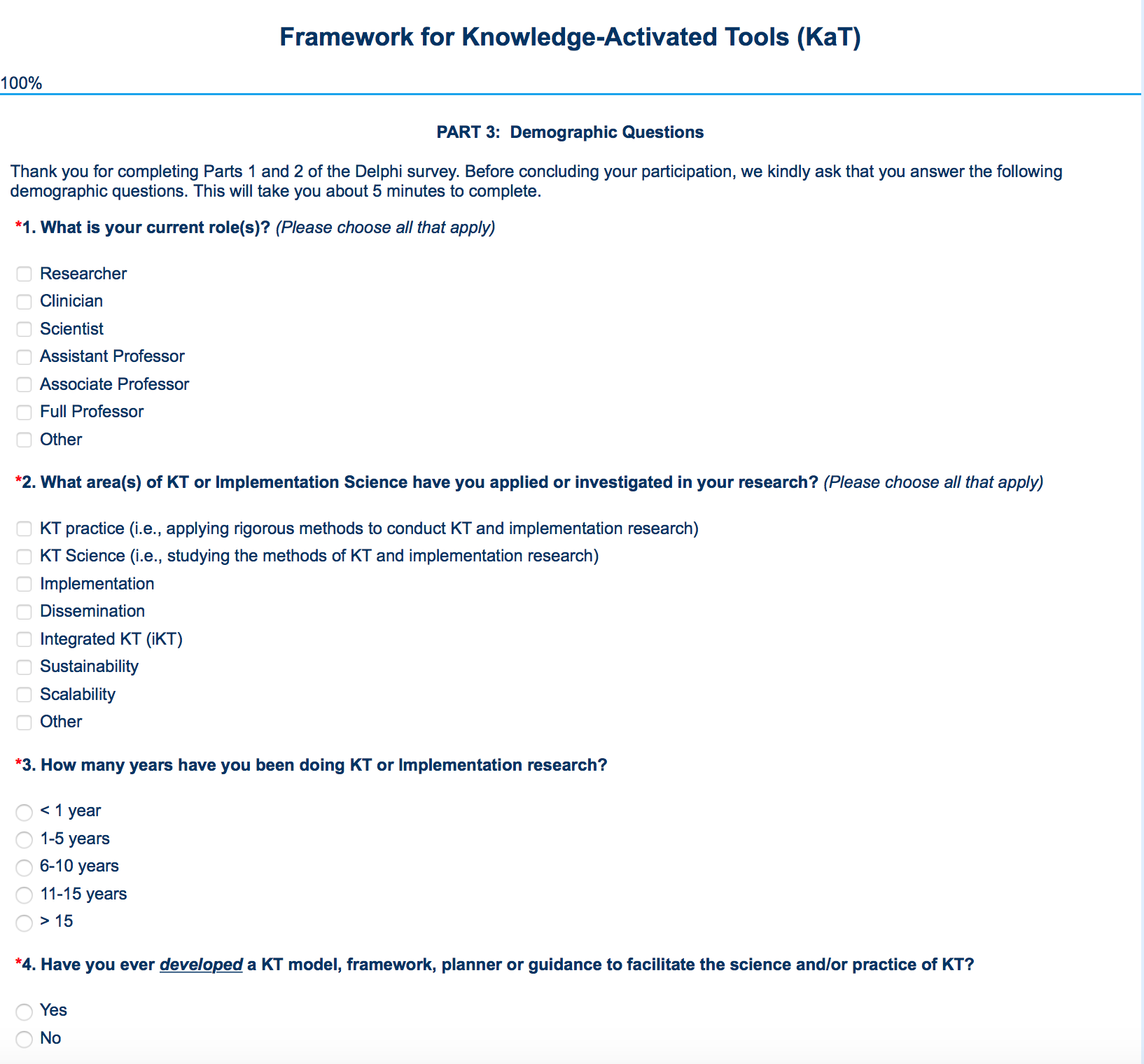 | |

**Appendix C**

| **Survey page** | **Screen shot** |
| --- | --- |
| **Perceptions of the EXPLORE web page mock-up** | 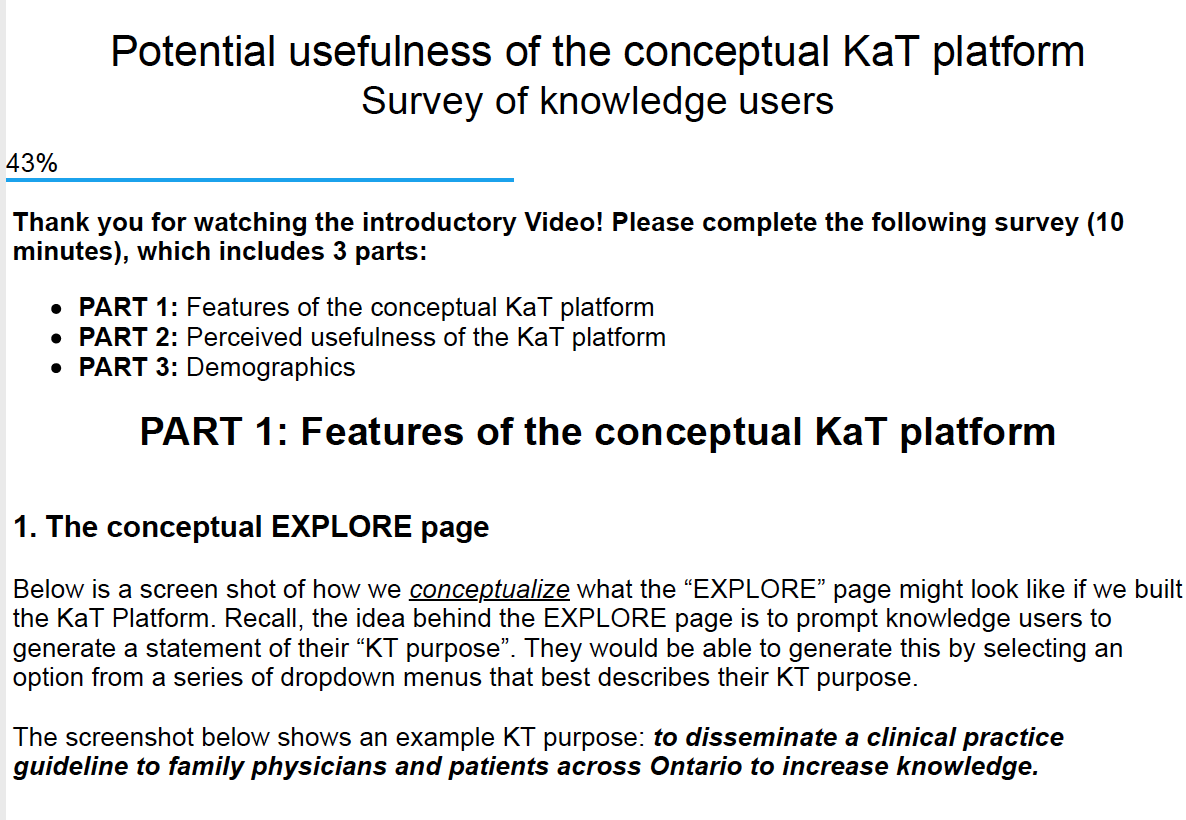 |
|  | 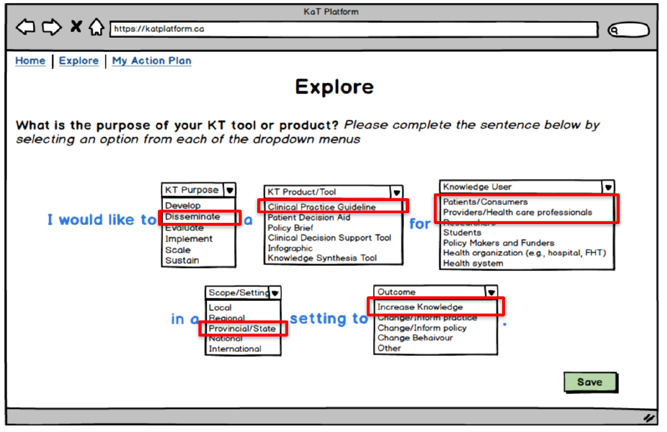 |
|  | 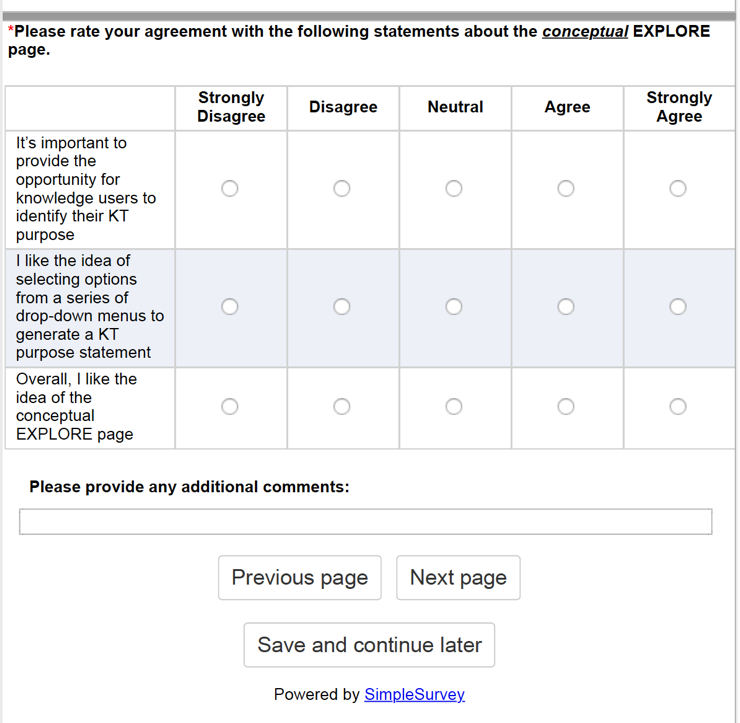 |
| **Perceptions of the ACTION PLAN web page mock-up** |  |
|  |  |
| **Demographic questions** |  |

**Appendix D**

Screen shots of the web page mock-ups of the interactive domains of the conceptual KaT platform (i.e., *Explore* and *Action Plan*)

| 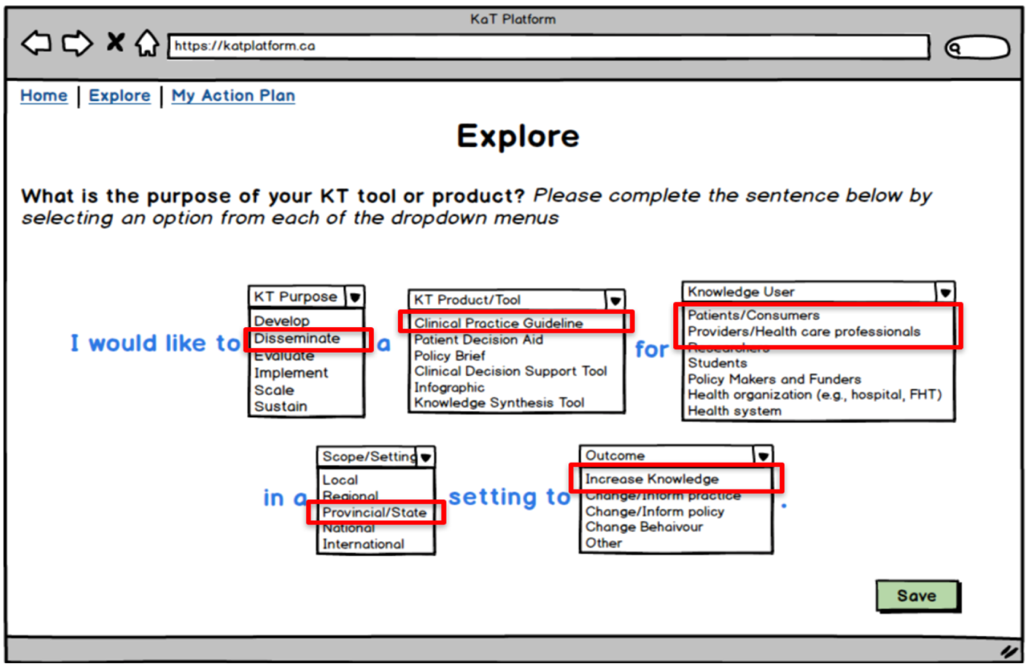 |
| --- |
| 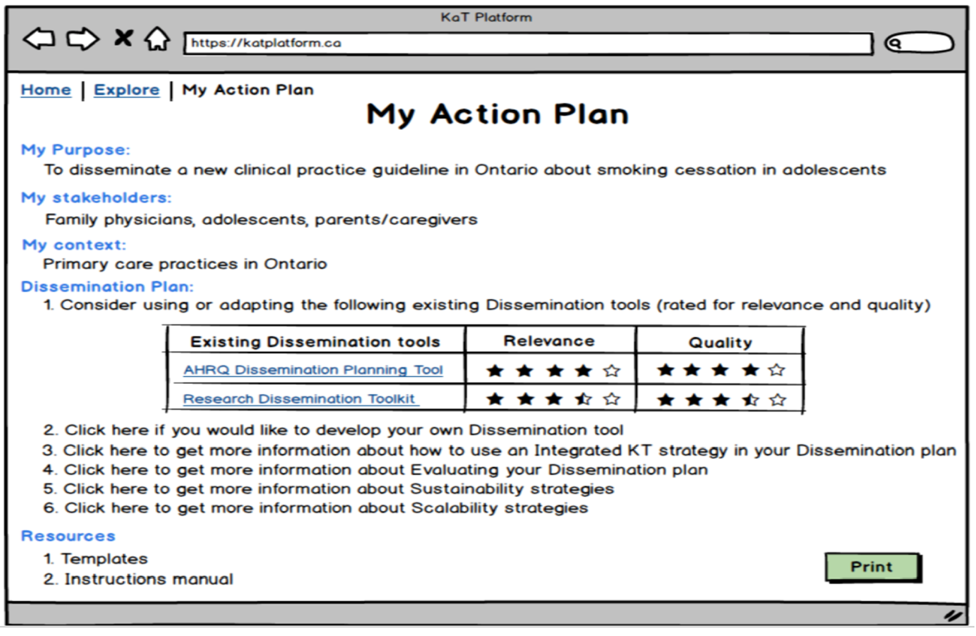 |

**Appendix E**

Existing KT strategies organized by knowledge user target audience mapped to seven broad KT purpose categories (adapted from the CIHR guidance on KT planning)

| **KT tools and products by knowledge user/target** | | **KT ACTIVITY** | | | **KT PURPOSE CATEGORY^1^** | | | | | | | | | | |
| --- | --- | --- | --- | --- | --- | --- | --- | --- | --- | --- | --- | --- | --- | --- | --- |
|  |  | **Diffusion**  Passive communication of information using delivery mechanisms for which little customization is required to reach target audiences^1^ | **Dissemination**  The communication is adapted to the specific audience and/or context in which the knowledge will be used^1^ | **Implementation**  The process of putting to use or integrating evidence into practice^1^ |  |  |  |  |  |  |  |  |  |  |  |
|  |  |  |  |  | **Increase knowledge / awareness** | **Inform future research** | **Inform / change attitudes** | | **Inform / change behaviour** | | **Inform / change policy** | **Inform / change practice** | | **Inform / change technology** | |
| **Patients/caregivers/consumers** | | | | | | | | | | | | | | |  |
|  | Alerts/reminders |  | **✓** |  |  |  |  | | | **✓** |  |  | |  | |
|  | Decision aids |  | **✓** | **✓** | **✓** |  | **✓** | | | **✓** |  |  | |  | |
|  | Educational materials |  | **✓** | **✓** | **✓** |  |  | | |  |  |  | |  | |
|  | Educational materials, printed | **✓** | **✓** |  | **✓** |  |  | | |  |  |  | |  | |
|  | Educational games |  | **✓** | **✓** | **✓** |  |  | | |  |  |  | |  | |
|  | Health information systems |  |  | **✓** | **✓** |  | **✓** | | | **✓** | **✓** | **✓** | | **✓** | |
|  | Meetings, educational |  | **✓** | **✓** | **✓** |  |  | | |  |  |  | |  | |
|  | Patient-initiated appointment systems |  | **✓** | **✓** |  |  |  | | | **✓** |  | **✓** | |  | |
|  | Self-management |  | **✓** | **✓** |  |  | **✓** | | | **✓** | **✓** |  | |  | |
|  | Social media |  | **✓** |  | **✓** |  | **✓** | | |  | **✓** |  | |  | |
|  | Summaries, plain language | **✓** | **✓** |  | **✓** |  |  | | |  |  |  | |  | |
|  | Tailored interventions |  |  | **✓** | **✓** |  | **✓** | | | **✓** | **✓** | **✓** | | **✓** | |
|  | Telemedicine and smart home technologies |  | **✓** | **✓** |  |  |  | | |  |  | **✓** | | **✓** | |
| **Providers/Healthcare professionals** | | | | | | | | | | | | | | |  |
|  | Academic conference presentations | **✓** |  |  | **✓** |  |  | | |  |  |  | |  | |
|  | Academic detailing (educational outreach) |  | **✓** | **✓** | **✓** |  |  | | | **✓** |  |  | |  | |
|  | Alerts/reminders |  | **✓** |  |  |  |  | | | **✓** |  |  | |  | |
|  | Audit and feedback |  | **✓** | **✓** |  |  |  | | | **✓** |  |  | |  | |
|  | Care pathways |  | **✓** | **✓** |  |  |  | | | **✓** |  |  | |  | |
|  | Case management |  | **✓** | **✓** |  |  |  | | | **✓** |  |  | |  | |
|  | Computerized decision support aids/systems |  |  | **✓** |  |  |  | | |  |  | **✓** | | **✓** | |
|  | Clinical practice guidelines |  | **✓** |  | **✓** |  |  | | | **✓** |  |  | |  | |
|  | Communication between providers | **✓** |  |  | **✓** |  | **✓** | | |  |  |  | |  | |
|  | Comprehensive geriatric assessment |  |  | **✓** |  |  |  | | |  |  | **✓** | |  | |
|  | Continuity of care |  | **✓** |  |  |  |  | | |  |  | **✓** | |  | |
|  | Continuous quality improvement |  |  |  |  |  |  | | |  |  | **✓** | |  | |
|  | Discharge planning |  | **✓** | **✓** |  |  |  | | |  |  | **✓** | |  | |
|  | Disease management |  |  |  |  |  |  | | | **✓** |  | **✓** | |  | |
|  | Education materials, printed | **✓** |  |  | **✓** |  |  | | |  |  |  | |  | |
|  | Educational meetings |  | **✓** |  | **✓** |  |  | | |  |  |  | |  | |
|  | Engage champions / opinion leaders |  | **✓** |  |  |  | **✓** | | |  |  |  | |  | |
|  | Financial incentives |  | **✓** |  |  |  |  | | | **✓** |  |  | |  | |
|  | Health information systems |  |  | **✓** |  |  |  | | |  |  | **✓** | | **✓** | |
|  | Inter-professional education |  | **✓** | **✓** | **✓** |  |  | | |  |  |  | |  | |
|  | Knowledge brokering |  | **✓** |  | **✓** |  | **✓** | | |  | **✓** |  | |  | |
|  | Local consensus processes |  |  | **✓** |  |  | **✓** | | |  |  |  | |  | |
|  | Local opinion leaders |  |  | **✓** |  |  | **✓** | | |  |  |  | |  | |
|  | Media release /outreach campaign/ interviews |  | **✓** |  |  |  | **✓** | | |  |  |  | |  | |
|  | Meetings, educational |  | **✓** |  | **✓** |  |  | | |  |  |  | |  | |
|  | Meetings, face-to-face |  | **✓** |  | **✓** |  | **✓** | | |  |  |  | |  | |
|  | Meetings, Interactive small group meeting or workshop |  | **✓** |  | **✓** |  | **✓** | | |  |  |  | |  | |
|  | Meetings with stakeholders |  | **✓** |  | **✓** |  | **✓** | | |  |  |  | |  | |
|  | Patient-mediated interventions |  | **✓** | **✓** | **✓** |  |  | | |  |  |  | |  | |
|  | Publications, non-peer reviewed | **✓** |  |  | **✓** |  |  | | |  |  |  | |  | |
|  | Publications, peer-reviewed (academic journals) | **✓** |  |  | **✓** |  |  | | |  |  |  | |  | |
|  | Referral systems |  |  | **✓** |  |  |  | | |  |  | **✓** | |  | |
|  | Seminars and workshops | **✓** |  |  | **✓** |  | **✓** | | |  |  |  | |  | |
|  | Shared care |  |  | **✓** |  |  |  | | |  |  | **✓** | |  | |
|  | Social media |  | **✓** |  | **✓** |  |  | | |  |  |  | |  | |
|  | Summaries | **✓** |  |  | **✓** |  |  | | |  |  |  | |  | |
|  | Summary briefings to stakeholders |  | **✓** |  | **✓** |  |  | | |  |  |  | |  | |
|  | Tailored interventions |  |  | **✓** | **✓** |  | **✓** | | | **✓** |  |  | |  | |
|  | Teams |  |  | **✓** |  |  |  | | |  |  | **✓** | |  | |
|  | Transitions of care |  |  | **✓** |  |  |  | | |  |  | **✓** | |  | |
|  | Web-based activities | **✓** |  |  | **✓** |  |  | | |  |  |  | |  | |
| **Mixed: Patients + Providers** | | | | | | | | | | | | | | |  |
|  | Shared decision-making |  |  | **✓** | **✓** |  | **✓** | | | **✓** | **✓** | |  |  | |
|  | Telemedicine / smart home tech |  | **✓** | **✓** | **✓** |  |  | | |  |  | | **✓** | **✓** | |
| **Researchers & Students** | | | | | | | | | | | | | | |  |
|  | Academic conference presentations | **✓** |  |  | **✓** | **✓** |  | | |  |  | |  |  | |
|  | Alerts/Reminders |  | **✓** |  |  |  |  | | | **✓** |  | |  |  | |
|  | Computerized decision support aids/systems |  | **✓** | **✓** |  |  |  | | |  |  | | **✓** | **✓** | |
|  | Engage champions / opinion leaders |  | **✓** |  |  |  | **✓** | | |  |  | |  |  | |
|  | Health information systems |  |  | **✓** | **✓** |  |  | | |  |  | | **✓** | **✓** | |
|  | Inter-professional education | **✓** |  |  | **✓** | **✓** | **✓** | | |  |  | |  |  | |
|  | Knowledge brokering |  | **✓** |  | **✓** | **✓** | **✓** | | |  |  | |  |  | |
|  | Local consensus processes |  | **✓** |  |  |  | **✓** | | |  |  | |  |  | |
|  | Local opinion leader |  | **✓** |  |  |  | **✓** | | |  |  | |  |  | |
|  | Media release/ outreach campaign/ interviews | **✓** | **✓** |  | **✓** |  | **✓** | | |  |  | |  |  | |
|  | Meetings, educational |  | **✓** |  | **✓** |  |  | | |  |  | |  |  | |
|  | Publications, non-peer reviewed | **✓** |  |  |  |  |  | | |  |  | |  |  | |
|  | Publications, peer-reviewed (academic journals) | **✓** |  |  | **✓** | **✓** |  | | |  |  | |  |  | |
|  | Social media |  | **✓** |  | **✓** |  | **✓** | | |  |  | |  |  | |
|  | Summaries | **✓** |  |  | **✓** |  |  | | |  |  | |  |  | |
|  | Web-based activities | **✓** |  |  | **✓** |  |  | | |  |  | |  |  | |
| **Policy makers and Funders** | | | | | | | | | | | | | | |  |
|  | Alerts/reminders |  | **✓** |  |  |  | |  | | **✓** |  | |  |  | |
|  | Continuous quality improvement |  |  | **✓** |  |  | |  | |  |  | | **✓** |  | |
|  | Engage champions / opinion leaders |  | **✓** |  |  |  | | **✓** | |  | **✓** | |  |  | |
|  | Inter-professional education |  | **✓** |  | **✓** |  | | **✓** | |  | **✓** | |  |  | |
|  | Local consensus processes |  | **✓** |  | **✓** |  | | **✓** | |  | **✓** | |  |  | |
|  | Local opinion leaders |  | **✓** |  |  |  | | **✓** | |  | **✓** | |  |  | |
|  | Media release /outreach campaign/ interviews |  | **✓** |  |  |  | | **✓** | |  |  | |  |  | |
|  | Meetings, interactive small group meeting/workshop |  | **✓** |  |  |  | | **✓** | |  |  | |  |  | |
|  | Policy briefs |  | **✓** |  | **✓** |  | | **✓** | |  | **✓** | |  |  | |
|  | Social media |  | **✓** |  | **✓** |  | | **✓** | |  | **✓** | |  |  | |
|  | Summary briefings to stakeholders |  | **✓** |  | **✓** |  | |  | |  | **✓** | |  |  | |
|  | Web-based activities | **✓** |  |  | **✓** |  | |  | |  |  | |  |  | |
| **Health care organizations (e.g., hospitals, family health teams)** | | | | | | | | | | | | | | |  |
|  | Alerts/reminders |  | ✓ |  |  |  | |  | | **✓** |  | |  |  | |
|  | Audit and feedback (performance feedback) |  | **✓** | ✓ |  |  | |  | | **✓** |  | |  |  | |
|  | Communities of practice |  | **✓** |  |  |  | | **✓** | |  |  | | **✓** |  | |
|  | Continuous quality improvement |  |  | ✓ |  |  | |  | |  |  | | **✓** |  | |
|  | Engage champions / opinion leaders |  | **✓** |  |  |  | | **✓** | |  |  | |  |  | |
|  | Health information systems |  |  | **✓** |  |  | |  | |  |  | | **✓** | **✓** | |
|  | Inter-professional education |  | **✓** |  | **✓** |  | | **✓** | |  |  | |  |  | |
|  | Local consensus processes |  | **✓** |  |  |  | | **✓** | |  |  | |  |  | |
|  | Local opinion leaders |  | **✓** |  |  |  | | **✓** | |  | **✓** | |  |  | |
|  | Media release /outreach campaign / interviews |  | **✓** |  |  |  | | **✓** | |  |  | |  |  | |
|  | Meetings, interactive small group meeting/workshop |  | **✓** |  | **✓** |  | | **✓** | |  |  | |  |  | |
|  | Monitoring the performance of the delivery of healthcare |  |  | **✓** |  |  | |  | | **✓** | **✓** | | **✓** |  | |
|  | Summary briefings to stakeholders |  | **✓** |  | **✓** |  | |  | |  |  | |  |  | |
|  | Telemedicine / smart home tech |  |  | **✓** |  |  | |  | |  |  | | **✓** | **✓** | |
|  | Web-based activities | **✓** |  |  | **✓** |  | |  | |  |  | |  |  | |
| **Health system** | | | | | | | | | | | | | | |  |
|  | Communities of practice |  | **✓** |  |  |  |  | | |  |  | | **✓** |  | |
|  | Engage champions / opinion leaders |  | **✓** |  |  |  |  | | |  |  | |  |  | |
|  | Health information systems |  |  | **✓** |  |  |  | | |  | **✓** | |  |  | |
|  | Media release / outreach campaign / interviews |  | **✓** |  |  |  | **✓** | | |  | **✓** | |  |  | |
|  | Monitoring the performance of the delivery of healthcare |  |  | **✓** |  |  |  | | | **✓** | **✓** | | **✓** |  | |
|  | Web-based activities | **✓** |  |  | **✓** |  |  | | |  |  | |  |  | |

^1^Adapted from the CIHR guidance on KT planning (Available at: <http://www.cihr-irsc.gc.ca/e/45321.html>.)

**Appendix F**

Details of the DISCOVER (later called EXPLORE) domain and its sub-domains of the framework for Knowledge-Activated Tools (KaT)

| **EXPLORE:** The KaT framework begins with the discovery stage, which allows knowledge users to “discover” and think through their gap, need, or problem; identify their KT purpose; and to define their scope. A set of outputs are generated from this discovery to inform a recommended pathway of next steps. | | | |
| --- | --- | --- | --- |
| **Sub-domain** | | | **Purpose/Description** |
| 1. **Identify the gap, need, problem** | | | To identify the gap, need, problem. Consider the type of changes that need to happen to address the problem, and what would be different if the problem is addressed (outcomes). A needs assessments process can be employed to determine the size and nature of the gap between current and more desirable knowledge, skills, attitudes, behaviours, and outcomes^1-3^ |
| 1. **Identify the purpose of the KT tool or product** | | | Identifying the KT purpose will help refine the problem and to inform the most appropriate KT tool/product that may be considered to develop or adapt to address the purpose. Knowledge users will be encouraged to think about how this inquiry will add to what is already known, how successful translation of knowledge might be measured, and what changes they would like to see happen. Purpose categories include: To increase, generate, share knowledge and awareness; to inform or change attitudes, behaviour, practice, policy, or technology^1^ |
| 1. **Define the scope** | | | Use identified purpose to identify the scope of the KT activity by identifying the knowledge base of the problem and existing KT tools or products; identify relevant stakeholders and knowledge users; and to define the context in which the KT tool or product will be used |
|  | 1. **Identify the knowledge base and assess research evidence** | | Search the evidence for existing knowledge to determine if the problem or purpose has been investigated or addressed; and assess the quality of the evidence. Identifying the knowledge base can be viewed from a scientific (e.g., systematic review to inform new clinical practice, a qualitative study to inform policy change), experiential (e.g., a therapeutic practice endorsed by patients based on their own experiences), or pragmatic (a practice pattern that seems to work stemming from a day-to-day clinical problem) perspective. If there is a need for a knowledge synthesis, select the method that best matches the research question |
|  | 1. **Identify existing, potentially relevant KT tools/products** | | Use our tools table (KT purpose categories mapped to a wide range of KT tools/products organized by different knowledge end-users) to identify potentially relevant tools/products that may be considered to address identified problem/purpose |
|  | 1. **Identify the stakeholders of the KT tool or product** | | Identify the targets, key stakeholders, knowledge users and experts; based on the KT purpose. Think about who will need to be involved? How will they influence the project? |
|  |  | **Identify partners and champions and change agents** | Individuals who will partner on the research; individuals who will champion implementation of the KT tool/product (e.g., local leaders, peer/organizational champions or other agents of change who will motivate knowledge users to adopt new actions |
|  |  | **Identify knowledge users** | Individuals or communities who will be interested in the knowledge, and will use or apply the knowledge that will be generated: e.g., providers, patients, caregivers, researchers, policy/decision makers, family health team, communities of practice |
|  |  | **Identify topic/content/methods experts** | Identify experts that will be needed based on identified objectives and purpose such as in the relevant content area, biostatistics, health economic analysis, behavior theory/change, KT, etc. |
|  | 1. **Identify the context and resources** | | Identify the context in which the KT tool will be implemented and used. In which setting will the tool be implemented and used? (i.e., consider sociodemographics, geography, language, and culture); How will the tool be delivered? Who will deliver? What resources are available (human, financial, consumables) and what technology and other resources are needed for intervention delivery (e.g., Internet connection, media skills, design)? What is feasible? |

^1^Canadian Institutes of Health Research (CIHR): Guide to Knowledge Translation Planning at CIHR: Integrated and End-of-Grant Approaches; 2012. Available at: <http://www.cihr-irsc.gc.ca/e/45321.html>.

^2^Kitson A and Straus SE. The knowledge-to-action cycle: identifying the gaps. CMAJ 2009.

^3^Straus SE, Tetroe J, Graham ID. Knowledge translation in health care: Moving from evidence to practice. 2nd ed. West Sussex, UK: Wiley; 2013

**Appendix G**

Details of the central domains and their sub-domains of the framework for Knowledge-Activated Tools (KaT): DEVELOP OR ADAPT, DISSEMINATE, IMPLEMENT

| **DEVELOP OR ADAPT:** As one of the three domains of the central part of the KaT framework, DEVELOP or ADAPT will guide and support knowledge users to engage relevant stakeholders; to identify the existing evidence base on the chosen KT tool/product; to select a theoretical basis for the development of adaptation of the KT tool/product; to develop or adapt a functioning prototype using a user-entered design; and to conduct usability evaluation of the KT tool/product. | | | |
| --- | --- | --- | --- |
| **Sub-domain** | | | **Purpose/Description** |
| 1. **Engage relevant stakeholders** | | | Convene relevant stakeholders and knowledge users (as identified in the Discovery stage) to plan the development of the KT tool or product^3-5^. |
|  | 1. **Develop objectives and research question(s)** | | Develop focused objectives and research questions based on the problem as identified in the Discovery stage. |
|  | 1. **Select the specific KT tool or product that will be developed or adapted** | | Focus the list of potentially relevant KT tools or products as identified in the Discovery stage |
|  | 1. **If adapting an existing KT tool or product, identify the processes needed for adaptation** | | Should consider factors that may influence the fit of the tool or product such as the target population and the setting, and the technology and the resources that are needed for intervention delivery^6^; Consider using the ADAPTE tool for adaptation of clinical practice guidelines^7^ |
| 1. **Identify the existing evidence base on the chosen KT tool or product** | | | Summarize the synthesis of existing evidence on the effectiveness of the KT tool or product (if it exists) or consider conducting a knowledge synthesis if it does not. The type of knowledge synthesis method should match the research question^3-5^. |
|  | 1. Identify features and components that have potential for impact | | Use a mapping exercise to Identify features and components of tools that have potential for impact (facilitators) |
|  | 1. Create a conceptual framework of the KT tool or product | | Based on evidence and consultation with iKT team, create a conceptual framework of the KT tool or product |
| 1. **Select a theoretical basis for the development or adaptation of the KT tool or product** | | | The selection of methods will in part depend on identified gap and purpose as informed by the DISCOVERY stage. Empirical and common sense approaches exist, but evidence shows that theory driven approaches have better potential for impact and can better identify the determinants of behavior^8-10^. |
|  | 1. **Consider Research-to-Practice Models** | | Research-to-practice models include Planned Action Theories and process models to guide the development of the KT tool to promote, plan or implement change, and to make change happen, and provide guidance to address goals^8^. *NOTE: This list is not exhaustive* |
|  |  | KTA framework  *Dynamic process model* | The KTA (Knowledge-to-Action) framework is an iterative and dynamic process model that promotes an evidence-based approach to implementing knowledge. It has two components: 1) Knowledge creation and 2) action, each of which contains several phases. It is conceptualized as having no definite boundaries between the two components and their phases (i.e., steps may occur sequentially or simultaneously, and may influence each other)^3;11^. |
|  |  | MRC framework  *Methodological guidance for developing and evaluating complex interventions* | The MRC (Medical Research Council) framework of complex interventions guides researchers, funders and other decision-makers to make appropriate methodological and practical choices for the development and evaluation of complex interventions^4^. |
|  |  | NCCDPHP Knowledge to Action Framework for Public Health | CDC’s National Center for Chronic Disease Prevention and Health Promotion (NCCDPHP) Knowledge to Action (K2A) framework identifies 3 phases (research, translation, and institutionalization) and the decision points, interactions, and supporting structures within the phases that are necessary to move knowledge to sustainable action. Evaluation underpins the entire K2A process^12^. |
|  | 1. **Consider cognitive behavioural change theories or models** | | Consider behavioural cognitive theories or models as the theoretical basis for tool development when the primary goal is to change/inform behavior and attitudes *NOTE: This list is not exhaustive* |
|  |  | COM-B | COM-B (Capability, Opportunity, Motivation and Behaviour) began to identify motivation as a process that directs behavior and to address the problems of lack of integration^8;13-14^. It states that behaviour comes about from an interaction of ‘capability’ to perform the behaviour and ‘opportunity’ and ‘motivation’ to carry out the behaviour. New behaviour or behaviour change requires a change in one or more of these. COM-B states that capability, opportunity and motivation generate behaviour, which in turn influences the three components. Opportunity and capability can influence motivation, while enacting a behavior can alter capability, motivation and opportunity^14^. |
|  |  | **Social Cognitive Theory** | Social Cognitive Theory is *“a causal model whereby behavior, cognitive and other personal factors, and environmental events all operate as interacting determinants that influence each other bidirectionally*”. Personal factors can contribute to this dynamic interaction, which can be used to improve the level of organizational functioning. In particular, there are three aspects of social cognitive theory that are relevant to this: *“developing competencies through mastery modelling, strengthening people’s beliefs in their capabilities so they make better use of their talents, and enhancing self-motivation through goal systems”* ^15^. |
|  |  | **Theoretical Domains Framework (TDF)** | The TDF (Theoretical Domains Framework) outlines steps for developing a theory-informed implementation intervention: 1) Who needs to do what differently?; 2) What barriers and enablers need to be addressed? 3) Which intervention components (behavior change techniques and mode(s) of delivery) could overcome the modifiable barriers and enhance the enablers?; 4) How can behavior change be measured and understood?^16^. |
|  |  | **Theory of Planned Behaviour (TPB)** | TPB (Theory of Planned Behaviour) is a social cognitive theory that has been used widely to identify the predictors or mediators of behaviour change^17^. The premise of this theory is that “the likelihood of a person changing behavior is directly related to the strength of his or her intention to change”^17^; and has been identified as ideal for studying the changing of provider behavior in health contexts^9^. |
|  |  | **Theory of Reasoned Action** | TRA (Theory of reasoned action) postulates that behavior can be predicted by an individual’s attitudes towards doing the behavior through the intervening effect of behavioural intention^18^ |
|  | **Consider Organizational level theories** | | Theories for organizational culture, organizational climate, leadership and organizational learning, which are relevant for understanding and explaining organizational influences on implementation processes^8;19^. For example, Situated Change Theory^20^ and the Institutional^21-22^ ; the use of complexity science to better understand of organizations^23-24^; and economic theories of innovative organizations^25^. |
|  | **Consider Quality Improvement (QI) approaches** | | PDSA (plan-do-study-act) cycles provide a method for structuring iterative development of change, either as a standalone method or as part of wider QI approaches, such as the Model for Improvement (MFI), Total Quality Management, Continuous QI, Lean, Six Sigma or ‘Quality Improvement^26^. |
| 1. **Develop (or adapt) a functioning prototype using a user-centered design** | | | Consider a user-centered design (UCD) to develop the prototype (or adapt an existing tool). UCD is an approach whereby tool users are central and engaged in its design and development. The stages are carried out in iterative cycles of testing and re-design (where designs are progressively adjusted and refined) and repeated until the tool meets end user needs^27^. Continuous end-user feedback and co-development as part of the design, testing, and implementation process is the focus of UCD^27^ as is the use of rigorous research methods and evaluation to ensure that user feedback is incorporated^28-29^. |
|  | 1. **Identify relevant knowledge end-users as co-developers of the KT tool** | | Iteratively engage knowledge end-users in the co-development of the tool through a multi-phased approach that may involve forming working groups with each type of knowledge user to ensure their continuous engagement. |
|  | 1. **Develop the conceptual KT tool or product** | | Develop the conceptual KT tool or product by identifying its components or adopting components of an existing tool. Balance tailoring interventions with generalizability. |
|  |  | Use knowledge synthesis findings to Identify effective tool components, features and format | Apply knowledge synthesis results in identifying or adapting effective tools and/or their components or features. Use data on facilitators to optimize tool features; and barriers data to address challenges |
|  |  | Conduct end-user consultations to obtain feedback on identified components, features, and format and how it relates to the problem | Conduct end-user consultations to obtain feedback on identified components, features, format and design and how it relates to the problem. These could be in the format of focus groups or needs assessment |
|  |  | Finalize the conceptual KT tool and its features based on evidence and end-user consultations/feedback | Triangulate knowledge synthesis and end-user consultations to finalize the conceptual KT tool or product |
|  | 1. **Assess the determinants of knowledge use of the conceptual tool** | | Identify the barriers and facilitators of the conceptual tool and its use. Methods to assess determinants may involve one-on-one interviews and focus groups, workshop discussions, observation of facilitators and barriers, online surveys with relevant knowledge users; and brainstorming by implementation researchers, record review, and consensus of opinion leaders. |
|  | 1. **Develop content** | | Develop content of the KT tool using literature review; design content flow (logic); review of content by experts and knowledge users, and any lay language translation (if applicable) |
|  |  | Conduct literature review | Conduct literature review to develop or refine content |
|  |  | Develop information architecture (logic) | Develop content flow, logic, navigation, and information structure. Card sorting can be used as a technique whereby testers organize a representative set of items into groups and then label the groups. It also provides insights about users’ mental model of an information space and can help determine the best information architecture for the tool. |
|  |  | Conduct review of content by relevant topic experts and knowledge users | Conduct review content by relevant topic/content experts and knowledge users |
|  |  | Consider lay language translation | Consider lay language translation if applicable |
|  | 1. **Adapt content to identified format and design to transform the conceptual tool into a functional prototype** | | Based on identified components, features, format and design, adapt content and design to transform the conceptual design into a functioning prototype ready for usability evaluation |
|  |  | Technology-based tools | Development of technology-based tools involve designing optimized layout through wire frame development (to conceptualize individual screens), format and navigation; and programming for optimized functioning that meets the needs of its intended end-users. |
|  |  | Paper-based | Development of paper-based tool may involving designing an optimized presentation and layout according to the needs of its intended users |
| 1. **Conduct usability evaluation of the KT tool or product** | | | Usability testing of the KT tool or product to ensure that it meets the specific needs of each knowledge end-user. There are several different techniques that can be employed to test the user interface and interaction design^30-32^. |
|  | 1. **Identify the objectives of the usability evaluation and research question(s)** | | To assess attitudes (to understand or measure people’s beliefs); to assess behaviours (what people do)^33^. |
|  | 1. **Select the method(s) of usability evaluation matched to objectives and research question** | | Studies that generate data about behaviours or attitudes based on direct observation are qualitative (and therefore better suited to answer questions about the “why” or “how” to fix a problem), and those based on indirect observation are collected quantitatively (e.g., surveys) and better suited to answer questions about “how many” and “how much”, which can help prioritize resources (i.e., to focus on issues with the biggest impact^33^. |
|  |  | **A/B testing** | A/B testing (i.e., multivariate testing or live testing), is a method used for evaluating websites, whereby changes to a site’s design are presented to random samples of site visitors, but hold everything else constant to determine the effect of different site-design choices on behaviour. |
|  |  | **Card sorting** | Useful for testing a taxonomy or navigation structure where testers organize a representative set of items into groups and then label the groups. It also provides insights about users’ mental model of an information space and can help determine the best information architecture for the tool. |
|  |  | **Eyetracking** | To better understand how users visually interact with interface designs. An eyetracking device is configured to precisely measure where participants look as they perform tasks or interact naturally with websites, applications, physical products or environments |
|  |  | **Heuristics testing** | A checklist used by a human factors engineer to determine if the KT tool or product and its components (if applicable) meet the principles of good interaction^30;34^ and user-centered design^35-38^. Heuristic testing is best performed with multiple evaluators who have knowledge of the domain and of good interface design. |
|  |  | **Interviews** | A researcher meets with participants one-on-one to have an in-depth discuss about what the participant thinks about the topic |
|  |  | Moderated, in-person usability testing | This technique is fundamental in usability testing to obtain feedback from live users interacting with the system being tested, which can be paper prototypes to fully implemented web-based applications. Participants are brought into a lab, one-on-one with a researcher, and given a set of scenarios that lead to tasks and usage of a tool |
|  |  | Moderated focus group | Focus groups are less useful for usability purposes but can provide a top-of-mind view of what people think about a brand or concept in a group setting. Groups of participants are led through a discussion about a set of topics, and encouraged to provide verbal and written feedback |
|  |  | **Observations and ethnographic field studies** | Ethnographic field studies, which utilizes a mixture of self-reported and behavioural data. Researchers meet with and study participants in their natural environment, where they would most likely encounter the tool in question |
|  |  | **Participatory design** | Participants are given design elements or creative materials to construct their ideal experience in a concrete way that expresses what matters to them most and why |
|  |  | **Surveys** | Surveys can be used as an efficient and cost-effective way to understand the testers, and can include both open-ended and more objective assessments (via Likert scales). It can measure and categorize attitudes or collect self-reported data to help track or discover important issues or errors to address |
|  |  | **Think aloud methods** | A cognitive and usability engineering framework that involves iterative cycles of testing and redesign, whereby participants are encouraged to “think aloud” as they interact with the KT tool or product^31-32^. |
|  |  | **Usability benchmarking** | Tightly scripted usability studies are performed with several participants using precise and predetermined measures of performance. |
|  | 1. **Identify outcomes** | | Identify outcomes according to method(s) of usability evaluation |
|  | 1. **Develop evaluation instruments** | | Develop evaluation instruments according to method(s) of usability evaluation |
|  | 1. **Modify and revise tool based on usability evaluation findings in preparation for implementation and/or dissemination** | | Revise tool based on usability evaluation findings in preparation for implementation and/or dissemination. |

1. Straus SE, Tetroe J, Graham ID. Knowledge translation in health care: Moving from evidence to practice. 2nd ed. West Sussex, UK: Wiley; 2013
2. Craig, P, Dieppe P, Macintyre S, Michie S, Nazareth I, Petticrew M. Developing and evaluating complex interventions: The new Medical Research Council guidance. International Journal of Nursing Studies 2013;50:585-92.
3. Smith SM, Bayliss EA, Mercer SW, Gunn J, Vestergaard M, Wyke S, Salisbury S, Fortin M. How to design and evaluate interventions to improve outcomes for patients with multimorbidity. Journal of Comorbidity 2013;3:10–17
4. Tabak RG, Khoong EC, Chambers D, Brownson RC. Bridging Research and Practice: Models for dissemination and implementation research. Am J Prev Med 2012;43(3):337-350.
5. Fervers B, Burgers JS, Voellenger R, Brouwers M, Browman GP, Graham ID, et al. ADAPTE collaboration guideline adaptation: an approach to enhance efficiency in guideline development and improve utilization. BMJ Qualit and Safety 2011;120:228-36.
6. Nilsen P. Making sense of implementation theories, models and frameworks. Implementation Sicence 2015;10:53.
7. Colquhoun HL, Letts LJ, Law MC, MacDermid JC, Missiuna CA. A scoping review of the use of theory in studies of knowledge translation. Canadian Journal of Occumpational Therapy 2010;77:270-279.
8. Sales, A, Smith J, Curran G, Kochevar L. Models, Strategies, and Tools: Theory in implementing evidence-based findings into health care practice. J Gen Intern Med 2006;21:S43-49.
9. Graham ID, Logan J, Harrison MB, Straus SE, Tetroe J, Caswell W, Robinson N. Lost in knowledge translation: time for a map? Journal of Continuing Education in the Health Professions 2006 Winter;26(1):13-24.
10. Wilson KM, Brady TJ, Lesesne C, on behalf of the NCCDPHP Work Group on Translation. An organizing framework for translation in public health: the knowledge to action framework. Prev Chronic Dis. 2011;8:A46.
11. Michie S, van Stralen MM, West R. The behavior change wheel: A new method for characterizing and designing behavior change interventions. *Implementation Science* 2011;6:42
12. Michie, S., Atkins, L. & West, R. (2014). The behaviour change wheel: a guide to designing interventions. Silverback Publishing.
13. Bandura A. Organisational applications of Social Cognitive Theory. Australian Journal of Management 1988;13(2):275-302.
14. French SD, Green SE, O’Connor DA, McKenzie JE, Francis JJ, Michie S, Buchbinder R, Schattner P, Spike N, Grimshaw JM. Developing theory-informed behaviour change interventions to implement evidence into practice: a systematic approach using the Theoretical Domains Framework Implement Sci. 2012; 7: 38.
15. Ajzen I. The theory of planned behavior. Organizational Behaviour and Human Decision Processes 1991;50:179-211.
16. Fishbein M, Ajzen I. Belief, Attitude, Intention, and Behaviour. New York: John Wiley; 1975.
17. Estabrooks CA, Thompson DS, Lovely JE, Hofmeyer A. A guide to knowledge translation theory. J Contin Educ Health Prof. 2006;26:25–36.
18. Orlikowski W. Improvising organizational transformation over time: a situated change perspective. Inform Syst Res. 1994;7:63–92.
19. DiMaggio PJ, Powell WW. The New Institutionalism and Organizational Analysis. Chicago, IL: University of Chicago Press; 1991.
20. Scott WR. Institutions and Organizations. Thousand Oaks, CA: Sage; 1995.
21. Plsek PE, Greenhalgh T. The challenge of complexity in health care. BMJ. 2001;323:625–8.
22. Waldrop MM. Complexity: The Emerging Science at The Edge of Order and Chaos. London: Viking; 1992.
23. Grol R, Wensing M, Eccles M. Improving Patient Care: The Implementation of Change in Clinical Practice. Edinburgh: Elsevier; 2005.
24. Taylor MJ, McNicholas C, Nicolay C, Darzi A, Bell D, Reed JE. Systematic review of the application of the plan-do-study-act method to improve quality in health care. BMJ Qual & Safety 2013;0:1-9.
25. Devi K, Sen A, Hemachandran K. A working framework for the user-centered design approach and a survey of the available methods. International Journal of Scientific and Research Publications. 2012;2:1–8. <http://www.ijsrp.org/research_paper_apr2012/ijsrp-apr-2012-05.pdf>.
26. Collins A, Joseph D, Bielaczyc K. Design research: theoretical and methodological issues. Journal of the Learning Sciences. 2004;13:15–42.
27. Steele Gray C, Khan AI, Kuluski K, McKillop I, Sharpe S, Bierman AS, Lyons RF, Cott C. Improving patient experience and primary care quality for patients with complex chronic disease using the electronic patient-reported outcomes tool: adopting qualitative methods into a user-centered design approach. JMIR Res Protoc. 2016;5:e28.
28. Nielsen, J. (1994b). Heuristic evaluation. In Nielsen, J., and Mack, R.L. (Eds.), Usability Inspection Methods, John Wiley & Sons, New York, NY
29. Kushniruk AW, Patel VL, Cimino JJ. Usability testing in medical informatics: cognitive approaches to evaluation of information systems and user interfaces. In Proc AMIA Annu Fall Symp. 1997;218–22.
30. Kushniruk AW and Patel VL. Cognitive and usability engineering methods for the evaluation of clinical information systems. J Biomed Inform 2004;37:56-75.
31. Rohrer C. When to use which user-experience research methods, 2014. Available at: <https://www.nngroup.com/articles/which-ux-research-methods/>
32. Brook J: In SUS: A “quick and dirty” usability scale. Edited by: Jordan PW, Thomas B,Werdmester BA, McClelland Al. Usability Evaluation in Industry. London: Taylor and Francis; 1996.
33. Bangor A, Kortum PT, Miller JT. An empirical evaluation of the system usability scale. Inter J of Human-Com Interaction. 2008;24(6):574–94.
34. Fogg B. A behavior model for persuasive design. [2012-11-03]. Available at: http://bjfogg.com/fbm_files/page4_1.pdf. Accessed: October 2016.
35. Chan J, Shojania KG, Easty AC, Etchells EE. Does user-centred design affect the efficiency, usability and safety of CPOE order sets? J Am Med Inform Assoc 2011 May 1;18(3):276-281 [improve usability and implementation’
36. Kujala S. User involvement: A review of the benefits and challenges. Behav Inf Technol 2003 Jan;22(1):1-16.

| **DISSEMINATE:** As one of the three domains of the KaT framework, DISSEMINATE or end-of-grant KT involves sharing research results and messages with the appropriate knowledge users or audiences by tailoring the research results and messages as well as the medium with which to deliver them (and by whom) according to the needs of each type of audience. Examples of dissemination activities include summaries or briefings to stakeholders, educational sessions with patients, practitioners and/or policy makers, engaging knowledge users in developing and executing dissemination/implementation plan, tools creation, and media engagement. | | | |
| --- | --- | --- | --- |
| **Sub-domain** | | | **Purpose/Description** |
| 1. **Engage stakeholders for all steps** | | | Engage all relevant stakeholders throughout |
| 1. **Determine the goals of the dissemination and uptake** | | | Goals should be clear, concrete and well justified and appropriate to the research findings and knowledge users. Goals of dissemination include: To share research findings with the goal of increasing, generating, sharing knowledge and awareness; informing or changing attitudes, behaviour, practice, policy, or technology^1;3;95-96^. |
| 1. **Design dissemination plan (i.e., end-of-grant KT plan)** | | | The researcher develops and implements a plan for making potential knowledge users or audiences aware of the knowledge that is gained during the project^1;3;95-96^. |
|  | 1. **Determine appropriate level of dissemination** | | Appropriateness is the most important consideration for all KT activities as each discipline, project, setting, and knowledge user group is different. Successful dissemination plans are those with the best match between the expected research findings, targeted knowledge users and the selected KT strategies. Consider the reliability, validity, strength and significance of the knowledge that resulted from the research. Key messages should be clearly identified and strategies selected to address goals. In general less emphasis should be placed on dissemination of findings from small studies, those with poor methodological quality, or low strength of evidence. |
|  |  | Diffusion (let it happen) | Passive, unplanned, uncontrolled efforts to communicate information using delivery mechanisms that require little customization to reach target audiences that seek out research evidence. The onus is on the knowledge user to access, appraise and apply the knowledge. Modest approaches of dissemination are recommended in instances where generalizability of the results are limited; single studies; pilot investigations. Examples of diffusion strategies include presentations, publications, web-based activities (blogs, webcasts) |
|  |  | Dissemination (help it happen) | Also known as end-of-grant KT, dissemination is a more tailored approach to the communication of research results to a particular audience, and messaging that are adapted to the audience type or context in which the knowledge will be used. Examples include patient decision aids, educational materials, small-group meetings, plain-language summaries, reminders, social media, networks, knowledge broker involvement. |
|  |  | Application (make it happen) | Represents more intensive approaches to move research into practice such as with synthesized evidence, which is considered more robust and represents the best knowledge for widespread application. This is the most tailored communication of knowledge. Examples include working with knowledge users to adapt knowledge for use, commercialize, identify barriers to knowledge use, tailor messages, monitor knowledge use evaluate outcomes, ensure sustainability |
|  | 1. **Consider the nature of the target audience** | | Ensure that the plan considers all potentially relevant knowledge users, and that the audiences should be precisely defined (roles, responsibilities, decision making needs) and their knowledge needs and preferences identified^1;3^. |
|  | 1. **Consider the expertise of the team to achieve goals** | | Description of the team’s ability to execute the plan and to collaborate with members of the target audience^3;95^. |
|  | 1. **Identify the determinants of dissemination** | | Identification of the barriers and facilitators to the effective sharing of knowledge (e.g., communications policy of the organization, language of KT product or activity, relationships, perception of the information). Consider what can be done to overcome barriers [Straus, Graham, Tetroe, 2013; PHAC 2012; CIHR 2012] |
|  | 1. **Adapt the knowledge to each specific audience** | | The knowledge to be disseminated should be adapted according to the specific audience (language, medium)^1;3;96^ |
|  | 1. **Determine what should be the message** | | Types of messages include data, findings, conclusions, recommendations, guideline, etc.^1;3^ |
|  | 1. **Determine the format(s) the message(s) should be presented** | | The most appropriate ways to present knowledge may be different for different knowledge users (e.g., report, summary, fact sheet, diagram, case study, podcast, poster, video)^3^ |
|  | 1. **Determine how the message(s) should be shared or delivered** | | Knowledge can be delivered in different ways for different knowledge users (e.g., mail-outs, website, social media, email, community of practice, discussion forum, conference presentation, training session, etc.) |
|  | 1. **Identify who should deliver the message(s)** | | Identifying the most appropriate person to deliver the message can influence knowledge use (e.g., credibility of the messenger for that knowledge user group)^1;3^ |
|  | 1. **Consider the context in which the knowledge is to be used** | | The plan should take into consideration the context in which the knowledge is to be used. This may also involve finding opportunities for sharing the knowledge (e.g., existing bulletin or newsletter, upcoming events, committee or network, team meeting, one-on-one contact, etc.)^1;3;96^ |
|  | 1. **Consider the resources (human, consumables)** | | Resources can involve personnel involving time, skills, responsibilities, roles, cultural competence across different expertise (e.g., clinical content, information technology, human factors, cost analysis, biostatistics, KT methods, knowledge broker) and financial such as consumables (e.g., costs related to printing, teleconferences, meetings, publishing, web-based, software, editing, translation). The consideration of the budget to ensure that there sufficient financial support to implement the dissemination plan^1;3;96^ |
|  | 1. **Consider using a framework for dissemination and spread** | | Consider using a framework for dissemination and spread: Framework for Spread^97^; Health Promotion Research Center Framework^98^ ; Linking Systems Framework^99^; PDSA (plan-do-study-act), which provides a method for structuring iterative development of change, either as a standalone method or as part of wider quality improvement approaches such as the Model for Improvement (MFI), Total Quality Management, Continuous QI, Lean, Six Sigma or ‘Quality Improvement^26^; Wave Sequence Spread, which is a systematic approach to rapidly spread multi-level interventions (i.e., interventions that cross tertiary, secondary, and primary care settings and might even branch into the community). This approach builds on the collaborative improvement approach and emphasizes developing champions from within the system to carry out the subsequent spread^100^; and the National Institutes of Health (NIH) approaches to dissemination and implementation^62^. |
| 1. **Monitor and Evaluate dissemination and uptake** | | | Monitor and assessment of the impact of knowledge dissemination and uptake^1;3;96^ |
|  | 1. **Identify the methods to monitor and evaluate knowledge use and uptake** | | The methods to monitor/evaluate the knowledge use and uptake of KT tools and products may include questionnaires, interviews, website metrics, experimental studies. |
|  | 1. **Identify indicators** | | Indicators for meeting the goals of the dissemination (reach, usefulness, use); and reaching the right target audience |
|  |  | Indicators that measure reach | Primary distribution (knowledge is pushed to the audience); Secondary distribution (audience pulls or requests the knowledge; examples: number of website hits, number of products distributed in response to order, number of people reached) |
|  |  | Indicators that measure usefulness | User satisfaction (% who received knowledge; % satisfied, % that rate the format or presentation as usable, number/% who rate the content as useful, % who report knowledge gained from it, number/% who report that it changed their views) |
|  |  | Indicators that measure use | Number/% of people intending to use the knowledge, number/% of people adapting the knowledge, number/% of people using the knowledge to inform practice/policy, number/% of people using the knowledge to improve their own practice or performance |
|  | 1. **Identify who should monitor/evaluate** | | Identify who should monitor/evaluate |

1. Canadian Institutes of Health Research (CIHR): Guide to Knowledge Translation Planning at CIHR: Integrated and End-of-Grant Approaches; 2012. Available at: <http://www.cihr-irsc.gc.ca/e/45321.html>.
2. Straus SE, Tetroe J, Graham ID. Knowledge translation in health care: Moving from evidence to practice. 2nd ed. West Sussex, UK: Wiley; 2013.
3. Taylor MJ, McNicholas C, Nicolay C, Darzi A, Bell D, Reed JE. Systematic review of the application of the plan-do-study-act method to improve quality in health care. BMJ Qual & Safety 2013;0:1-9.
4. Glasgow RE, Vinson C, Chambers D, Khoury MJ, Kaplan RM, Hunder C. National Institutes of Health approaches to dissemination and implementation sciences: Current and future directions. AJPH 2012;102(7):1274-81.
5. Barwick M. 2010. Knowledge Translation Planning Template. Hospital for Sick Children, Toronto, Ontario. Available at: <http://melaniebarwick.com/training.php>.
6. Public Health Agency of Canada: Knowledge Translation (KT) Planning Primer 2012. Available at: <http://publications.gc.ca/collections/collection_2013/aspc-phac/HP35-37-2012-eng.pdf>
7. Nolan, K, Schall MW, Erb F, Nolan T. Using a Framework for Spread: The case of patient access in the veterans health administration. Journal on Quality and Patient Safety 2005;31(6):339-347.
8. Harris JR, Cheadle A, Hannon PA, Forehand M, Lichiello P, Mahoney E, et al. A framework for disseminating evidence-based health promotion practices. Prev Chronic Dis. 2012;9:E22.
9. Robinson K, Elliott SJ, Driedger SM, et al. Using linking systems to build capacity and enhance dissemination in heart health promotion: a Canadian multiple-case study. Health Educ Res 2005;20(5):499–513.

| **IMPLEMENT:** Implementation is defined as *“the initial process of embedding tools in a setting”* [Chambers 2013]. As one of the three domains of the KaT framework, IMPLEMENT will guide and support knowledge users to engage with relevant stakeholders to determine their objectives for implementation, define roles and optimized communication mechanisms; to identify the implementability of the KT tool/product; to develop and execute the implementation plan; to monitor and evaluate knowledge use; and to organize and document findings using appropriate reporting criteria*.* | | | |
| --- | --- | --- | --- |
| **Sub-domain** | | | **Purpose/Description** |
| 1. **Engage relevant stakeholders and establish partnerships to:** | | | Convene relevant stakeholders, partners and knowledge users (building on individuals identified in the Discovery stage) to plan the implementation, monitoring, and evaluation of the KT tool or product |
|  | 1. **Determine the objectives and goals for implementation and refine goals** | | Determine the objectives for implementation. |
|  | 1. **Determine optimized communication mechanism among team** | | Define strategies that will be used to establish optimized communication amongst the team, and to enable efficient modes of communication and exchange of information (e.g., in-person, email, tele-/web-conference, newsletter); |
|  | 1. **Identify and clarify roles** | | Identify and clarify roles |
|  | 1. **Identify any anticipated challenges and mitigating strategies to implementation** | | Identify any anticipated challenges and mitigating strategies to implementation |
| 1. **Identify the implementability of the KT tool/product** | | | Identify the implementability of the KT tool or product: determinants of implementation from the perspective of relevant knowledge users including their readiness to change |
|  | 1. **Assess the determinants of implementation (barriers and facilitators to change)** | | Identify the barriers and facilitators to change and the perceived relevance, acceptability and feasibility of the implementation of a KT tool/product. These can be assessed using interviews, surveys and focus groups involving each type of identified knowledge user^3^. |
|  | 1. **Assess Readiness to Change** | | Organizational readiness for change is defined as “the extent to which organizational members are both psychologically and behaviorally prepared to implement change”^39^. Assessment of readiness provides an opportunity to identify potential challenges and can lead to better implementation outcomes. An organization is more likely to accept the change if they are ready. However, organizations, systems and people may not be ready to implement KT tools/products even if these are effective and implementation is feasible. As such, tools are available to assess readiness to adopt innovations in health care. Understanding whether knowledge users are ready for change is key to success. Low readiness may require a phased approach to implementation (gradual introduction of the KT tool/product into the organization, system or community). |
|  |  | **Organizational readiness to change strategies**  *General* | ORCA (Organisational Readiness for Implementing Change Measures) based on the PARIHS framework, and was developed for use in quality improvement activities by researchers to assess site readiness. It includes three domains (evidence, context, and facilitation), and has adequate estimates of reliability and validity^40^; ORIC (Organisational Readiness for Implementing Change) measure is based on Weiner’s theory of organizational readiness for change^41^; ORC^42-44^ and extended ORC^42;45^; TCU-ORC^42^; TCU-ORC-D ^46^; FORCE (functional ORC evaluation)^47^; MORC (medical ORC)^48^; PORC (perceived ORC)^49^; OCRS (organization culture and readiness survey^50^; Organizational readiness for stage-based dynamics of innovation implementation^51^. |
|  |  | **Technology readiness to change strategies**  *e-Health, telehealth, CPOE* | OITIRIS (Information technology innovation readiness^52^; Organizational eHealth readiness^53^; organizational telehealth readiness^54^; CPOE readiness^55^ |
|  |  | **I-RREACH:**  *To guide implementation in low-resource settings* | I-RREACH (Implementation and research readiness engagement and assessment of community health care) tool is aimed at guiding implementation interventions in low-resource environments^56^. |
|  |  | **MORE scale**  *Organizational readiness for patient engagement* | MORE (Measuring Organisational Readiness for Patient Engagement) is a theory-based measure of organisational readiness focused on patient engagement. It has been developed using an international group of knowledge users (16 countries) but it has not yet been validated^57^. |
|  |  | **PRECEDE-PROCEDE model** | The PRECEDE-PROCEED (Predisposing, Reinforcing and Enabling Constructs in Educational Diagnosis and Evaluation-Policy, Regulatory, and Organizational Constructs in Educational and Environmental Development) specify implementation aspects that should be evaluated as part of intervention studies^58^. |
|  |  | **Ready, Set, Change!**  *Decision Support Tool to guide the selection of a reliable readiness assessment measure* | *Ready, Set, Change!* is a decision support tool, which provides guidance to implementers to select a valid and reliable readiness assessment measure that is appropriate for their settings^59-60^. Although measures are available to help teams assess readiness, the selection of these measures have generally been found to be difficult and therefore not used^59^. *Ready, Set, Change!* is based on a framework for organizational readiness for change consisting of 4 key constructs^61^: “1) Individual Psychological (attitudes, beliefs and perceptions held by individuals regarding the change); 2) Individual Structural (staff knowledge, skills and abilities to perform activities and roles related to the change); 3) Organizational Psychological (the extent to which members of an organization are seen to work together to achieve change implementation); 4) Organizational Structural (human and material resources, communication channels, and formal policies required to support change implementation)”. |
| 1. **Develop implementation plan** | | | Create a plan for implementation according to identified implementation objectives and readiness |
|  | 1. Identify stakeholders who should be involved in the implementation | | Identify stakeholders who should be involved in the implementation |
|  | 1. Consider the use of an implementation framework | | Consider the use of an implementation framework to guide the implementation process and to achieve enhanced understanding and explanation of certain aspects of implementation. Implementation strategies should address barriers to implementation and readiness to change and match objectives and scope^8;62-63^. *[NOTE: This list is not exhaustive]* |
|  |  | **CFIR framework:**  *Addresses the implementation of innovations in general* | The CFIR (Consolidated Framework for Implementation Research) comprises 5 major domains (the intervention, inner and outer setting, the individuals involved, and the process by which implementation is accomplished) – these domains interact in rich and complex ways to influence implementation effectiveness^64^. CFIR addresses many different components and factors that can influence the use of research in decision making. It is grounded in published theories that have been evaluated to identify constructs based on strength of conceptual or empirical support; it provides explicit definitions for each construct, but doesn’t provide guidance for how to progressively implement innovations^64-65^. |
|  |  | **Diffusion of Innovations** | Rogers’ Theory of Diffusion is considered the single most influential theory in the field of knowledge utilization and has been widely applied in implementation science^8^. Its attributes include: relative advantage, compatibility, complexity, trialability and observability^8;66^. Additionally, it highlights the importance of intermediary actors (opinion leaders, change agents and gatekeepers) for successful adoption and implementation^66^. |
|  |  | **Diffusion of Innovations in Service Organizations** | Greenhalgh et al added constructs from social psychology, organizational behavior theories, and socio-technical systems theory to produce a typology of factors that affect diffusion into practice^67^. |
|  |  | **GItools**  *Framework for developing guideline implementability tools* | GItools (Guideline Implementatbility Tools) is a 12-item framework that characterizes the optimal features of GI tools^68^. |
|  |  | **KTA framework**  *Dynamic process model to guide the implementation of knowledge* | The KTA (Knowledge-to-Action) framework is an iterative and dynamic process model that promotes an evidence-based approach to implementing knowledge. It has two components: 1) Knowledge creation and 2) action, each of which contains several phases. It is conceptualized as having no definite boundaries between the two components and their phases (i.e., steps may occur sequentially or simultaneously, and may influence each other)^3;11^. |
|  |  | **Linking RTA**  *Assesses country level efforts linking research to action* | Linking RTA (Linking Research to Action) provides a range of activities that can be considered when developing initiatives within organizations to support the use of research evidence to inform health policy decisions^69^. The framework includes four elements: the climate for research use, the production of research and appropriate synthesis of research for policymakers, efforts used to link research to action, and evaluation. |
|  |  | **NIH approaches to dissemination and implementation** | National Institutes of Health (NIH) approaches to dissemination and implementation sciences^70^. |
|  |  | **Normalization Process Theory**  *Facilitates embedding complex interventions in practice* | The Normalization Process Theory was a model aimed for the implementation of new technologies, and was expanded into a theory as change mechanisms and interrelations between various constructs were clarified. The theory identifies four determinants of embedding (i.e. normalizing) complex interventions in practice (coherence or sense making, cognitive participation or engagement, collective action and reflexive monitoring) and the relationships between these determinants^71-72^. |
|  |  | **OMRU**  *Facilitates research use in clinical practice settings* | OMRU (Ottawa Model of Research Use) was developed in response to improving the use of research evidence in clinical practice. It consists of six key elements: evidence-based innovation (e.g. a continuity of care innovation), potential adopters (those whose behaviours are intended to change), the practice environment (settings, sectors), implementation of interventions, adoption of the innovation, and outcomes resulting from implementation of the innovation (e.g. patient, practitioner, economic and system implications)^73^. |
|  |  | **PARIHS framework:**  *Examines different dimensions of context on research use to explicitly include facilitation to effect research use* | The PARIHS (Promoting Action on Research In Health Sciences) framework is a conceptual heuristic for research implementation. It has 3 elements (evidence, context, and facilitation) and each of these is ranked on a low-to-high scale, whereby the most successful implementation occurs when all elements are on the scale’s high end^74-75^. It is a flexible framework but it is complex and remains largely untested it terms of how it can guide research use in an actual practice environment rather than as a reflective assessment tool. The premise of the PARIHS framework is that for implementation of evidence to be successful, there needs to be clarity about the nature of the evidence being used the nature of the context, and the type of facilitation needed to ensure a successful change process^75-76^. |
|  |  | **PRISM model**  *Implementing evidence-based practices in public health* | The Practical, Robust Implementation and Sustainability Model (PRISM) model uses concepts from the quality improvement, chronic care, and the diffusion of innovations along with measures of population-based effectiveness interventions. It includes key elements to consider when implementing evidence-based practices and key questions to enhance implementation and sustainability for each domain. The domains include: Program (Intervention) – organizational and population perspectives of the intervention; External environment; Implementation and sustainability infrastructure; Recipients (organizational and population characteristics)^77^. |
|  |  | **RAPID model**  *Understand knowledge use and translation of policy in developing countries* | The RAPID (Research and Policy in Development) model was developed to understand the dynamics of knowledge use and translation in the policy realm in developing countries^78^. The RAPID model includes four aspects: 1) the political context extent (i.e., civil and political freedoms, institutional pressures, vested interests, power relations, and attitudes and incentives among officials), 2) characteristics of the evidence (quality of the research, the relevance of the topic, the operational usefulness of an idea, and the solutions or recommendations associated with the research), 3) links between policy and research communities (i.e., the research and policy communities through intermediary organizations or networks), and 4) external influences (international policies as well as socio-economic and cultural influences). The messengers, communication, and packaging of the research are important to consider as well^78^. |
|  |  | **QIT**  *Tool to facilitate quality implementation of an innovation* | The QIT (Quality Implementation Tool) was developed using the interactive systems framework for dissemination and implementation (ISF)^79^, which focuses on the interactions between multiple systems that collaboratively build the capacity needed to disseminate and implement an innovation with quality^79^. The QIT tool is aimed at enhancing the likelihood that desired outcomes are achieved^80^. It can be applied to planning for quality implementation, real-time monitoring of implementation, and evaluating the extent to which the innovation was implemented with quality. It can be used to facilitate high quality planning, monitoring and evaluation of how an innovation is implemented^80^. “The QIT is designed to be completed through a collaborative process between members of the Support and Delivery Systems.” |
|  |  | **RD & U framework**  *Research dissemination and utilization for health policy and clinical decision making* | The RD & U (Research Dissemination and Utilization) framework was developed to support public health practitioners^81^. This framework incorporates the complex interrelationships that exist among the five stages of Rogers’ Diffusion of Innovation theory^66^: knowledge, persuasion, decision, implementation and confirmation. The framework takes into consideration the influence of characteristics associated with the innovation, organization, environment and individual. The framework progresses from research dissemination to research utilization and provides examples of potential types of research dissemination, evidence based decision making, research utilization and outcomes^81^. |
|  |  | **TAM**  *Technology Acceptance Model* | TAM (technology acceptance model) is based on the Theory of Planned Behaviour by including a group of “diffusion constructs”^82^; TAM has been shown to be predictive of intention to utilize behaviours, interventions and innovations among physicians^83^. |
|  |  | **TICD**  *Tailored Implementation for Chronic Diseases* | The TICD (Tailored Implementation for Chronic Diseases) checklist helps **to** develop valid and efficient methods of tailoring implementation interventions to determinants of practice for knowledge implementation in chronic illness care^84^. |
|  |  | **TDF**  *Steps to developing theory-informed implementation* | The TDF (Theoretical Domains Framework) outlines steps for developing a theory-informed implementation intervention: 1) Who needs to do what differently?; 2) What barriers and enablers need to be addressed? 3) Which intervention components (behavior change techniques and mode(s) of delivery) could overcome the modifiable barriers and enhance the enablers?; 4) How can behavior change be measured and understood?^16^. |
|  |  | **WHO Ageing and Health KT Framework**  *Facilitates the use of evidence in policymaking in the area of ageing and health* | This framework is based Lavis’ Linking RTA framework^69^, and includes other factors deemed important to the field of ageing and health (i.e. context), and to reflect the order of importance of the different elements with respect to facilitating the use of research evidence in policymaking in the area of ageing and health^76^. This framework includes contextual factors, relationships, and initiatives specific to ageing and health. The main elements are: 1) a climate and/ or context for research use, 2) linkage and exchange efforts between researchers, stakeholders and knowledge users, 3) creation of new knowledge, 4) push efforts, 5) facilitating pull efforts, 6) pull efforts, and 7) evaluation of efforts to link research to action^76^. |
| 1. **Monitor and Evaluate the implementation of the KT tool or product** | | | This step is necessary to determine how and to what extent the knowledge is used by knowledge users. There is a need to assess how well the implementation of the tool is going and to make corrections if needed. In these stages, there are many evaluation questions that could be asked, all having to do with monitoring and evaluation activities. Depending on the purpose of the evaluation and implementation objectives, create an evaluation plan |
|  | 1. **Engage stakeholders and knowledge users** | | Engage stakeholders and knowledge users to identify the objectives and purpose of the evaluation, to select the appropriate study designs, to select outcomes, and the best approaches to evaluation. Engage experts according to identified objectives and purpose such as in the relevant content area, biostatistics, health economic analysis, behavior theory, KT, etc. |
|  | 1. **Identify objectives and purpose of the evaluation** | | Identify the objectives and purpose of the evaluation. Setting the objectives for the evaluation will help to focus it and keep the process from becoming too complex and all-inclusive. |
|  |  | Formative evaluation | Evaluate the feasibility, appropriateness, and acceptability of the KT tool or product before it is fully implemented. This is usually conducted when a new tool is being developed or when an existing one is being adapted or modified |
|  |  | Implementation or process evaluation | Determine whether the KT tool or program have been implemented as intended. Process evaluation includes the identification of the target population, a description of the services delivered, the use of resources, and the qualifications and experiences of the personnel participating in them. It involves determining what services were actually delivered, to whom, and with what level of resources. |
|  |  | Outcome evaluation | Objective-based evaluation after the KT tool or product has made contact with at least one person or group in the target population (i.e., the degree to which the program is having an effect on the target population’s behaviours); it informs whether the KT tool or product is effective in meeting its objectives |
|  |  | Economic evaluation | Cost analysis, cost-effectiveness evaluation, cost-benefit analysis, cost-utility analysis at the beginning or during the conduct of the study; it will show what resources are being used and their costs (direct and indirect) compared to outcomes; It provides a way to assess cost relative to effects. |
|  |  | Impact evaluation | Assesses the effectiveness in achieving outcomes at the end of the study; provides evidence for use in practice or policy and funding decisions |
|  | 1. **Select the appropriate study design(s) for the type of evaluation** | | Select the most appropriate study design or method to address research question(s) and purpose of evaluation. Consider whether the evaluation is for local or generalizable knowledge |
|  |  | **RCTs**  *Broad evaluation to understand the “what”* | Consider explanatory RCTs (efficacy) or pragmatic RCTs (effectiveness) for broader evaluation with larger samples sizes, more settings. RCTs should be consider after pilot evaluation has been completed and findings show potential for impact. Could be considered as part of a dissemination or scale-up strategy. Advantages: high internal validity, generalizability; Limitations: resource intensive, may not be generalizable if using a pragmatic approach |
|  |  | **Non-randomized or quasi-experimental studies:**  *Feasibility and pilot evaluation* | Consider using controlled before-after, interrupted time series, and uncontrolled before-after studies to assess feasibility of the KT tool or product (pilot studies). Advantage: Requires fewer resources and has simpler logistics. Limitations are: more potential for selection bias. |
|  |  | **Qualitative evaluation**  *To understand the “why”* | RCTs and non-randomized studies help us understand the “what” but not the “why”. Consider conducting a qualitative study to better understand “why” the KT tool or product works (or doesn’t work), for whom, and under what contexts |
|  | 1. **Select outcomes and establish indicators** | | Consider a wide range of outcomes (and indicators) to ensure that there is a distinction between assessing implementation effectiveness vs. treatment effectiveness^13^. Outcome categories include clinical, patient, process or implementation, service, organizational and healthcare system and economic^3;85^. Consider intermediate outcomes such as patient self-efficacy and health behaviours that link to longer term outcomes; Consider measures that capture treatment burden, avoidance of unnecessary tests, and minimizing medication side-effects; Consider cost-effectiveness and health-related quality-of-life measures to inform economic analyses^5^. |
|  |  | Clinical | Disease symptoms, mental state, effects of a disease or condition on patient functioning |
|  |  | Patient | Change in health status (e.g., mortality, quality of life); satisfaction, function, symptomatology; well-being, safety^3;85^; |
|  |  | Provider | Provider satisfaction, acceptance |
|  |  | Process or implementation | Accepatability; adoption, appropriateness, costs, feasibility, fidelity, penetration, sustainability^85^; |
|  |  | Service | Efficiency, safety, effectiveness, equity, patient-centeredness, timeliness^85^ |
|  |  | Organizational and health care system | Attitudes, knowledge and competencies; organizational culture and climate; policies and procedures; practices and services; use of data and technology; stakeholder awareness and engagement; relationships |
|  |  | Economic | Cost-effectiveness; health-related quality-of-life |
|  | 1. **Consider using evaluation frameworks** | |  |
|  |  | COM-B | The COM-B can be used as an evaluation framework^14;71;86^ |
|  |  | Conceptual framework for implementation outcomes | This framework describes how to conceptualize and evaluate successful implementation, and proposes a taxonomy of 8 conceptually distinct Implementation outcomes (acceptability; adoption; appropriateness; feasibility; fidelity; implementation cost; penetration; and sustainability)^85^. |
|  |  | Framework of implementation outcomes | The framework of implementation outcomes was developed by Proctor et al^85^ that can be used to evaluate implementation initiatives. It has eight conceptually distinct outcomes for potential evaluation: acceptability, adoption, (also referred to as uptake), appropriateness, costs, feasibility, fidelity, penetration (integration of a practice within a specific setting) and sustainability (also referred to as maintenance or institutionalization)^85^. |
|  |  | Normalization Process Theory | The Normalization Process Theory can be used as an evaluation framework^71-72;87^ |
|  |  | PRECEDE-PROCEED model | The PRECEDE-PROCEED (Predisposing, Reinforcing and Enabling Constructs in Educational Diagnosis and Evaluation-Policy, Regulatory, and Organizational Constructs in Educational and Environmental Development) specify implementation aspects that should be evaluated as part of intervention studies^58^. |
|  |  | RE-AIM framework | RE-AIM (Reach, Effectiveness, Adoption, Implementation and Maintenance) is a framework that offers a comprehensive approach to considering five dimensions important for evaluating the potential public health impact of an intervention^88^. RE-AIM can be used to help plan programs and improve their chances of working in “real-world” settings. The overall goal of the RE-AIM framework is to encourage knowledge users to pay more attention to essential program elements that can improve the sustainable adoption and implementation of effective, evidence-based health promotion programs. RE-AIM consists of 5 elements: reach (did the target population receive the tool/product? Establishing organizational champions can enhance reach), effectiveness (did the tool/product have its intended effect? More useful to objectively measurement increased knowledge or skill than self-reported perceptions), adoption (was the tool/product adopted by its intended users? Important to measure actual behavior change), implementation (was the intervention implemented with high fidelity to its essential features? Cues such as handouts that summarize the innovation may improve implementation), and maintenance (was the tool/product maintained in practice over long-term follow-up? Reminders about the innovation after it’s been implemented can enhance maintenance of knowledge use)^88^. To maximize overall impact, an intervention must perform well across all five elements. Despite some overlap, each of the elements has been designed to provide the necessary guidance to improve the chances of successfully adopting an evidence-based health promotion program. The RE-AIM framework includes elements related to program design at both the participant level (Reach, Effectiveness, and Maintenance) and the organizational or setting level (Adoption, Implementation, and Maintenance)^88^. |
|  |  | Theoretical Domains Framework | The Theoretical Domains Framework can be used as an evaluation framework^89-90^ |
| 1. **Organize and document findings – consider using a tool development and evaluation reporting criteria to guide this process** | | | To ensure transparency of methods, interpretation of study results and replicability of interventions, consider applying reporting criteria for the development and evaluation of KT tools or products^91^. |
|  |  | **CReDECI**  *Criteria for reporting the development and evaluation of complex interventions* | The 16-item CreDECI criteria is based on the MRC framework, and comprise a minimum set of reporting criteria to improve the transparency and quality of publications on the development and evaluation of complex interventions^91^ |
|  |  | **RAMESES II**  *Realist And Meta‐narrative Evidence Syntheses: Evolving Standards* | Reporting standards for Realist evaluation, which is a form of theory-driven evaluation that can address “what works, for whom, under what circumstances, and how” in complex interventions. The aim of the reporting standards is to “lead to greater consistency and rigour of reporting and make realist evaluation reports more accessible, usable and helpful for different stakeholders”^92^. |
|  |  | **TiDIER**  *Template for intervention description and replication checklist and guide* | The 12-item template for intervention description and replication (TiDIER) checklist encourages authors to describe interventions in sufficient detail to allow their replication. The checklist contains the minimum recommended items for describing an intervention^93^. |
|  |  | **WIDER**  *Workgroup for Intervention Development and Evaluation Research recommendations in systematic reviews* | The WIDER framework aims to identify and provide detailed reporting of the essential components of behavior change interventions in systematic reviews to facilitate replication, further development, and scale-up of the interventions^94^. |

1. Straus SE, Tetroe J, Graham ID. Knowledge translation in health care: Moving from evidence to practice. 2nd ed. West Sussex, UK: Wiley; 2013.
2. Smith SM, Bayliss EA, Mercer SW, Gunn J, Vestergaard M, Wyke S, Salisbury S, Fortin M. How to design and evaluate interventions to improve outcomes for patients with multimorbidity. Journal of Comorbidity 2013;3:10–17
3. Nilsen P. Making sense of implementation theories, models and frameworks. Implementation Sicence 2015;10:53.
4. Graham ID, Logan J, Harrison MB, Straus SE, Tetroe J, Caswell W, Robinson N. Lost in knowledge translation: time for a map? Journal of Continuing Education in the Health Professions 2006 Winter;26(1):13-24.
5. Michie S, van Stralen MM, West R. The behavior change wheel: A new method for characterizing and designing behavior change interventions. *Implementation Science* 2011;6:42
6. Michie, S., Atkins, L. & West, R. (2014). The behaviour change wheel: a guide to designing interventions. Silverback Publishing.
7. French SD, Green SE, O’Connor DA, McKenzie JE, Francis JJ, Michie S, Buchbinder R, Schattner P, Spike N, Grimshaw JM. Developing theory-informed behaviour change interventions to implement evidence into practice: a systematic approach using the Theoretical Domains Framework Implement Sci. 2012; 7: 38.
8. Jennett PA, Gagnon MP, Brandstadt HK. Preparing for success: Readiness models for rural telehealth. J Postgrad Med. 2005;51:279–85.
9. Helfrich CD, Li YF, Sharp ND, Sales AE (2009) Organizational readiness to change assessment (ORCA): development of an instrument based on the Promoting Action on Research in Health Services (PARIHS) framework. IS, 4: 38.
10. Shea CM, Jacobs SR, Esserman DA, Bruce K, Weiner BJ. Organizational readiness for implementing change: a psychometric assessment of a new measure. Implementation Sci. 2014;9:7.
11. Lehman WE, Greener JM, Simpson DD (2002) Assessing organizational readiness for change. J Subst Abuse Treat. 22(4): p. 197–209.
12. Fuller BE, Rieckmann T, Nunes EV, Miller M, Arfken C, et al. (2007) Organizational Readiness for Change and opinions toward treatment innovations. J Subst Abuse Treat, 33(2): 183–192.
13. Gotham HJ, Claus RE, Selig K, Homer AL (2009) Increasing program capability to provide treatment for co-occurring substance use and mental disorders: organizational characteristics. J Subst Abuse Treat, 38(2): 160–169.
14. Saldana L, Chapman JE, Henggeler SW, Rowland MD (2007) The Organizational Readiness for Change scale in adolescent programs: Criterion validity. J Subst Abuse Treat. 33(2): p. 159–69.
15. Chabot G, Gagnon M-P, Godin G (2012) Redefining the school nurse role: an organizational perspective. J Health Organ Manag. 26(4): p. 444–466.
16. Devereaux MW, Drynan AK, Lowry S, MacLennan D, Figdor M, et al. (2006) Evaluating organizational readiness for change: a preliminary mixed-model assessment of an interprofessional rehabilitation hospital. Healthc Q, 9(4): 66–74.
17. Bohman TM, Kulkarni S, Waters V, Spence RT, Murphy-Smith M, et al. (2008) Assessing health care organizations’ ability to implement screening, brief intervention, and referral to treatment. Journal of Addiction Medicine, 2(3): 151–157.
18. Armenakis AA, Harris SG, KW M (1993) Creating readiness for organizational change. Human Relations, 46(6): 681–703.
19. Melnyk B, Fineout-Overholt E, Mays M (2008) The evidence-based practice beliefs and implementation scales: psy- chometric properties of two new instruments. Worldviews Evid Based Nurs, 5(4): 208–216.
20. Simpson DD. Organizational readiness for stage-based dynamics of innovation implementation. Res Social Work Practice. 2009; 19(5):541–551.
21. Snyder-Halpern R (2002) Development and pilot testing of an Organizational Information Technology/Systems Innovation Readiness Scale (OITRIS). Proc AMIA Symp 702–706.
22. Toure M, Poissant L, BR (2012) Assessment of organizational readiness for e-health in a rehabilitation centre. Disabil Rehabil, 34(2): 167–173.
23. Jennett P, Bates J, Healy T, Ho K, Kazanjian A, et al. (2003) A readiness model for telehealth is it possible to pre-determine how prepared communities are to implement telehealth? Stud Health Technol Inform, 97: 51–55.
24. Stablein D, Welebob E, Johnson E, Metzger J, Burgess R, Classen DC (2003) Understanding hospital readiness for computerized physician order entry. Jt Comm J Qual Saf, 29(7): 336–344.
25. Maar M, Yeates K, Barron M, Hua D, Liu P, Lum-Kwong MM, Perkins N, Sleeth J, Tobe J, Wabano MJ, Williamson P, Tobe SW. I-RREACH: an engagement and assessment tool for improving implementation readiness of researchers, organizations and communities in complex interventions. Implementation Science 2015;10:64.
26. Oostendorp LJM, Durand MA, Lloyd A, Elwyn G. Measuring organizational readiness for patient engagement (MORE): an international online Delphi consensus study. BMC Health Services Research 2015;15:61.
27. Green, L., Kreuter, M. (2005). Health program planning: An educational and ecological approach. 4th edition. New York, NY: McGraw-Hill
28. Timmings C, Khan S, Moore JE, Marquez C, Pyka K, Straus SE. Ready, Set, Change! Development and usability testing of an online readiness for change decision support tool for healthcare organizations. BMC Med Inform Dec Mak 2016;16:24.
29. Khan S, Timmings C, Moore JE, Marquez C, Pyka K, Gheihman G, et al. The development of an online decision support tool for organizational readiness for change. Implement Sci. 2014;9:56.
30. Holt DT, Helfrich CD, Hall CG, Weiner BJ. Are you ready? How health professionals can comprehensively conceptualize readiness for change. J Gen Intern Med. 2010;25(1):50–5.
31. Glasgow RE, Vinson C, Chambers D, Khoury MJ, Kaplan RM, Hunder C. National Institutes of Health approaches to dissemination and implementation sciences: Current and future directions. AJPH 2012;102(7):1274-81.
32. The Improved Clinical Effectiveness through Behavioural Research Group (ICEBeRG). Designing theoretically-informed implementation interventions 2006;1:4.
33. Damschroder LJ, Aron DC, Keith RE, Kirsh SR, Alexander JA, Lowery JC. Fostering implementation of health services research findings into practice: a consolidated framework for advancing implementation science. Implementation Science 2009; 4(50).
34. Damschroder L, Hall C, Gillon L, Reardon C, Kelley C, Sparks J, Lowery J. The Consolidated Framework for Implementation Research (CFIR): progress to date, tools and resources, and plans for the future. Implementation Science 2015, 10(Suppl 1):A12
35. Rogers EM. Diffusion of Innovations. 5th ed. New York: Free Press; 2003.
36. Greenhalgh T, Robert G, Macfarlane F, Bate P, Kyriakidou O. Diffusion of Innovations in service organizations: Systematic Review and recommendations. Millbank Q 2004;82(4):581-629.
37. Gagliardi AR, Brouwers MC, Bhattacharyya O and the Guideline Implementation Research and Application Network. Implementation Science 2014;9:98.
38. Lavis JN, Lomas J, Hamid M, Sewankambo NK. Assessing country-level efforts to link research to action. Bulletin of the World Health Organization 2006; 84(8):620-628.
39. National Institutes of Health (NIH) approaches to dissemination and implementation sciences: Current and future directions. AJPH 2012;102(7):1274-81.
40. May C, Finch T. Implementing, embedding and integrating practices: an outline of Normalization Process Theory. Sociology. 2009;43:535-54.
41. Murray E, Treweek S, Pope C, et al. Normalisation process theory: a framework for developing, evaluating and implementing complex interventions. BMC Med 2010;8:63.
42. Graham ID, Logan J. Innovations in knowledge transfer and continuity of care. The Canadian Journal of Nursing Research 2004; 36(2):89-103.
43. Kitson AL, Rycroft-Malone J, Harvey G, McCormack B, Seers K and Titchen A. Evaluating the successful implementation of evidence into practice using the PARiHS framework: theoretical and practical challenges. Implementation Science 2008;3:1
44. Stetler, C.B., Damschroder, L.J., Helfrich, C.D. et al. (2011). A Guide for applying a revised version of the PARIHS framework for implementation Implement Sci. 2011; 6: 99.
45. Ellen M. Knowledge Translation Framework for Ageing and Health. April 2012. Available at: http://www.who.int/ageing/publications/knowledge_translation.pdf
46. Feldstein, A. C. & amp; Glasgow, R. E. (2008). A Practical, Robust Implementation and Sustainability Model (PRISM) for integrating research findings into practice. The Joint Commission Journal on Quality and Patient Safety, 34(4), 228-243.
47. Jones N, Datta A, Jones H. Knowledge, policy and power: Six dimensions of the knowledge–development policy interface. 2009. Overseas Development Institute.
48. Wandersman, A., Duffy, J., Flaspohler, P., Noonan, R., Lubell, K., Stillman, L., et al. Bridging the gap between prevention research and practice: The Interactive Systems Framework for Dissemination and Implementation. American Journal of Community Psychology 2008;41:171–181.
49. Meyers DC, Katz J, Chien V, Wandersman A, Scaccia JP, Wright A. Practical Implementation Science: Development of the Quality Implementation Tool. Am J Community Pscyhol 2012;50:481-96.
50. Dobbins M, Ciliska D, Cockerill R, Barnsley J, DiCenso A. A framework for the dissemination and utilization of research for health-care policy and practice. The Online Journal of Knowledge Synthesis for Nursing 2002; 9(7).
51. Venkatesh V, Morris MG, Davis GB, Davis FD. User acceptance of information technology: Toward a unified view. MIS Quart 2003;27(3):425-478.
52. Yarbrough AK, Smith TB. Technology Acceptance among physicians – A new take on TAM. Med Care Res Rev 2007;64(6):650-672.
53. Flottorp SA, Oxman AD, Krause J, Musila NR, Wensing M, Godycki-Cwirko M, Baker R, Eccles MP. A checklist for identifying determinants of practice: a systematic review and synthesis of frameworks and taxonomies of factors that prevent or enable improvements in healthcare professional practice. Implementation Science. 2013 Mar 23;8:35. doi: 10.1186/1748-5908-8-35.
54. Proctor E, Silmere H, Raghavan R, Hovmand P, Aarons G, Bunger A, Griffey R, Hensley M. Outcomes for Implementation Research: Conceptual Distinctions, Measurement Challenges, and Research Agenda. Adm Policy Ment Health. 2011 Mar; 38(2): 65–76.
55. Praveen D, Patel A, Raghu A, Clifford GD, Maulik PK, Abdul AM, et al. Development and field evaluation of a mobile clinical decision support system for cardiovascular diseases in rural India. JMIR mHealth uHealth. 2014;2:e54.
56. McEvoy R, Ballini L, Maltoni S, O’Donnell CA, Mair FS, MacFarlane A. A qualitative systematic review of studies using the normalization process theory to research implementation processes. Implement Sci. 2014;9:2.
57. Glasgow RE, Vogt TM, Boles SM. Evaluating the public health impact of health promotion interventions: the RE-AIM framework. American Journal of Public Health. 1999 Sep;89(9):1322-7.
58. Phillips CJ, Marshall AP, Chaves NJ, Lin IB, Loy CT, Rees G, et al. Experiences of using Theoretical Domains Framework across diverse clinical environments: a qualitative study. J Multidiscip Healthc. 2015;8:139–46.
59. Fleming A, Bradley C, Cullinan S, Byrne S. Antibiotic prescribing in long-term care facilities: a qualitative, multidisciplinary investigation. BMJ Open. 2014;4(11):e006442.
60. Möhler R, Bartoszek G, Kopke S, Meyer G. Proposed criteria for reporting the development and evaluation of complex interventions in healthcare (CReDECI): guideline development. International Journal of Nursing Studies 49 (2012) 40–46
61. Wong G, Westhorp G, Manzano A, Greenhalgh J, Jagosh J, Greenhalgh T. RAMESES II reporting standards for realist evaluations. BMC Medicine 2016;14:96.
62. Hoffman TC, Glasziou PP, Boutron I, Milne R, Perera R, Moher D, Altman DG, Barbour V, Macdonald H, Johston M, Lamb SE, Dixon-Woods M, McCulloch P, Wyatt JC, Cha A, Michie S. Better reporting of interventions: template for intervention description and replication (TIDieR) checklist and guide. BMJ 2014;348:g1687.
63. Albrecht L, Archibald D, Scott SD. Development of a checklist to assess the quality of reporting of knowledge translation interventions using the Workgroup for Intervention Development and Evaluation Research (WIDER) recommendations. Implementation Science 2013;8:52.

**Appendix H**

Details of the impact driver domains and their sub-domains of the framework for Knowledge-Activated Tools (KaT): SUSTAINABILITY, SCALABILITY, INTEGRATED KT

| **SUSTAINABILITY:** Sustainability is defined as: *“The degree to which an innovation continues to be used after initial efforts to secure adoption is completed”*^122^. In the conceptual KaT framework, sustainability is represented as a ring encircling the three broad domains (develop, implement, disseminate) to emphasize that assessment of the sustainability potential of KT tools and products should be considered early on, and across any or all of KT tool development, implementation and/or dissemination. Sustainability has a close relationship with, and is an important implementation outcome. | | | | |  |
| --- | --- | --- | --- | --- | --- |
| **Sub-domain** | | | **Purpose/Description** | |  |
| 1. **Engage stakeholders throughout all steps of sustainability assessment** | | | Engage with iKT team throughout all steps of sustainability assessment. | |  |
| 1. **Identify the purpose of sustainability** | | | Identify the purpose of sustainability, which may involve the continuation of the tool as originally designed or the need to adapt it in other contexts which may be very different from the one in which it was originally developed or tested^110^. Determine when sustainability will be assessed. The sustainability potential of the KT tool or product should be assessed early during the tool development process (i.e., between development and implementation) and at the time of implementation to identify any anticipated challenges and mitigating strategies to sustainability^3;111^. | |  |
| 1. **Develop a sustainability plan** | | | Develop a sustainability plan; assess sustainability over several years rather than at a single time to capture any variations that might occur over time be^110^ | |  |
|  | 1. Assess the context and characteristics of the adopter environment | | Assess both the outer (policies, legislation) and inner (organizational characteristics (e.g., structure, climate, culture, resources). This also includes understanding the learning and problem-solving skills needed by key individuals and organizations to respond to challenges related to changing environments^110-112^. Use the experience and knowledge of the context that was learned during the implementation phase^112^. | |  |
|  | 1. Identify the determinants of sustainability | | Identify the determinants of sustainability, and understand if the factors influencing sustainability differ from those influencing implementation^110;112^. | |  |
|  | 1. Assess the fit and effectiveness of the KT tool | | Assess the fit, and effectiveness of the KT tool or product^110^ | |  |
|  | 1. Assess fidelity and adaptation of the KT tool | | Assess adaptation (whether the tool is adapted or changes over time) and fidelity (a combination of adherence to a prescribed set of practices at the right dose or intensity, competence in delivery, and differentiation from other tools; and efforts to align the intervention and the setting^110;113^. It is important to understand when and to what components of the tool fidelity is necessary^110;112;113^. | |  |
|  | 1. Assess the capacity to sustain the KT tool | | Funding, resources, workforce characteristics and stability, interpersonal processes. These include funding sources for conducting the sustainability research^110;112^ | |  |
|  | 1. Adapt learnings from implementation | | Use the experience and knowledge of the context that was learned during the implementation phase^112^. | |  |
| 1. **Monitor and evaluate sustainability** | | | Monitor and evaluate sustainability of the KT tool or product | |  |
|  | 1. Define sustainability | | Consider factors in choosing a definition to guide research on the sustainment of the tool including clearly specification of research questions regarding each factor. These factors are: (1) whether, and to what extent, the core elements (the elements most closely associated with desired health benefits) are maintained; (2) the extent to which desired health benefits are maintained or improved upon over time after initial funding or supports have been withdrawn; (3) the extent, nature, and impact of modifications to the core and adaptable/peripheral elements of the program or innovation; and (4) continued capacity to function at the required level to maintain the desired benefits. A program or intervention’s impact may be considered sustained if desired health benefits remain at or above the level achieved during implementation and this increase can be attributed to continuation of the program. A program or intervention may be considered to be sustained at a given point in time if, after initial implementation support has been withdrawn, core elements are maintained (e.g., remain recognizable or delivered at a sufficient level of fidelity or intensity to yield desired health outcomes) and adequate capacity for continuation of these elements is maintained^110^. | |  |
|  | 1. Determine the focus of the sustainability assessment | | Determine the focus of the sustainability assessment, which could be on the KT tool or product rather than the system in which it is introduced. In this respect, there will be a set of factors or conditions that will increase the likelihood of sustainability^114^. This perspective puts an emphasis on the determinants of the “preservation, fidelity to, or discontinuation of a program”^110^. Examination of sustainability from an ecological or complex-systems perspective, to understand the interconnection between broader environmental forces, contextual influences, and the KT tool^115-116^. Research conducted from an ecological perspective seeks understanding of how the intervention and the local context mutually adapt and evolve and how this process impacts sustainability^117^. | |  |
|  | 1. Define outcomes of desired benefits | | The desired impact and benefits of the program or intervention should be identified. Stakeholder goals for sustainability should also be considered in the interpretation of findings (Must the program be sustained at the same level, or improved upon? To what extent is a lower level of implementation fidelity or a partially sustained program consistent with stakeholders’ goals for the project? At what point, and under what circumstances, is discontinuation, modification, or implementation of a more effective, efficient, or better-fitting intervention advisable?) should be considered in the interpretation of findings^110^. | |  |
|  | 1. Consider using a sustainability framework | | Consider using a sustainability framework to evaluate and measure sustainability of the KT tool or product. | |  |
|  |  | **DSF Framework**  *Dynamic Sustainability Framework* | | DSF (Dynamic Sustainability Framework) emphasizes that change exists in the use of interventions over time, the characteristics of practice settings, and the broader system that establishes the context for how care is delivered^111^. The model comprises of the intervention, the context in which the intervention is delivered, and the broader ecological system within which the practice settings exist and operate. DSF emphasizes the idea that change is constant, and that there needs to be a continuous process for optimizing the “fit between the intervention and a dynamic delivery context to achieve maximal benefit”^111^. The DSF consists of seven tenets: *“1) An intervention should not be optimized prior to implementation, or even prior to ‘sustainability phase’ onset; 2) Interventions can be continually improved, boosting sustainment in practice, and can enable ongoing learning among developers, interventionists, researchers and patients; 3) Ongoing feedback on interventions should use practical, relevant measures of progress and relevance; 4) Voltage drop is NOT inevitable; 5) Programs should be more likely to be maintained when there is strong ‘fit’ between the program and the implementation setting; 6) Organizational learning should be a core value of the implementation setting; 7) Ongoing stakeholder involvement throughout should lead to better sustainability”* ^111^. | |
|  |  | **Framework of public health program capacity for sustainability** | | The framework presents nine domains specific to public health interventions that are essential for success. It can be used to assess a program’s capacity for sustainability for public health decision makers, program managers, program evaluators, and dissemination and implementation researchers to consider when developing and implementing prevention and intervention programs: 1) Political Support; 2) Funding Stability; 3) Partnerships; 4) Organizational Capacity; 5) Program Evaluation; 6), Program Adaptation; 7) Communications; 8) Public Health Impacts, and 9) Strategic Planning^113;118^ . | |
|  |  | **NHS Sustainability model** | | The NHS Sustainability model consists of 10 factors that may improve sustainability, and can be used by teams who are implementing new practice in their organization to identify strengths and weaknesses of the implementation plan of their innovation, and to predict the likelihood of sustainability^119^. The sustainability guide offers practical advice on achieving sustained use of the initiative by providing the means for teams to address identified challenges and prompt discussion and action to address them, particularly across factors with maximum potential for improvement (i.e., largest difference between identified score and maximum potential sustainability score). The process is aimed at raising early awareness of sustainability challenges, and the opportunity for teams to iteratively address these challenges to optimize the new initiative’s potential for impact^120^. The model can be used in the planning and early stages of implementation to evaluate the likelihood that an innovation will be sustained, and it can also be used at any phase of a project^110^ | |
|  |  | **PRISM model** | | The Practical, Robust Implementation and Sustainability Model (PRISM) model uses concepts from the quality improvement, chronic care, and the diffusion of innovations along with measures of population-based effectiveness interventions. It includes key elements to consider when implementing evidence-based practices and key questions to enhance implementation and sustainability for each domain. The domains include: Program (Intervention) – organizational and population perspectives of the intervention; External environment; Implementation and sustainability infrastructure; Recipients (organizational and population characteristics)^77^. | |
|  |  | **Program Sustainability Index** | | A 29-item program sustainability index to assess six factors related to the sustainability of community-based programs^121^. | |

1. Straus SE, Tetroe J, Graham ID. Knowledge translation in health care: Moving from evidence to practice. 2nd ed. West Sussex, UK: Wiley; 2013.
2. Feldstein, A. C. & amp; Glasgow, R. E. (2008). A Practical, Robust Implementation and Sustainability Model (PRISM) for integrating research findings into practice. The Joint Commission Journal on Quality and Patient Safety, 34(4), 228-243.
3. Wiltsey Stirman S, Kimberly J, Cook N, Calloway A, Castro F, Charns M. The Sustainability of new programs and innovations: a review of the empirical literature and recommendations for future research. Implementation Science 2012;7:17]
4. Chambers DA, Glasgow RE and Stange KC. The dynamic sustainability framework: addressing the paradox of sustainment amid ongoing change. Implementation Science 2013;8:117
5. Proctor 2015 Racine DP: Reliable effectiveness: a theory on sustaining and replicating worthwhile innovations. Adm Policy Ment Health 2006, 33(3):356-387.
6. Tricco AC, Ashoor HM, Cardoso R, MacDonald H, Cogo E, Kastner M, Perrier L, McKibbon A, Grimshaw JM, Straus SE. Sustainabiity of knowledge translation interventions in healthcare decision-making: a scoping review. Implementation Science 2016;11:55
7. Racine DP: Reliable effectiveness: a theory on sustaining and replicating worthwhile innovations. Adm Policy Ment Health 2006, 33(3):356-387.
8. Gruen RL, et al: Sustainability science: an integrated approach for health programme planning. Lancet 2008, 372(9649):1579.
9. Aarons G, Hurlburt M, Horwitz S: Advancing a Conceptual Model of Evidence-Based Practice Implementation in Public Service Sectors. Adm Policy Ment Health Ment Health Serv Res 2011, 38(1):4-23
10. Kirsh SR, Lawrence RH, Aron DC: Tailoring an intervention to the context and system redesign related to the intervention: A case study of implementing shared medical appointments for diabetes. Implement Sci 2008, 3(1):34
11. Schell SF, Luke DA, Schooley MW, Elliott MB, Herbers SH, Mueller NB, et al. Public health program capacity for sustainability: a new framework. Implement Sci. 2013;8:15.
12. Maher L, Gustafson D, Evans A. NHS Sustainability Model. NHS Institute for Innovation and Improvement; 2010. Available at: [www.institute.nhs.uk/sustainability](http://www.institute.nhs.uk/sustainability).
13. Doyle C, Howe C, Woodcock T, Myron R, Phekoo K, McNicholas C, et al. Making change last: applying the NHS institute for innovation and improvement sustainability model to healthcare improvement. Implement Sci. 2013;8:127.
14. Mancini JA, Marek LI: Sustaining community-based programs for families: Conceptualization and measurement. Fam Relat 2004, 53(4):339-347.
15. Rogers EM. Diffusion of Innovations. Fourth ed. New York (NY): The Free Press, 1995.

| **SCALABILITY** Scalability is defined as the *“deliberate efforts to increase the impact of health service innovations successfully tested in pilot or experimental project so as to benefit more people”*^123^. In the conceptual KaT framework, scalability is represented as a ring encircling the three broad domains (develop, implement, disseminate) to emphasize that assessment of the scalability potential of KT tools and products should be considered early on, and across any or all of KT tool development, implementation and/or dissemination. Scalability should be considered along with Sustainability. | | | | |
| --- | --- | --- | --- | --- |
| **Sub-domain** | | | | **Purpose/Description** |
| 1. **Identify scale-up objectives and scope** | | | | Identify scale-up objectives and scope |
| 1. **Identify scale-up team** | | | | Identify the stakeholders who will plan and execute the scale-up plan^100;122,123^ |
|  | 1. Define the scalability capacity of the resource team that developed and tested the successful KT tool | | | A resource team are people who were involved in the development and testing of the tool and are seeking to promote its wider use. They may be formally charged with promoting the innovation or may act informally in this role. Resource teams are more likely to be successful if they possess the following features: effective and motivated leaders who command authority and have credibility with the adopter organization; a unifying vision; an appreciation of the adopter organization’s capacities and limitations; an understanding of the political, social and cultural environments within which scaling up takes place; the ability to generate financial and technical resources; relevant technical skills; training capacity; and management skills^122,123^ |
|  | 1. Identify target adopters of the user organization | | | The system, organization or people who will adopt the tool or product. Successful transfer of innovations is facilitated when the user organization has the following characteristics: the members of the user organization perceive a need for the innovation; the user organization has the appropriate implementation capacity; the timing and circumstances are right; the user organization possesses effective leadership and internal advocacy; the resource and user organizations are similar in characteristics, and are in close physical proximity^100;122;124^ |
|  | 1. Identify adopter site champions | | | Those who will facilitate the adoption of the tool in the adopter setting. |
| 1. **Ensure that the KT tool is ready for scale-up** | | | | Attributes of the KT tool that facilitate innovation transfer and utilization include: must be based on sound evidence or supported by respected or credible persons or institutions; observable to ensure that potential users can see the results; relevant for addressing persistent or “sharply felt” problems and challenges; have a relative advantage over existing practices; easy to install and understand; compatible with the potential users’ established values, norms and facilities; testable without committing the potential user to complete adoption^122;124^ |
| 1. **Develop a scale-up plan** | | | | Develop a scale-up plan with the identified team to addresses objective and scope. Consider using the nine-step scale-up strategy to develop the scale-up plan as outlined by the World Health Organization (WHO)^123^ |
|  | | 1. Assess and understand the context of the adopter organization or setting | | Assess the social, cultural, political and economic context within which scaling up takes place. “The environment can include both the people and communities who require health services as well as the multiple actors, sectors and influences that shape the process of bringing successful interventions to scale. These include the policy setting, the political, system, bureaucratic culture, the health sector, the socioeconomic and cultural contexts and the influence of global trends.”^100;122,123^ |
|  |  | 1. Identify the determinants of scale-up | | Identify the facilitators and challenges to scaling up the KT tool using formalized qualitative and quantitative methods. |
|  |  | 1. Consider factors that will maximize the potential for successful scale-up | | Factors that will maximize the potential for successful scale-up include: clear messages; personal contact and informal communication; early involvement of members of the user organization; adaptation of the innovation to the local context; participatory approaches; technical assistance and a supportive approach; sufficient time to implement new approaches; strong diffusion channels; training support to ensure skills transfer; systematic use of evidence on the process and outcomes of scaling up; ongoing focus on sustainability^122;125^. Innovation adopters also need to: “develop and enhance demand, and align any available incentives and expectations; “communicate in ways that promote awareness, support accurate assessment of costs and benefits, and convey an understanding of the requirements”; “provide technical assistance, tools, and resources, including guidance for adaptation and for management of the organizational context, and recognize and address heterogeneity across settings with regard to needs, circumstances, capabilities, and other factors^126^ |
|  |  | 1. Assess the type of scale-up strategy that best fits with objectives and identified challenges | | The plans and actions necessary to fully establish an innovation in policies, programmes and service delivery”^123^. Strategies may include decisions about what specifically will be scaled-up, what type of scaling up will be appropriate; how it will be organized; how fast it will be done; how various environmental challenges and opportunities will be engaged; and what role research will play in the process^122,123^. The different types of scaling up do not function in isolation. Spontaneous diffusion may occur in combination with scaling up that is guided, and expansion is typically most effective when supported by political and institutional scaling up^122,123^. |
|  |  |  | Horizontal scaling up | Replication or expansion scaling up occurs when innovations are replicated in different locations or are expanded to serve larger populations or different categories of beneficiaries |
|  |  |  | Vertical scaling up | Political, policy or legal initiative scaling up takes place when innovations are institutionalized through policy or legal action. |
|  |  |  | Diversification | Diversification (i.e., functional scaling up or grafting), consists of testing and adding new interventions to existing innovations |
|  |  | 1. Assess costs and mobilizing resources | | Financial and human resources involved in formulating a scaling-up strategy whether it’s to expand an innovation to a new geographical sties or population, time needed to obtain political support, any additional testing and implementation of new components of the tool, and for evaluating and working with spontaneous scaling up that may occur; and to have supportive, financial, organizational, governance, and regulatory structures^3;62;122;123;125^. |
| 1. **Monitor and evaluate** | | | | Special research and evaluation procedures should monitor whether the innovation is being implemented as expected and the extent to which local adaptation maintains minimum established standards^122,123^. “Plans and resources for further expansion can be adjusted when problems and dysfunctions are encountered. At the same time, identifying the positive features and results obtained from scaling up motivates communities, providers, decision-makers and managers by demonstrating the value of new approaches”^122,123^. Assessments may include service statistics, studies, local assessments, environmental analysis^123^. |

1. Straus SE, Tetroe J, Graham ID. Knowledge translation in health care: Moving from evidence to practice. 2nd ed. West Sussex, UK: Wiley; 2013.
2. Glasgow RE, Vinson C, Chambers D, Khoury MJ, Kaplan RM, Hunder C. National Institutes of Health approaches to dissemination and implementation sciences: Current and future directions. AJPH 2012;102(7):1274-81.
3. Massoud MR, Donohue KL, and McCannon CJ. 2010. Options for Large‐scale Spread of Simple, High impact Interventions. Technical Report. Published by the USAID.
4. Simmons R, Shiffman J: Scaling Up Health Service Innovations: A Framework for Action. In Scaling Up Health Service Delivery: From Pilot Innovations to Policies and Programmes. Geneva, Switzerland: World Health Organization; 2007:1–30.
5. WHO: Nine steps for developing a scaling up strategy 2010. Available at: <http://www.who.int/immunization/hpv/deliver/nine_steps_for_developing_a_scalingup_strategy_who_2010.pdf>.
6. Glaser EM, Abelson HH, Garrison KN. Putting knowledge to use: facilitating the diffusion of knowledge and the implementation of planned change. San Francisco, CA, Jossey-Bass Inc., 1983.
7. Hanson K, Ranson MK, Oliveira-Cruz V, Mills A. Expanding access to priority health interventions: a framework for understanding the constraints to scaling up. Journl of Knowledge Management 2003;15:1-14.
8. Mittman B. AHRQ 2014: Factors that influence the scale-up and spread of innovations. Available at: <https://innovations.ahrq.gov/perspectives/factors-influence-scale-and-spread-innovations>.

| **INTEGRATED KT (IKT)** is defined as “*an ongoing relationship between researchers and decision makers (clinicians, managers, policy-makers, etc.) for the purpose of engaging in a mutually beneficial research project or program of research to support decision making*”^110^. Also known as collaborative research or participatory action research, iKT is a way of doing research that applies KT principles to the entire research process. To ensure that research results will be more relevant and useful to knowledge users (individuals who are interested in or will be able to use the knowledge for decision making), iKT facilitates the collaboration between researchers and knowledge users as partners in the entire research process from the development or refinement of research questions, selection of the methodology and outcome measures, data collection and tools development through to the interpretation of findings, crafting the messages, and dissemination of the results^1^. In the conceptual KaT framework, iKT is represented as a ring encircling the three broad domains (develop, implement, disseminate) to emphasize the importance of applying an iKT approach early, and to continue through to the development, implementation and/or dissemination of KT tools and products. | | | |
| --- | --- | --- | --- |
| **Sub-domain** | | | **Purpose/Description** |
| 1. **Engage relevant stakeholders and establish partnerships throughout all the steps to:** | | | Convene relevant stakeholders, partners and knowledge users (building on individuals identified in the Discovery stage) to plan the KT tool development, implementation, dissemination^2,3^. |
|  | 1. Identify knowledge users and partners | | Clearly define and identify knowledge users and partners. Facilitators include identifying partners with pre-established links to ease and expedite interaction^101-102^. Ensure that decision makers are considered as part of the iKT team (if appropriate) as evidence suggests that they are not always engaged as partners^101^. |
|  | 1. Identify and clarify expectations about iKT member roles | | Identify and clarify expectations of iKT team member roles, scope and contribution to build trust and avoid confusion^101-102^. |
|  | 1. Develop the iKT organizational and governance structure | | Identify the objectives of iKT and the different needs for iKT that will be needed at different parts of the work in the context of the purpose and context that was identified in the Discovery stage. This also includes identifying the organizational structure of iKT (i.e., steering groups, working groups, advisory groups, etc) |
|  | 1. Determine optimized communication mechanism between iKT members | | Define strategies that will be used to establish optimized communication amongst the team, and to enable efficient modes of communication and exchange of information to enable a shared understanding^3;101-103^. |
|  | 1. Identify any anticipated challenges | | Identify any anticipated challenges to ensuring that all stakeholders and knowledge users are involved throughout all the stages of creating the KT tool, and mitigating strategies to implementation |
| 1. **Develop an iKT plan** | | | Develop an iKT plan matched to overall purpose and scope (from Discovery stage) including the methods that will be used |
|  | 1. Convene teams (core iKT team to steer; advisory groups; working groups) | | Convene a core iKT team that will steer the project across all domains (develop, implement, disseminate) as well as for sustainability and scalability aspects. Convene advisory and working groups as needed for each stage of the projects and across domains (develop, implement, disseminate) |
|  | 1. Continuously monitor and evaluate communication mechanism | | Monitor and evaluate communication mechanism continuously; identify the indicators that will be measured; identify who will monitor and evaluate the iKT and how |
|  | 1. Establish a style of collaboration that provides opportunities for co-production of KT | | Establish a close style of collaboration that provides opportunities to overcome barriers to achieving and co-producing KT^104^. Use a phased approach to develop a shared language^101^. |
|  | 1. Capture and report iKT activities | | Capture and report “iKT activities (e.g., brainstorming sessions, data interpretation sessions, passive dissemination through websites), who is involved in which activity, sho is leading the activity and how often activities take place”^101^. |
| 1. **Monitor and Evaluate the iKT plan that was implemented** | | | Evaluate the effectiveness of the iKT structure that was designed overall and for individual stages of the project (i.e., across develop, implement, disseminate) and for sustainability and scalability considerations. Share these findings with iKT team by outlining what worked and didn’t work and why. |
|  | 1. Identify the goal of iKT monitoring and evaluation | | Identify the goal of iKT monitoring and evaluation |
|  | 1. Jointly assess progress | | Jointly assess progress and implement changes as needed^101-102^. |
|  | 1. Consider using an iKT framework or theory to guide its process | | Consider using an existing iKT framework to guide the planning and execution of the iKT plan^1;3;101^ |
|  |  | **Collaborative Model for KT**  *Collaborative engagement in clinical settings* | The Collaborative Model for KT is a conceptualization of KT as “a dialogic, collaborative engagement between researchers and practitioners through which people come to reflect on what they do, and its consequences, and identify what they might do differently by drawing on research based knowledge”^105^. The approach uses the concepts of process (translation) and content (knowledge). The process dimension has two main components: (a) a collaborative relationship between researchers and practitioners, and (b) the knowledge translation cycle that embodies its dynamic nature. The process dimension is embedded within the content dimension, which involves an ongoing cycle of data collection/analysis/and synthesis of knowledge^105^. |
|  |  | **CollaboraKTion Framework for Community Based Knowledge Translation**  *To collaboratively identify and create changes to contextual factors that influence health outcomes* | The CollaboraKTion framework states that community-based KT is an iterative process comprising five overarching processes: (1) contacting and connecting; (2) deepening understandings; (3) adapting and applying the knowledge base; (4) supporting and evaluating continued action; and (5) transitioning and embedding as well as several key elements within each of these processes (e.g. building on existing knowledge, establishing partnerships)^106^. |
|  |  | **Co-KT Framework**  *To conduct population focused KT* | Co-KT is a collaborative approach to conducting KT informed by a participatory theoretical framework. It is defined as: “a framework for actioning the intent of researchers and communities to co-create, refine, implement and evaluate the impact of new knowledge that is sensitive to the context (values, norms, and tacit knowledge) where it is generated and used”^107^. It is meant to guide the conducti of KT within a population health study. The framework involves five steps embedded within an overarching context (i.e., the ‘study context’ [i.e. the study site or location, stakeholders, local information and expertise] and the ‘research context’ [i.e. researchers who facilitate the study and ensure scientific integrity throughout the co-KT process])^107^ |
|  |  | **iKT Capacity Framework**  *To assess readiness and capacity for iKT* | iKT Capacity Framework has nine components across 3 domains (organizational, professional, individual). It can be used by organizations to plan, or assess their capacity for IKT at the organizational, professional and individual levels^108^. |
|  | 1. **Consider using a reporting checklist to design iKT initiatives** | | Consider using a reporting checklist to design iKT initiatives or to report evaluation findings^101^ |
|  |  | **WIDER checklist** | The WIDER (Workgroup for Intervention Development and Evaluation Research recommendations in systematic reviews) checklist recommends describing: the intervention (approaches, strategies), mode of delivery (intensity, duration, timing), intervention content (knowledge generated or shared), participants and their role (the characteristics of those sponsoring, delivering, and receiving the intervention), setting, and adherence or fidelity^94;109^. |

1. Canadian Institutes of Health Research (CIHR): Guide to Knowledge Translation Planning at CIHR: Integrated and End-of-Grant Approaches; 2012. Available at: <http://www.cihr-irsc.gc.ca/e/45321.html>.
2. Straus SE, Tetroe J, Graham ID. Knowledge translation in health care: Moving from evidence to practice. 2nd ed. West Sussex, UK: Wiley; 2013.
3. Albrecht L, Archibald D, Scott SD. Development of a checklist to assess the quality of reporting of knowledge translation interventions using the Workgroup for Intervention Development and Evaluation Research (WIDER) recommendations. Implementation Science 2013;8:52.
4. Gagliardi AR, Berta W, Kothari A, Boyko J, Urquhart R. Integrated knowledge translation (IKT) in health care: a scoping review. Implementation Science 2016;11:38.
5. Hofmeyer A, Scott C, Lagendyk L. Researcher-decision-maker partnerships in health services research: practical challenges, guiding principles. Health Serv Res. 2012;12:280
6. Manojlovich M, Squires JE, Davies B, Graham ID. Hiding in plain sight: communication theory in implementation science. Implementation Sicence 2015;10:58.
7. Heaton J, Day J, Britten N. Collaborative research and the co-production of knowledge for practice: an illustrative case study. Implementation Science 2016;11:20.
8. Baumbusch JL, Kirkham SR, Khan KB, McDonald H, Semeniuk P, Tan E, et al. Pursuing common agendas: a collaborative model for knowledge translation between research and practice in clinical settings. Res Nurs Health 2008;31(2):130-140.
9. Jenkins EK, Kothari A, Bungay V, Johnson JL, Oliffe JL. Strengthening population health interventions: developing the CollaboraKTion Framework for Community-Based Knowledge Translation. Health Research Policy and Systems 2016;14:65.
10. Kitson A, Powell K, Hoon E, Newbury J, Wilson A, Beilby J. Knowledge translation within a population health study: how do you do it? Implement Sci. 2013;8:54.
11. Gagliardi AR, Dobrow MJ. Identifying the conditions needed for integrated knowledge translation (IKT) in health care organizations: qualitative interviews with researchers and research users: BMC Health Services Research 2016;16:256.
12. Camden C, Shikako-Thomas K, Nguyen T, Graham E, Thomas A, Sprung J, et al. Engaging stakeholders in rehabilitation research: a scoping review of strategies used in partnerships and evaluation of impacts. Disabil Rehabil. 2015;37:1390–400.
13. Kothari A, Wathen CN. A critical second look at integrated knowledge translation. Health Policy. 2013;109(2):187–91.

**Appendix I**

Details of the ACTION PLAN domain and its sub-domain of the framework for Knowledge-Activated Tools (KaT)

| **ACTION PLAN** The Action Plan is the final output of the KaT framework, which is customized according to the needs, purpose, scope and context of the tool user. The Action Plan will contain a summary of the Discovery stage outputs, and recommendations for action for each domain of the framework that is relevant to identified purpose and scope (whether it’s any or all of develop, implement, disseminate) including how each of iKT, Evaluation, Sustainability, and Scalability fit within the overall customized plan. The Action Plan will also include learning supports (i.e., templates relevant to Action Plan items, and Instructions on how to use all of these). | | |
| --- | --- | --- |
| **Sub-domain** | | **Purpose/Description** |
| 1. **Summary of outputs from Discovery** | | Inputs from the Discovery stage will generate a series of outputs that can be used to inform a recommended pathway of next steps within the KaT framework.. |
|  | 1. Clearly defined gap(s), purpose and research questions | Users will have clearly defined their gap(s), purpose and research questions |
|  | 1. Defined scope (identified stakeholders, context and resources) | Users will have defined their scope in terms of their stakeholders (the knowledge user(s) and partners who should be involved in addressing the problem); the context or setting in which the KT tool will be delivered; and the resources that are needed vs. what is available and feasible to achieve outcomes |
|  | 1. List of potential KT tools and products that may be considered to address identified purpose and scope. | Users of the KaT framework will be guided through a process to identify the existing knowledge base on their problem, to identify KT tools and products that may be considered to address them, and whether there is a need to develop a new KT tool or to adapt an existing one. In addition, knowledge users will be able to identify the type of tool(s) that may best match their purpose and scope. To facilitate this, we have developed a table of existing KT tools and products that are organized by target audience (patients, providers or both, researchers, hospitals and managers, and health system), and each tool is mapped to seven broad purpose categories. You can view this table through the Delphi survey. |
|  | 1. Learning supports (templates, instructions) | Templates for each of the 3 subdomains of discovery (identify KT purpose, define scope, search the evidence); Instructions manual |
| 1. **Action Plan according to the needs, purpose, scope and context of the user** | | An Action Plan will be generated overall, which will include a customized output based on identified purpose and context, and include individual plans for any or all of the three domains of the framework (develop, implement, disseminate). The plan will also include learning supports (i.e., templates and instructions) |
|  | 1. Development plan | A detailed plan to develop or adapt the KT tool or product to guide and support knowledge users to engage relevant stakeholders; to identify the existing evidence base on the chosen KT tool/product; to select a theoretical basis for the development of adaptation of the KT tool/product; to develop or adapt a functioning prototype using a user-entered design; and conduct usability evaluation of the KT tool/product. |
|  | 1. Implementation plan | A detailed implementation plan to guide and support knowledge users to engage with relevant stakeholders to determine their objectives for implementation, define roles and optimized communication mechanisms; to identify the implemetability of the KT tool/product; to develop and execute the implementation plan; monitor and evaluate knowledge use; and to organize and document findings using appropriate reporting criteria. |
|  | 1. Dissemination plan | A detailed dissemination plan that is appropriate to the strength and scope of the research results, clearly defines the expertise of the team; considers all relevant knowledge users, the context and available resources; and helps identify the determinants of dissemination. |
| 1. **A description of how iKT, Evaluation, Sustainability, and Scalability fit within the Action Plan** | | The Action Plan will include a section to describe how each of iKT, Evaluation, Sustainability, and Scalability fit within the overall customized plan |
| 1. **Suggested timelines (overall and for each section of the Action Plan)** | | Suggested timelines will be provided for each relevant domain, and section(s) of the plan (i.e., iKT, Evaluation, Sustainability, Scalability) |
| 1. **References and links to sources of action recommendations outlined in the Action Plan** | | All recommended action plan items will be referenced, and links to sources provided. |
| 1. **Templates relevant to Action Plan items** | | Templates (online and paper-based) will be provided for each section of the Action Plan to facilitate its implementation |
| 1. **Instructions manual on how to use the Action Plan and Templates** | | Instructions manual will be provided on how to use the Action Plan and templates |

**Appendix J**

**Delphi study:** Items of the Knowledge-activated Tools (KaT) framework that did not reach consensus by the Round 1 panel including qualitative comments and suggestions about each domain*

| **KaT Framework Domain** | **Domain factor:** *Comments* | | **N** | **Mean (SD)** | **Median** | **IQR†** | **Percent agreement to include‡** |
| --- | --- | --- | --- | --- | --- | --- | --- |
| **DISCOVER** | DISCOVER is an appropriate label | | 35 | 5.0 (1.40) | 5.0 | 2 | 71% |
|  |  | Alternative suggestions for domain label: *Explore (n = 4); Identify KT purpose (n = 1); Assess (n = 1); Needs assessment (n = 1); Preparation phase (n = 1).* | | | | | |
| **DEVELOP** | Knowledge users will find it useful. | | 35 | 5.7 (1.53) | 6.0 | 2 | 74% |
|  |  | - *This is excellent and thorough work, but it may not be practical, feasible, or usable for most knowledge users, especially because of the time needed to go into this level of detail and consideration).* - *This will only work if knowledge users and researchers work together.* - *Not all knowledge users may find the Develop domain useful as they may have already developed tools* | | | | | |
| **DISSEMINATE** | Knowledge users will find it useful. | | 35 | 5.6 (1.50) | 6.0 | 3 | 71% |
|  |  | - *Seems too complicated. Users may feel overwhelmed"* - *Depending on scope, "Disseminate" may overlap with "Implement." I would prefer seeing these two components more integrated"* - *Users not familiar with KT terms may find it difficult to differentiate between the terms (e.g. disseminate v. implement)* | | | | | |
| **IMPACT DRIVERS** | The order in which the four impact drivers are represented *make sense* | | 35 | 5.2 (1.63) | 6.0 | 2 | 71% |
|  | The placement of the four impact drivers clearly illustrates that they should be considered across each of the three broad domains of the framework (i.e., develop, disseminate, implement) | | 35 | 5.4 (1.58) | 6.0 | 2 | 74% |
|  |  | - *Does one read from the centre of the circle to the outside, or from the outside in?* - *Not sure what the order presents. Is it suggesting that Scalability is the most important, or that IKT is?* - *Visual presentation is confusing: Impact drivers' names are repeated three times. Why? Seems redundant.* - *Cannot understand visual at a glance.* - *Evaluation is included as an impact driver, but users may interpret the "develop -> disseminate -> implement" layout to imply that there is no need to test/evaluate* - *Should Evaluation be included as a fourth component in the circle rather than just an impact driver?* | | | | | |
| **CORE** | The placement of the CORE clearly illustrates that a KT tool is the ultimate goal and end product resulting from using the KaT framework | | 35 | 5.7 (1.62) | 6.0 | 2 | 69% |
|  |  | - *What does the core represent? It’s placed in the centre, but "action plan" may be considered the end product. Users may be confused whether the ultimate goal is to create a tool or an action plan* - *Is the tool the core or ultimate goal of the KaT Framework? or is the goal of the framework practice change, or use of the KaT Framework?* - *As presented, the core feels like the starting point* | | | | | |
| **PLANNING** | PLANNING, which encircles the core, is important to include as part of the overall KaT framework | | 35 | 5.2 (1.61) | 6.0 | 3 | 60% |
|  | The placement of PLANNING clearly illustrates that a plan can be generated for each or all of the three broad domains of the KaT framework (i.e., develop, disseminate, implement) | | 35 | 4.9 (1.72) | 5.0 | 3 | 54% |
|  |  | - *Doesn’t add much value because it is implicit that some planning needs to occur* - *Seems to suggest that the user must plan for all three broad domains. Can it be clarified that different plans can be generated depending on the domain you are focusing in?* - *As planning process is embedded throughout, the planning "ring" in the visual may be distracting/confusing because it may suggest that there is something additional users have to do at each stage.* | | | | | |
| **ACTION PLAN** | Researchers will find it useful | | 35 | 5.7 (1.33) | 6.0 | 3 | 74% |
|  | Health care providers will find it useful | | 35 | 5.8 (1.20) | 6.0 | 3 | 77% |
|  | Policy or decision makers will find it useful | | 35 | 5.7 (1.18) | 6.0 | 3 | 74% |
|  |  | - *It’s difficult to assess the usefulness of the action plan because it is unclear about what the action plan would include, and what it would do (n = 5)* - *Usefulness of the action plan depends on how it is tailored to each of these audiences (e.g. researchers, healthcare providers, policy/decision makers), and what the action plan includes and what it would do* - *An action plan may change (e.g. due to local barriers, over time), but the framework suggests a single static plan. Consider "Action Plans" instead of just "Action Plan* - *How is the action plan informed by the earlier stages?* - *Without seeing an actual example, it is hard to comment on the comprehensiveness.* - *Consider adding a template of an action plan with the identified elements* | | | | | |
| **OVERALL KaT framework** | The KaT framework is a good reflection of its intended purpose (i.e., to guide the rigorous and efficient creation of KT tools) | | 35 | 5.7 (1.30) | 6.0 | 2 | 74% |
|  | Health care providers will find it useful | | 35 | 5.3 (1.40) | 6.0 | 2 | 66% |
|  | Policy or decision makers will find it useful | | 35 | 5.3 (1.40) | 6.0 | 2 | 63% |
| **TABLE of existing KT tools** | Knowledge users will find the TABLE of existing KT tools organized by targets useful | | 35 | 5.4 (1.60) | 6.0 | 3 | 63% |
|  | Knowledge users will find the TABLE of existing KT tools mapped to purpose categories useful | | 35 | 5.4 (1.60) | 6.0 | 3 | 69% |

*SD = standard deviation; IQR = interquartile range

**†**IQR 0 = high consensus; IQR 1 = good consensus; IQR 2 = poor consensus.

‡Percent agreement to include item = score of ≥ 5 out of 7 by ≥ 80% of panel (consensus) or < 5 out of 7 by < 80% of panel (non-consensus).

**Appendix K**

**Delphi study:** Round 2 discussion points and decisions about non-consensus based items of the Knowledge-activated Tools (KaT) framework that were carried forward from Round 1 ratings

| **KaT Framework Domain** | **Domain factor from Round 1 that did not reach consensus** | **Round 2 discussions and decisions** |
| --- | --- | --- |
| **DISCOVER** | DISCOVER is an appropriate label | - DISCOVER is an overused term and too similar to the term: “discovery science” - We changed the label of this domain to EXPLORE |
| **DEVELOP** | Knowledge users will find these domains useful | - There was confusion about why we asked this question so we clarified that knowledge users would not be interacting with the KaT framework in itself but rather as a computerized version of it - Passed to Round 3 for reassessment |
| **DISSEMINATE** |  |  |
| **ACTION PLAN** |  |  |
| **3 BROAD DOMAINS** | There was some confusion about how DISSEMINATION and IMPLEMENTATION differ | - We moved the DEVELOP domain to the bottom of the framework and DISSEMINATE and IMPLEMENT on either side - We added new descriptors under each label to clarify how they are different:   - DISSEMINATE (share knowledge);   - IMPLEMENT (apply knowledge) - Passed to Round 3 for reassessment |
| **IMPACT DRIVERS** | The order in which the four impact drivers are represented *make sense* | - The panel was satisfied with the explanation of the purpose and placement of the impact drivers. - We added the word “consider” in front of the impact drivers to iterate the idea that they should be considered in the creation of KT tools and products. However, the panel thought that the addition of “consider” in front of each was unnecessary, and too wordy and cluttered, so we removed it - Passed to Round 3 for reassessment |
|  | The placement of the four impact drivers clearly illustrates that they should be considered across each of the three broad domains of the framework (i.e., develop, disseminate, implement) |  |
|  | The panel felt that the order of impact factors and their visual representation is unclear and confusing |  |
| **EVALUATE** | Some thought that EVALUATION needs to stand out more because it’s often overlooked and undervalued. Others thought that it may be confusing to include EVALUATION as an impact factor since everything is evaluated (all the domains); it’s also different from iKT, sustainability and scalability. As such, we moved EVALUATE where PLANNING used to be (i.e., encircling the core) | - The panel didn’t like EVALUATE in the area where PLANNING used to be. They suggested that since everything is evaluated, that it be represented in the outermost circle rather than embedded among the impact drivers, so we placed it in the outermost circle rather than embedded among the Impact Drivers - Passed to Round 3 for reassessment |
| **PLANNING** | PLANNING, which encircles the core, is important to include as part of the overall KaT framework | - There was consensus that the PLANNING encircling the core doesn’t add value so we removed it from the framework |
|  | The placement of PLANNING clearly illustrates that a plan can be generated for each or all of the three broad domains of the KaT framework (i.e., develop, disseminate, implement) |  |
| **CORE** | The placement of the CORE clearly illustrates that a KT tool is the ultimate goal and end product resulting from using the KaT framework | - There was confusion about what the CORE represents; and some thought that it may get confused with Action plan - Keep the CORE as it centres the framework, and will be less cluttered without the PLANNING - Passed to Round 3 for reassessment |
| **OVERALL KaT framework** | The overall framework is clear (i.e., easy to understand or interpret) | - The ratings for the overall framework reflects some of the issues that were discussed for individual domains in Round 2 - Passed to Round 3 for reassessment |
|  | The KaT framework is a good reflection of its intended purpose (i.e., to guide the rigorous and efficient creation of KT tools) |  |
| **TABLE of existing KT tools** | Knowledge users will find the TABLE of existing KT tools organized by targets useful | - Table needs more work, it needs to be reviewed for accuracy and be validated - Defer for now – needs further development and validation for the online, interactive KaT platform |
|  | Knowledge users will find the TABLE of existing KT tools mapped to purpose categories useful |  |

**Appendix L**

Survey respondents’ perceptions of the EXPLORE page of the conceptual Knowledge-activated Tools (KaT) platform and corresponding mean Likert scale score

| **EXPLORE page of the conceptual KaT platform** | | **Mean (SD)** |
| --- | --- | --- |
| It’s important to provide the opportunity for knowledge users to identify their KT purpose | | 4.79 (0.431) |
|  | - I really like the idea of developing a purpose statement upfront as well as being able to see existing tools. - Be explicit and transparent in your Explore page about why its important for users to identify their purpose. You (designers) should provide a rationale at the top of the page (maybe after the question - what is the purpose...?) that reminds users of why it is important for users to identify their purpose. For me, it helps to stay focused, but Q1 above is hard to answer because I don't know you and I are on the same page for the rationale for identifying the KT purpose. | |
| I like the idea of selecting options from a series of drop-down menus to generate a KT purpose statement | | 4.46 (0.624) |
|  | - I like the idea of drop down options because it helps the user to think through all the possibilities. - I think the use of dropdowns to provide the alternatives is fine and I like the structured composition of the KT purpose. - What it the KT tool or outcome is a combination of the options or does not fit the options. - You may need the option of selecting more than one item - especially in the outcome box, but also in some of the other boxes - How will the menus handle an "other" option? - I am wondering if you have got all the relevant constructs and options here. - Will there be "other" options for those who have elements that are not listed in the drop down menu? - With use of a drop-down menu, will the user have the option to select more than one purpose? - May need to consider to add "other" or "N/A" - I would suggest having "Other" as an option in the dropdown box at least for product/tool and user - And add an 'other' because there's no way you've captured everything here. - Dropdowns are fine as long as they allow people to select multiple options. This is sometimes tricky for users to do, so in general, I would lean toward checkboxes instead - I'd add "info" buttons for each dropdown, with some background. - As long as multiple options could be selected - I like the drop down menu however I could imagine at times I might feel somewhat limited in my options. What options for 'other' are planned? - Would the drop down menu allow for multiple choices or only one at a time (e.g., multiple stakeholders at the same time) or would one have to run each query individually as a conglomerate result may get confusing (e.g., which one is for practitioners and which one for patients) but running different queries might be more onerous | |
| Overall, I like the idea of the conceptual EXPLORE page | | 4.54 (0.557) |
|  | - Brilliant. Looks very useful - Very intuitive! The rest of the tool should be this straightforward. - Appears user friendly and help to more clearly define the goals of the user - I like its simplicity - I think this is a clean, well designed page - This feels like it could work well with dissemination and single-level implementation - Simple concept. I like it. I think it will help knowledge users describe their KT purpose - Love the idea that the suggestions provided are evidence based and a precursor to making yet another tool | |
| Other comments | | |
|  | 1. Prescriptive nature of Explore – may not fit all conditions/circumstances (i.e., how will it handle multiple outcomes, purpose, knowledge users?  - What if the user would like to select more than one options for any of these variables? Would they go through multiple times or can they do this in one step choosing multiple options from the drop-down menu? - What if they have multiple knowledge users or goals? Would they be instructed to use the tool multiple times? - Will this layout work if a person selects multiple outcomes? or should you specify a primary outcome? - Often there are multiple criteria under one option however. How is that handled? For example, would a separate KT purpose statement have to be generated for different KT products/tools? If I wanted to disseminate clinical practice guidelines, patient decision aid and a policy brief to the same KUs, scope/setting and anticipated outcome, can I choose multiple responses or do I have to generate three different purpose statements, one per KT tool? - what if people are not sure of what to do, or wish to pursue multiple options? - Not sure about the complexity of the algorithm for complex implementation within several layers of a health care system, and several target audiences within each; similarly with scale. - It is very prescriptive and wonder how modifiable the Explore page would be - may I select >1 knowledge user, will this pigeonhole me to only selecting items from the drop down. I understand that there are multiple options and "other" category, but worry about leading the user. - May not fit all conditions/circumstances - but forces to consider possible gaps and reflection = good  1. Structure of the KaT framework  - The positioning of KT purpose evaluate is a bit confusing relative to where it fell in the wheel (which implied it came later after roll out). - I'm not sure that "evaluate" is the right word for a KU-focused site, "check" or "assess" might be better. - For patient-facing tool consider adding KT Purpose = to provide patient values and perspective on a topic. - I think you should change the orientation of "develop" in the wheel so the letters aren't upside down. - Overall, I think IKT is done very poorly, KUs invited to attend entire research meetings that are run according to the needs of scientists. For that reason, I'm hesitant to give IKT unqualified support even though I think meaningful involvement of patients and end users throughout the research process is essential  1. KT tools and products repository  - Are there other KT products or tools that could be included or is this a limited subset. Some users may have trouble understanding what each of these is so a glossary or info button may be useful - Patient tools need to be broader than exclusively 'patient decision aids' - there are other KT tools for patients that are not decision aids.  1. The need for definitions and a glossary  - I think a very comprehensive easy to navigate glossary would be necessary to make this function as planned - Some users may be unclear as to the differences between certain response options (e.g. change practice vs. change behaviour). A quick description of each may be helpful. - it may help to offer a "hover over" option where users can get a one or two sentence explanation of what each term means in case they are less familiar with the jargon. - My only concern is that the different activities - e.g., implementation, dissemination - sometimes have blurred lines, so the user and the developer need to be on the same page about definitions. - I could be useful for knowledge users to have access to definitions of the terms used, namely in the KT purpose section. Some may know what they want to achieve but may not be familiar with the KT vocabulary. For the KT product/tool, the options we see on the screen are they the only possible ones? Again, maybe definitions or examples of the KT tools categories could be useful for knowledge users to select the one that applies best to their needs. - Needs help menus because "KT purpose" is not a well-known term.  1. Format  - Suggest keeping the background statement (in blue) visible - I think that the design of the interface is secondary as motivated users will learn how to use it.  1. Other themes:  - Might not be engaging enough for citizen and patient users. Can it be preceded by an 'explainer video'? - My one concern is will it include other KT targets like community settings and preventive interventions? - User research would be helpful to identify a greater range of potential choices for each field, the best way to represent complex purposes (e.g., selecting multiple options for a field vs. generating separate purpose statements), and the possible utility of customizable options for each field - What about mechanisms? Example, practice facilitation, academic detailing, etc. - Depending on the user, they may need some information before they get to this step to understand why they need this - While this seems like a great idea, I'm unsure if we know enough at time about the value of KT models, frameworks and theories to guide end-users throughout the process - I confess that I continue to the resist the use of the term "Knowledge" as though this is the primary drive of practice improvement but I understand that this likely reflects cultural and disciplinary difference. | |

**Appendix M**

Survey respondents’ perceptions of the ACTION PLAN page of the conceptual Knowledge-activated Tools (KaT) platform and corresponding mean Likert scale score

| **ACTION PLAN page of the conceptual KaT platform** | | | **Mean (SD)** |
| --- | --- | --- | --- |
| I like the idea that the ACTION PLAN would provide customized information to platform users | | | 4.65 (0.546) |
|  | | - Customization is great | |
| I would find the table of existing KT tools a useful feature of the KaT platform | | | 4.55 (0.640) |
|  | - I really like the idea of being able to see existing tools. - the table of KT tools would need to be regularly updated - Some relevant existing tools might be overlooked, for instance because of language - Perhaps the list of existing KT tools can be provided only if the user clicks on a link (if interested). Also, more information (a high level description or screen shot) of the existing tools would also be useful - In practice I don’t think anyone would upload a new tool right then and there. Maybe a different part of the portal gives them an option to upload tools and there is a general link to KU-provided tools here | | |
| It’s a good idea to show the quality* rating for each of the suggested KT Tools | | | 4.41 (0.743) |
|  | - I think may be useful to get a wide perspective on quality and relevance since it may vary by disciplines - The quality rating is essential (and should have an underscore link to see the criteria used to assess quality) - I'd want to know how the quality rating is determined; also not sure how customized this info would be to my needs - Love idea of reviewing existing tools. Concerned about 'usual' criteria for quality that may not include pragmatism. - The quality of existing tools will be driven by context, i.e. it might work well in some situations and not others - Also quality is in the eye of the user, not the provider, would suggest not to be too prescriptive | | |
| It’s a good idea to show the relevance* rating for each of the suggested KT tools | | | 4.30 (0.776) |
|  | | - The relevance and quality ratings are useful as long as they are valid (i.e., well correlated with actual quality and relevance). This tool seems to be training as well as building the action plan (e.g., teaching people about evaluation, sustainability and scalability. I wonder if the training functions would be better off in a separate tool - The relevance seems like something I would need to judge for myself so not so relevant here - Relevance seems particularly hard to automate - For relevance, it would be nice to know why it was selected as relevant - I doubt you'll be able to assess relevance in a way that applies across contexts   Comments about both quality and relevance   - I rated quality and relevance neutral because I wasn’t sure how they would be rated. - Would you be open to crowd Sourcing the quality & relevance rating system (similar to NCI's GEM)? This tool would be helpful across a wide audience and I think may be useful to get a wide perspective on quality and relevance since it may vary by disciplines - I wonder that you need to think of the rating in terms of practicality? - Assure that whatever appraisal tool you apply, takes external validity into account, otherwise you recreate the same problem that KT has with all evidence use - Lots of value laden comments in this bit, quality might not be as important as sensitivity to context, and relevance is determined by the choices made during the purpose definition. I suggest that a range of options are made available and as people make decisions they can see which methods are either moved up or down a ranked list - It would be helpful to be able to click to see how each tool met or did not met the criteria for quality and relevance - Quality and relevance is certainly relative, and I'm not sure how this is rated.. or the quality received a 3.5/5 stars - A clear definition of quality and relevance would have to be provided - One concern is the potential for too many tools being listed under the Dissemination Plan--it could become overwhelming, although the quality and relevance ratings will help to choose the appropriate resource - Regarding the assessment of quality and relevance, this might be difficult to achieve given the lack of universal tool to assess the quality of knowledge tools and the fact that relevance is highly subjective. I would not prioritize this feature of the platform, but rather try to be exhaustive in the listing of existing KT tools (for instance, having an up-to-date database of such tools) - I'm not sure that the relevance and quality of the tool would always be interpreted the same way by all knowledge users - what is relevant to one may not be to another even with the same purpose - It will be a challenge to include and rate all existing tools, but a very relevant and worthwhile one. I suppose the difficulty may lie where researchers have promised a 'product' as part of a grant application and then feel duty bound to create another, even if others already exist.... - Challenge will be in how quality and relevance are determined. These could change depending on the who and where the KT tools will be used - Unclear what processes will be used to determine quality and relevance - Would need greater clarity on the process by which quality, relevance and sustainability are operationalized and measured - Quality and relevance of KT tools may be very situation dependent - unsure how this can be generalized - Be very clear on how quality was developed and how up to date the tools are - Should say who is the one who decides the relevance and quality of existing tools. Is it only experts? It is interesting to think about how user experience might be part of the rating (like online restaurant reviews) | |
| It’s important to provide platform users with an option to develop a new KT tool if they wish | | | 4.32 (0.775) |
|  | | - I would suggest including here an option to guide adaptation of an existing tool - I wasn't sure if the action plan would be generated if the respondent opted to use an existing tool (i.e., to adapt that tool); it seems like it would be as important to support adaptation as development of a new tool | |
| It’s important to provide platform users with an option to learn about integrated KT (IKT) | | | 4.38 (0.719) |
|  | | - Learning about IKT different than doing. The issue with IKT at this stage, is that the true co-creation opportunity may have already occurred (but something is better than nothing!) - I don't know what 'integrated KT' means, so it was hard to answer that question - Further, I'm not too sure what integrated KT means, even with the definition below. I'm in the US, so this may mean something different though it seems the goal is to be user friendly for everyone - I think that iKT is extremely important - but again not sure it would be understood the same way by all knowledge users - and it is difficult so it might have the potential to be misunderstood what is really involved - IKT: differentiate between what I call 'participatory implementation' and real 'participatory research'. The former is what usually happens when KT stakeholders come together to identify and address their own evidence needs; the latter is what happens when they find that none of the extant evidence is appropriate for their needs, so they co-create their own knowledge from scratch. Conceptually, this difference has implications for sustained knowledge use - There are many models of KT not just IKT, so a link to a range might be more inclusive - IKT is necessary knowledge | |
| It’s important to provide platform users with the option to learn about evaluating their KT tool | | | 4.57 (0.606) |
|  | | - Yes to evaluation, this is often missed - Not every KT activity should or can be formally evaluated, sometimes due to resources sometimes due to the fact that the evaluation might costs more than the implementation... | |
| It’s important to provide platform users with the option to learn about the ***sustainability*** of their KT tool | | | 4.45 (0.720) |
| It’s important to provide platform users with the option to learn about the ***scalability*** of their KT tool | | | 4.42 (0.696) |
|  | | - Same feedback for implementation as for scale - this feels like a design tool for low complexity, single target and/or single level interventions, perhaps not so good at complex, multilayer and/or multi target health care systems interventions and scale (?). Also - not sure how this aligns with pre-existing quality improvement infrastructure and this is a major part of sustainment in local/rural environments - I think you'll need to define both sustainability and scalability. It’s also important to convey that one would need evidence of effectiveness prior to considering sustaining or scaling your tool. - Regarding sustainability and scalability, I think that some high-level information is needed to explain what these mean in the context of KT, in addition to providing the user with the option to learn about them for their tool. - Sustainability is multifactorial; the tool is only one component = could mislead users - Would need greater clarity on the process by which sustainability are operationalized and measured | |
| **Other comments** | | | |
|  | | - I won’t use KT tools but as a researcher l really like the presentation of this platform - I love the idea of promoting congruence among different tools by pointing people in the direction of tools that might be appropriate for them - I think the breadth of options and resources here is what is most exciting. As a KT practitioner, I am interested for myself but also for those with whom I collaborate to have access to such guidance - Not sure unsophisticated users will be able to create their own successful tools from on line tool? - I like. I find that items 2-6 of the dissemination plan are visually lost on the page as they come immediately after the table summarizing the dissemination tools - might be good to develop ways to make them stand out more. - It was difficult to evaluate the usefulness of any features besides the existing dissemination tools because this demonstration did not illustrate how those would work. Even with the existing tools, I am wondering about the structure of the database for which that information is pulled. Overall, this feels more like a hub that sends the user out for more information than it does an integrated action plan. - Same feedback for implementation as for scale - this feels like a design tool for low complexity, single target and/or single level interventions, perhaps not so good at complex, multilayer and/or multi target health care systems interventions and scale (?). Also - not sure how this aligns with pre-existing quality improvement infrastructure and this is a major part of sustainment in local/rural environments - Its not clear if the question about developing a new KT tool is a new KT category of tools (CPG, or ways of doing SRs), or if it is a new tool tailored for a study or setting based on an existing category of KT tool. - Consider KT tool as part of the overall strategy. - What about fidelity? - It might be useful to provide examples of how the identified tools have been used by others. I think people will tend towards creating their own if they don't see examples of how the existing tools can/have been used by others. - Would also be helpful to have questions that we should be asking ourselves to tailor to particular audiences and/or sensitive topics (eg. talking to adolescents about sexual health) - (1) I might flip the order of sustainability and scalability here and in the wheel so that scalability comes later. - The importance of the last listed items will depend on their phase/purpose but generally important to have - #3-#7 are essential to the model but could get lost as they look like alternative options to the primary choice of using an existing tool or creating your own tool. I think perhaps these options should go on the next page, so users can really consider each carefully once they complete the step of choosing their tool. the only other addition I have is to better define jargon and especially iKT which is a uniquely Canadian term. - I think developing a new tool is a different issue (methods and people) - hard to reconcile with main stream goal= should be a fully separate module. | |
